# New carboxamide derivatives bearing benzenesulphonamide as a selective COX-II inhibitor: Design, synthesis and structure-activity relationship

**DOI:** 10.1371/journal.pone.0183807

**Published:** 2017-09-18

**Authors:** David Izuchukwu Ugwu, Uchechukwu Chris Okoro, Hilal Ahmad

**Affiliations:** 1 Department of Pure and Industrial Chemistry, University of Nigeria, Nsukka, Nigeria; 2 Department of Chemistry, Indian Institute of Technology, Kanpur, India; Universidade Federal do Rio de Janeiro, BRAZIL

## Abstract

Sixteen new carboxamide derivatives bearing substituted benzenesulphonamide moiety (**7a-p**) were synthesized by boric acid mediated amidation of appropriate benzenesulphonamide with 2-amino-4-picoline and tested for anti-inflammatory activity. One compound **7c** showed more potent anti-inflammatory activity than celecoxib at 3 h in carrageenan-induced rat paw edema bioassay. Compounds **7g** and **7k** also showed good anti-inflammatory activity comparable to celecoxib. Compound **7c** appeared selectivity index (COX-2/COX-1) better than celecoxib. Compound **7k** appeared selectivity index (COX-2/COX-1) a little higher than the half of celecoxib while compound **7g** is non-selective for COX-2. The LD_50_ of compounds **7c, 7g** and **7k** were comparable to celecoxib.

## Introduction

The initial stage of transformation of arachidonic acid to prostanoids are catalysed by cyclooxygenases (COXs). It exists as three distinct isozymes; cyclooxygenase-1 (COX-I), cyclooxygenase-2 (COX-II) and cyclooxygenase-3 (COX-III). Selective COX-II inhibitors are a class of potential anti-inflammatory, analgesic and antipyretic drugs with reduced gastrointestinal (GI) side effects compared to non-selective inhibitors [1–3]. Cyclooxygenase (COX)-2 is one of the rate-limiting enzyme in the production of prostaglandin from arachidonic acid. Non-steroidal anti-inflammatory drugs (NSAIDs) are the competitive inhibitors of cyclooxygenase (COX), the enzyme which mediates the bioconversion of arachidonic acid to inflammatory prostaglandins (PGs). The inhibition of COX-2 gives rise to the anti-inflammatory activity of NSAIDs whereas the undesired side effects arise from inhibition of COX-1 activity. Thus, it was thought that more selective COX-2 inhibitors would have reduced side effects [3]. Based upon a number of selective COX-2 inhibitors (rofecoxib, celecoxib, valdecoxib etc.) were developed as safer NSAIDs with improved gastric safety profile [4]. However, the recent market removal of some COXIBs such as rofecoxib due to its adverse cardiovascular side effects clearly encourages researchers to explore and evaluate alternative templates with COX-2 inhibitory activity [3]. Recognition of new avenues for selective COX-2 inhibitors in cancer chemotherapy and neurological diseases such as Parkinson and Alzheimer’s diseases still continues to attract investigations on the development of COX-2 inhibitors [5]. COX-2 is induced by stimuli such as mitogens, cytokines, growth factors and tumor promoters, and has been elucidated to be up-regulated not only at the sites of inflammation but also in various cancer tissues such as colon, stomach, breast, lung, head and neck including oral cavity [6]. The biosynthesis of prostanoids, which include the prostaglandins (PGs) and thromboxanes, occurs in three steps: (a) the mobilization of a fatty acid substrate, typically arachidonic acid (AA), from membrane phospholipids through the action of a phospholipase A2; (b) biotransformation of AA by cyclooxygenase in a bifunctional action which leads to the generation of unstable PGG2 by the cyclooxygenase reaction, and its immediate conversion into PGH2 by the same enzyme in a peroxidase reaction; (c) the conversion of PGH2 to specific prostanoids through the action of synthases and specific isomerases [6]. The successful inhibition of COX-2 will arrest the synthesis of prostaglandin which has been implicated in varieties of physiological and pathophysiological conditions, including inflammation [7].

Sulphonamides have been the centre of drug structures as they are quite stable and well tolerated in human beings [8]. Sulphonamides constitute an important class of chemotherapeutic agents with applications ranging from their traditional antibacterial agent [8] to anticancer [9], antimalarial [10], anticonvulsant [11], antiretroviral [12], antidiabetic [13], anti-insomnia [14], anti-inflammatory [15], diuretics [16] and antileukemic [17] agents to mention but a few.

Carboxamides are also ubiquitous functionality in drug molecules as pharmacophore [18]. Carboxamides are present in drug molecules used in the blockage of cholesterol synthesis [19], treatment of hypertension and angina [20], blockade of angiotensin-II receptors [21], inhibition of angiotensin converting enzyme [22], treatment of HIV [23], and management of heart disease [24] to mention but a few.

We therefore exploited the synergistic biological properties arising from the successful incorporation of carboxamides in substituted benzenesulphonamides in this report.

## Experimental

### Instrumentation

All reactions requiring inert atmosphere were carried out under nitrogen atmosphere. Drying of solvents was achieved using molecular sieve for 48 h. All reagents were purchased from commercial suppliers, Aldrich, Merck, Fluka, Avra, SD fine and Alfa Aesar. Thin layer chromatography was carried out using silica plates purchased from Avra. The plates were visualized under UV light (popular India). FT-IR spectroscopy of the compounds were run in PerkinElmer Spectrum version 10.03.06 and the bands presented in wavenumber. 1H NMR and 13C NMR spectroscopy were run in DMSOd_6_ and CD_3_OD, unless otherwise stated on either Jeol 500 MHz or 400 MHz. The chemical shifts were reported in part per million with reference to tetramethylsilane. Mass spectroscopy were carried out using micro TOF electrospray time of flight (ESI-TOF) mass spectrometer, sodium formate was used as the calibrant. All experiments were carried out at Prof. Sandeep Verma’s Laboratory, department of Chemistry, Indian Institute of Technology, Kanpur. Melting points were determined using digital melting point apparatus and were uncorrected.

#### General procedure for the synthesis of substituted benzenesulphonamoyl alkanamides (3a-p)

Sodium carbonate (Na_2_CO_3_, 1.590 g, 15 mmol) was added to a solution of amino acids (**2a-h**, 12.5 mmol) in water (15 mL) with continuous stirring until all the solutes had dissolved. The solution was cooled to -5°C and the appropriate benzenesulphonyl chloride (**1a-c**, 15 mmol) was added in four portions over a period of 1 h. The slurry was further stirred at room temperature for about 4 h. The progress of the reaction was monitored using TLC (MeOH/DCM, 1:9). Upon completion, the mixture was acidified using 20% aqueous hydrochloric acid to pH 2. The crystals was filtered via suction and washed with pH 2.2 buffer. The pure products (**3a-p**) were dried over self-indicating fused silica gel in a desiccator.

#### General procedure for the synthesis of *N*-benzoyl derivatives of benzenesulphonamides (5a-f and 5i-n)

Appropriate benzenesulphonamides (**3a-f**, and **3i-n**, 1.0 mmol) was dissolved in NaOH (10%, 10 mL) in a 50 mL round bottom flask. Benzoyl chloride (**8**, 1.1 mmol, 0.2 mL) was transferred into the solution of appropriate benzenesulphonamide and stirred at room temperature. The reaction progress was monitored by TLC (3% MeOH/CH_2_Cl_2_) to the disappearance of the benzenesulphonamide spot. Upon completion, the solution was transferred into a beaker containing crushed ice and then acidified to pH of 3 with concentrated hydrochloric acid. The solid was collected via suction filtration and transferred into a beaker containing CCl_4_ (10 mL) and covered with watch glass boiled for 10 min. the mixture was allowed to cool slightly and then filtered. The products **(5a-f** and **5i**-**n)** obtained were washed with 10–20 mL of CCl_4_ and dried over fused self-indicating silica gel in a dessicator.

#### Boric acid catalysed direct amidation of unactivated carboxylic acid and 2-amino-4-picoline

To a suspension of *N*-benzoyl-substituted-benzenesulphonamides (**5a-f** and **5i**-**n**, 1.0 mmol) in dry toluene (40 mL) equipped with Dean-Stark apparatus for azeotropic removal of water, was added 4-picoline (1.0 mmol) and boric acid (0.1 mmol) at room temperature and then refluxed for 6 h. On completion (as monitored by TLC), reaction mixture was precipitated in to amides by adding about 40 mL *n*-hexane. The carboxamides were obtained via suction filtration, washed with *n*-hexane and dried over fused silica gel or concentrated using rotary evaporator and dried over vacuum in the case of oily products.

***N*-(4-methylpyridin-2-yl)-2-[*N*-(4-nitrobenzenesulfonyl)**-**1-phenylformamido]acetamide (7a)**

Appearance: brownish oil; Yield (0.4471 g, 98.5%), FTIR (KBr, cm^-1^): 3420 (NH), 3064 (C-H aromatic), 2976 (C-H aliphatic), 1701, 1669 (C = O), 1631 (C = N), 1601, 1494, 1454 (C = C), 1334, 1310 (2S = O), 1166, 1123 (SO_2_N), 1091, 1015 (C-N). ^1^H NMR (DMSO-d_6_, 400 MHz) δ: 8.34–8.31 (m, 1H, ArH), 8.24–8.22 (m, 1H, ArH), 8.14–8.09 (m, 2H, ArH), 7.91 (d, J = 6.88 Hz, 2H, ArH), 7.74 (d, J = 8.72 Hz, 2H, ArH), 7.66 (d, J = 5.96 Hz, 2H, ArH), 7.55 (t, J = 7.76 Hz, 1H, ArH), 7.44 (t, J = 7.32 Hz, 2H, ArH), 7.24–7.01 (m, 2H, ArH), 6.43 (s, 2H, ArH), 3.24 (s, 2H, CH_2_), 2.17 (s, 3H, CH_3_). ^13^C NMR (DMSO-d_6_, 400 MHz) δ: 173.9, 168.3 (C = O), 157.7, 151.8, 149.5, 147.2, 142.0, 137.7, 129.8, 129.4, 129.0, 128.6, 128.3, 126.8, 124.5, 114.1, 110.4 (fifteen aromatic carbons), 58.9, 21.6 (two aliphatic carbons). HRMS (ESI-TOF, m/z): 454.0958 (M^+^), calculated, 454.0947.

**(2S)-*N*-(4-methylpyridin-2-yl)-2-[*N*-(4-nitrobenzenesulfonyl)**-**1-phenylformamido]-3-phenyl propanamide (7b)**

Appearance: pale brown solids; Yield (0.5304 g, 97.5%),mp, 156.10–156.80°C, FTIR (KBr, cm^-1^): 3383 (NH), 3034 (C-H aromatic), 2964 (C-H aliphatic), 1773, 1661 (C = O), 1624 (C = N), 1602, 1575, 1446, 1412 (C = C), 1525 (NO_2_), 1353, 1304 (2S = O), 1199, 1168 (SO_2_N), 1093, 1044, 1013 (C-N). ^1^H NMR (DMSO-d_6_, 400 MHz) δ: 8.36–8.30 (m, 3H, ArH), 8.16 (d, J = 8.72 Hz, 1H, ArH), 8.02–7.98 (m, 3H, ArH), 7.90 (d, J = 7.32 Hz, 1H, ArH), 7.71 (d, J = 5.96 Hz, 2H, ArH), 7.58 (t, J = 7.56 Hz, 1H, ArH), 7.45 (t, J = 7.10 Hz, 2H, ArH), 7.14–7.08 (m, 2H, ArH), 6.72 (s, 1H, NH), 6.46–6.43 (m, 2H, ArH), 3.89–3.85 (m, 1H, CH), 2.96 (dd, J = 4.60, 4.56 Hz, 1H, CH_a_ of CH_2_), 2.71 (dd, J = 9.60, 10.08 Hz, 1H, CH_b_ of CH_2_), 2.15 (s, 3H, CH_3_). ^13^C NMR (DMSO-d_6_, 400 MHz) δ: 170.7, 167.9 (C = O), 157.4 (C = N), 152.0, 149.9, 149.9, 147.0, 146.2, 142.0, 133.3, 129.8, 129.1, 128.9, 128.7, 128.2, 124.9, 124.7, 124.6, 114.2, 110.4 (seventeen aromatic carbons), 48.9, 44.5, 21.5 (three aliphatic carbons). HRMS (ESI-TOF, m/z): 562.0107 (M+NH_4_), calculated, 562.0110.

**(2S)-3-(*1H*-indol-2-yl)-*N*-(4-methylpyridin-2-yl)-2-[*N*-(4-nitrobenzenesulfonyl)**-**1-phenylformamido]propanamide (7c)**

Appearance: yellowish-brown solids; Yield (0.5799 g, 99.4%), mp, 100.50–100.80°C, FTIR (KBr, cm^-1^): 3415, 3365 (NH), 3087 (C-H aromatic), 2891 (C-H aliphatic), 1702, 1669 (C = O), 1623 (C = N), 1601, 1491 (C = C), 1528 (NO_2_), 1350, 1311 (2S = O), 1163, 1121 (SO_2_N), 1093 (C-N). ^1^H NMR (DMSO-d_6_, 500 MHz) δ: 10.6664 (s, 1H, NH of indole), 8.58 (s, 1H, ArH), 7.91 (d, J = 6.90 Hz, 1H, ArH), 7.81 (d, J = 8.60 Hz, 2H, ArH), 7.75–7.70 (m, 1H, ArH), 7.58 (t, J = 7.45 Hz, 1H, ArH), 7.48–7.45 (m, 3H, ArH), 7.25–7.20 (m, 1H, ArH), 7.12 (t, J = 7.40 Hz, 1H, ArH), 7.05 (d, J = 8.00 Hz, 1H, ArH), 6.99–6.98 (m, 1H, ArH), 6.88–6.82 (m, 2H, ArH), 6.32 (d, J = 5.15 Hz, 1H, ArH), 6.26 (s, 1H, ArH), 5.97 (s, 1H, NH), 3.88 (t, J = 4.28 Hz, 1H, CH-C = O), 3.04 (dd, J = 4.60, 4.00 Hz, 1H, CH_a_ of CH_2_), 2.78 (dd, J = 10.30, 10.30 Hz, 1H, CH_a_, CH_2_), 2.11 (s, 3H, CH_3_). ^13^C NMR (DMSO-d_6_, 500 MHz) δ: 173.5, 167.9 (C = O), 159.7 (C = N), 148.9, 148.7, 146.5, 136.5, 133.4, 129.8, 129.4, 129.1, 128.7, 127.4, 126.9, 125.9, 124.8, 123.8, 121.2, 118.8, 118.2, 113.9, 111.7, 109.3, 108.9 (twenty one aromatic carbons), 57.2, 28.4, 21.2 (three aliphatic carbons). HRMS (ESI-TOF, m/z): 583.1236 (M^+^), 583.1526.

**(2S)-4-Methyl-*N*-(4-methylpyridin-2-yl)-2-[*N*-(4-nitrobenzenesulfonyl)**-**1-phenylformamido]pentanamide (7d)**

Appearance: yellowish oil; Yield (0.4989 g, 97.8%), FTIR (KBr, cm^-1^): 3333 (NH), 3106 (C-H aromatic), 2957, 2871 (C-H aliphatic), 1691, 1671 (C = O), 1619 (C = N), 1603, 1583, 1492, 1452 (C = C), 1530 (NO_2_), 1316, 1301 (2S = O), 1172, 1148 (SO_2_N), 1093, 1071, 1025 (C-N). ^1^H NMR (DMSO-d_6_, 500 MHz) δ: 8.36–8.24 (m, 2H, ArH), 8.12–8.05 (m, 2H, ArH), 7.97 (d, J = 8.72 Hz, 2H, ArH), 7.90 (d, J = 7.76 Hz, 1H, ArH), 7.66 (m, 1H, ArH), 7.55 (t, J = 7.32 Hz, 1H, ArH), 7.43 (t, J = 7.56 Hz, 1H, ArH), 7.20–7.06 (m, 2H, ArH), 3.67 (t, J = 7.32 Hz, 1H, CH-C = O), 2.17 (s, 3H, CH_3_), 1.92–1.87 (m, 1H, CH), 1.63–1.55 (m, 1H, CH_a_ of CH_2_), 1.41–1.37 (m, 1H, CH_b_ of CH_2_). ^13^C NMR (DMSO-d_6_, 400 MHz) δ: 174.4, 168.2, 156.9, 152.7, 149.8, 147.3, 140.8, 129.8, 129.4, 128.9, 128.7, 125.8, 124.6, 114.2, 110.8 (thirteen aromatic carbons), 55.4, 41.5, 24.5, 23.2, 21.5 (five aliphatic carbons). HRMS (ESI-TOF, m/z): 511.2299 (M+H), calculated, 510.1573.

**(2S)-3-Methyl-*N*-(4-methylpyridin-2-yl)-2-[*N*-(4-nitrobenzenesulfonyl)**-**1-phenylformamido]pentanamide (7e)**

Appearance: pale yellow solids; Yield (0.5009 g, 98.2%), 110.90–111.40°C, FTIR (KBr, cm^-1^): 3422 (NH), 3071 (C-H aromatic), 2962 (C-H aliphatic), 1673, 1662 (C = O), 1618 (C = N), 1601, 1496, 1470 (C = C), 1521 (NO_2_), 1332, 1311 (2S = O), 1169, 1136 (SO_2_N), 1093, 1051, 1016 (C-N). ^1^H NMR (DMSO-d_6_, 500 MHz) δ: 8.34 (d, J = 9.15 Hz, 2H, ArH), 7.98 (d, J = 8.60 Hz, 2H, ArH), 7.69 (d, J = 5.15 Hz, 1H, ArH), 7.15 (m, 5H, ArH), 6.30 (d, J = 5.15 Hz, 1H, ArH), 6.23 (s, 1H, ArH), 5.91 (s, 1H, NH), 3.58 (d, J = 5.70 Hz, 1H, CH-C = O), 2.10 (s, 3H, CH_3_), 1.69–1.65 (m, 1H, CH), 1.36–1.30 (m, 1H, CH_a_ of CH_2_), 1.11–1.03 (m, 1H, CH_b_ of CH_2_), 0.82–0.73 (m, 6H, 2CH_3_). ^13^C NMR (DMSO-d_6_, 500 MHz) δ: 172.6, 165.4 (C = O), 159.9, 158.5, 149.9, 148.4, 147.2, 146.8, 146.2, 129.4, 128.7, 125.9, 124.8, 113.9, 108.9 (thirteen aromatic carbons), 61.1, 37.4, 24.9, 21.1, 15.9, 11.5 (six aliphatic carbons). HRMS (ESI-TOF, m/z): 510.1582 (M^+^), calculated, 510.1583.

**(2S)-3-Methyl-*N*-(4-methylpyridin-2-yl)-2-[*N*-(4-nitrobenzenesulfonyl)**-**1-phenylformamido]butanamide (7f)**

Appearance: yellow solid; Yield (0.4869 g, 98.2%), 143.30–143.90°C, FTIR (KBr, cm^-1^): 3418 (NH), 2964 (C-H aliphatic), 1672, 1663 (C = O), 1620 (C = N), 1604, 1495 (C = C), 1520 (NO_2_), 1356, 1311 (2S = O), 1169, 1140 (SO_2_N), 1093, 1047, 1016 (C-N). ^1^H NMR (DMSO-d_6_, 400 MHz) δ: 8.31 (d, J = 8.72 Hz, 2H, ArH), 7.78 (d, J = 9.16 Hz, 2H, ArH), 7.89 (d, J = 7.32 Hz, 1H, ArH), 7.67 (d, J = 5.46 Hz, 1H, ArH), 7.56 (t, J = 7.56 Hz, 1H, ArH), 7.44 (t, J = 7.56 Hz, 1H, ArH), 7.27–7.009 (m, 2H, ArH), 6.31 (d, J = 5.52 Hz, 1H, ArH), 6.26 (s, 1H, ArH), 6.0654 (s, 1H, NH), 3.51 (d, J = 5.96 Hz, 1H, CH-C = O), 2.09 (s, 3H, CH_3_), 1.96–1.89 (m, 1H. CH), 0.80–0.75 (m, 6H, 2CH_3_). ^13^C NMR (DMSO-d_6_, 500 MHz) δ: 172.8, 168.1 (C = O), 159.4, 149.8, 149.1, 147.3, 145.9, 129.8, 129.4, 129.1, 128.7, 125.8, 124.7, 113.9, 109.2 (thirteen aromatic carbons), 62.3, 30.9, 21.2, 19.6, 18.3 (five aliphatic carbons). HRMS (ESI-TOF, m/z): 497.0419 (M+H), calculated, 497.0422.

**(2S)-4-Hydroxy-*N*-(4-methylpyridin-2-yl)**-**1-(4-nitrobenzenesulfonyl)pyrrolidine-2-carboxamide (7g)**

Appearance: crystalline yellow; Yield (0.4061 g, 100%), 100.20–100.60°C, FTIR (KBr, cm^-1^): 3414 (OH), 3323 (NH), 3107 (C-H aromatic), 2953 (C-H aliphatic), 1670 (C = O), 1635 (C = N), 1606, 1491 (C = C), 1530 (NO_2_), 1352, 1308 (2S = O), 1199, 1161 (SO_2_N), 1093, 1010 (C-N, C-O). ^1^H NMR (DMSO-d_6_, 400 MHz) δ: 8.35 (d, J = 9.16 Hz, 2H, ArH), 8.02 (d, J = 8.72 Hz, 2H, ArH), 7.69 (d, J = 5.52 Hz, 1H, ArH), 6.28 (d, J = 5.48 Hz, 1H, ArH), 6.22 (s, 1H, ArH), 5.82 (s, 1H, NH), 4.16 (s, 1H, OH), 4.09 (t, J = 7.80 Hz, 1H, CH-C = O), 3.19 (d, J = 5.45 Hz, 2H, CH_2_N), 2.08 (s, 3H, CH_3_), 2.03–1.98 (m, 1H, CH-OH), 1.93–1.86 (m, 2H, CH_2_). ^13^C NMR (DMSO-d_6_, 400 MHz) δ: 173.8 (C = O), 160.1, 150.3, 148.2, 147.2, 143.6, 129.5, 124.8, 113.9, 108.8 (nine aromatic carbons), 69.0, 60.6, 57.1, 21.1 (four aliphatic carbons). HRMS (ESI-TOF, m/z): 407.1023 (M+H), calculated, 407.1027.

**(2S)-*N*-(4-methylpyridin-2-yl)**-**1-(4-nitrobenzenesulfonyl)pyrrolidine-2-carboxamide (7h)**

Appearance: off-yellow crystals; Yield (0.3900 g, 99.9%), 156.70–157.20°C, FTIR (KBr, cm^-1^): 3265 (NH), 3009 (C-H aromatic), 2981, 2893 (C-H aliphatic), 1678 (C = O), 1641 (C = N), 1602, 1492, 1449 (C = C), 1320, 1306 (2S = O), 1183, 1163 (SO_2_N), 1088, 1045 (C-N). ^1^H NMR (DMSO-d_6_, 400 MHz) δ: 8.35 (d, J = 9.16 Hz, 2H, ArH), 8.05 (d, J = 9.16 Hz, 2H, ArH), 7.68 (d, J = 5.48 Hz, 1H, ArH), 6.34 (d, J = 5.48 Hz, 1H, ArH), 6.31 (s, 1H, ArH), 4.15 (dd, J = 3.20, 3.68 Hz, 1H, CH-C = O), 3.37–3.34 (m, 1H, CH_a_ of CH_2_N), 3.24–3.18 (m, 1H, CH_b_ of CH_2_N), 2.12 (s, 3H, CH_3_), 1.96–1.91 (m, 1H, CH of CH_2_), 1.86–1.75 (m, 2H, CH_2_), 1.66–1.59 (m, 1H, CH of CH_2_). ^13^C NMR (DMSO-d_6_, 400 MHz) δ: 174.1 (C = O), 159.1, 150.3, 149.7, 144.9, 143.9, 129.4, 125.1, 113.9, 109.5 (nine aromatic carbons), 61.5, 48.9, 31.0, 24.7, 21.3 (five aliphatic carbons). HRMS (ESI-TOF, m/z): 389.0603 (M-H), calculated, 389.0604.

**2-[*N*-(4-methylbenzenesulfonyl)**-**1-phenylformamido]-*N*-(4-methylpyridin-2-yl)acetamide (7i)**

Appearance: white crystals; Yield (0.4201 g, 99.3%), mp, 137.10–137.60°C, FTIR (KBr, cm^-1^): 3311 (NH), 3039 (C-H aromatic), 2091 (C-H aliphatic), 1703, 1664 (C = O), 1644 (C = N), 1602, 1579, 1496, 1426 (C = C), 1392, 1329 (2S = O), 1185, 1162 (SO_2_N), 1093, 1018 (C-N). ^1^H NMR (DMSO-d_6_, 500 MHz) δ: 7.91 (d, J = 8.60 Hz, 3H, ArH), 7.72 (d, J = 5.75 Hz, 1H, ArH), 7.64 (d, J = 8.00 Hz, 1H, ArH), 7.57 (t, J = 7.45 Hz, 1H, ArH), 7.45 (t, J = 7.73 Hz, 2H, ArH), 7.33–7.27 (m, 2H, ArH), 6.32 (d, J = 5.15 Hz, 1H, ArH), 6.28 (s, 1H, ArH), 6.15 (s, 1H, NH), 3.46 (s, 2H, CH_2_), 2.32 (s, 3H, CH_3_-Ar), 2.11 (s, 3H, CH_3_-Py). ^13^C NMR (DMSO-d_6_, 500 MHz) δ: 171.3, 168.2 (C = O), 159.7 (C = N), 148.8, 146.3, 143.1, 138.3, 133.2, 131.7, 129.9, 129.8, 129.0, 128.7, 127.1, 113.9, 109.1 (thirteen aromatic carbons), 44.8, 21.5, 21.2 (three aliphatic carbons). HRMS (ESI-TOF, m/z): 423.0782 (M^+^), calculated, 423.0783.

**(2S)-2-[*N*-(4-methylbenzenesulfonyl)**-**1-phenylformamido]-*N*-(4-methylpyridin-2-yl)-3-phenyl propanamide (7j)**

Appearance: off-white solids; Yield (0.5009 g, 97.6%), mp, 91.50–92.30°C, FTIR (KBr, cm^-1^): 3267 (NH), 3062 (C-H aromatic), 1700, 1679 (C = O), 1641 (C = N), 1599, 1494, 1452 (C = C), 1383, 1301 (2S = O), 1174, 1157 (SO_2_N), 1091, 1025 (C-N). ^1^H NMR (DMSO-d_6_, 500 MHz) δ: 7.92 (d, J = 7.45 Hz, 3H, ArH), 7.71 (d, J = 5.20 Hz, 2H, ArH), 7.56 (t, J = 7.45 Hz, 2H, ArH), 7.45 (t, J = 7.43 Hz, 4H, ArH), 7.22–7.08 (m, 5H, ArH), 6.35 (d, J = 5.15 Hz, 1H, ArH), 6.33 (s, 1H, ArH), 5.47 (s, 1H, NH), 3.77 (t, J = 5.75 Hz, 1H, CH-C = O), 2.90 (dd, J = 5.75, 5.75 Hz, 1H, CH_a_ of CH_2_), 2.72 (dd, J = 8.05, 8.55 Hz, 1H, CH_b_ of CH_2_), 2.26 (s, 3H, CH_3_-Ar), 2.12 (s, 3H, CH_3_-Py). ^13^C NMR (DMSO-d_6_, 500 MHz) δ: 173.7, 168.3 (C = O), 159.2 (C = N), 149.6, 145.1, 142.7, 138.7, 137.7, 133.1, 131.9, 129.7, 129.4, 128.9, 128.7, 128.6, 126.9, 125.9, 113.9, 109.5 (seventeen aromatic carbons), 58.4, 38.6, 21.4, 21.2 (four aliphatic carbons). HRMS (ESI-TOF, m/z): 512.0759 (M-H), calculated, 512.0762.

**(2S)-3-(*1H*-indol-2-yl)-2-[*N*-(4-methylbenzenesulfonyl)**-**1-phenylformamido]-*N*-(4-methylpyridin-2-yl)propanamide (7k)**

Appearance: creamy solids; Yield (0.5502 g, 99.6%), mp, 114.40–114.70°C, FTIR (KBr, cm^-1^): 3421, 3264 (2NH), 3020 (C-H aromatic), 1692, 1684 (C = O), 1641 (C = N), 1599, 1494, 1454 (C = C), 1326, 1299 (2S = O), 1183, 1148 (SO_2_N), 1067, 1025 (C-N). ^1^H NMR (DMSO-d_6_, 500 MHz) δ: 10.73 (s, 1H, NH of indole), 8.01 (s, 1H, ArH), 7.91 (d, J = 8.60 Hz, 1H, ArH), 7.71 (d, J = 5.20 Hz, 1H, ArH), 7.58 (t, J = 7.45 Hz, 1H, ArH), 7.44 (m, 3H, ArH), 7.21 (m, 3H, ArH), 7.12 (t, J = 8.60 Hz, 3H, ArH), 7.01–6.99 (m, 2H, ArH), 6.88 (t, J = 8.60 Hz, 1H, ArH), 6.32 (d, J = 5.15 Hz, 1H, ArH), 6.27 (s, 1H, ArH), 3.81 (t, J = 4.30 Hz, 1H, CH-C = O), 3.01 (dd, J = 6.30, 6.30 Hz, 1H, CH_a_ of CH_2_), 2.81 (dd, J = 8.00, 7.40 Hz, CH_b_ of CH_2_), 2.27 (m, 3H, CH_3_-Ar), 2.11 (s, 3H, CH_3_-Py). ^13^C NMR (DMSO-d_6_, 500 MHz) δ: 173.3, 167.7 (C = O), 159.6 (C = N), 148.8, 146.3, 142.7, 133.3, 129.8, 129.7, 129.4, 129.1, 128.7, 127.5, 126.8, 125.9, 124.4, 121.3, 118.8, 118.4, 113.9, 111.9, 109.5, 109.0 (twenty one aromatic carbons), 57.2, 28.8, 21.6, 21.5 (four aliphatic carbons). HRMS (ESI-TOF, m/z): 553.1335 (M+H), calculated, 553.1339.

**(2S)-4-Methyl-2-[*N*-(4-methylbenzenesulfonyl)**-**1-phenylformamido]-*N*-(4-methylpyridin-2-yl) pentanamide (7l)**

Appearance: yellowish oil; Yield (0.4689 g, 97.8%), FTIR (KBr, cm^-1^): 3196 (NH), 3063 (C-H aromatic), 2959 (C-H aliphatic), 1692, 1675 (C = O), 1642 (C = N), 1600, 1493, 1451 (C = C), 1383, 1316 (2S = O), 1161 (SO_2_N), 1093, 1070, 1025 (C-N). ^1^H NMR (DMSO-d_6_, 500 MHz) δ: 7.93–7.91 (m, 3H, ArH), 7.69 (d, J = 5.52Hz, 1H, ArH), 7.61 (d, J = 8.24 Hz, 1H, ArH), 7.57–7.53 (m, 1H, ArH), 7.46–7.42 (m, 3H, ArH), 7.28–7.26 (d, J = 7.80 Hz, 1H, ArH), 7.21–7.17 (t, J = 7.34 Hz, 1H, ArH), 7.12–7.09 (t, J = 7.10 Hz, 1H, ArH), 6.37 (s, 2H, ArH), 3.59–3.58 (t, J = 6.88 Hz, 1H, CH-C = O), 2.31–2.24 (m, 3H, CH_3_-Ar), 2.12 (s, 3H, CH_3_-Py), 1.59–1.52 (m, 1H, CH), 1.34 (t, J = 6.88 Hz, 2H, CH_2_), 0.79–0.65 (m, 6H, 2CH_3_). ^13^C NMR (DMSO-d_6_, 500 MHz) δ: 174.7, 168.4 (C = O), 158.8, 150.3, 144.1, 142.9, 138.8, 133.1, 131.9, 129.8, 129.4, 128.9, 128.7, 127.1, 125.8, 113.9, 109.8 (fifteen aromatic carbons), 54.9, 24.4, 23.1, 21.7, 21.3, 15.9 (six aliphatic carbons). HRMS (ESI-TOF, m/z): 480.1989 (M+H), calculated, 480.1992.

**(2S)-3-Methyl-2-[*N*-(4-methylbenzenesulfonyl)**-**1-phenylformamido]-*N*-(4-methylpyridin-2-yl) pentanamide (7m)**

Appearance: off-white solids; Yield (0.4709 g, 98.3%), mp, 91.20–91.80°C, FTIR (KBr, cm^-1^): 3268 (NH), 3012 (C-H aromatic), 2965 (C-H aliphatic), 1692, 1679 (C = O), 1641 (C = N), 1598, 1496 (C = C), 1383, 1301 (2S = O), 1161, 1123 (SO_2_N), 1093, 1024 (C-N). ^1^H NMR (DMSO-d_6_, 500 MHz) δ: 7.92 (d, J = 6.88 Hz, 4H, ArH), 7.68 (d, J = 5.96 Hz, 2H, ArH), 7.61 (d, J = 8.24 Hz, 2H, ArH), 7.54 (t, J = 7.34 Hz, 2H, ArH), 7.43 (t, J = 7.56 Hz, 4H, ArH), 7.24 (d, J- 8.24 Hz, 2H, ArH), 6.82 (s, 1H, ArH), 6.36 (d, J = 5.52 Hz, 3H, ArH), 3.45 (d, J = 5.52 Hz, 1H, CH-C = O), 2.29–2.23 (m, 3H, CH_3_-Ar), 2.12 (s, 3H, CH_3_-Py), 1.66–1.60 (m, 1H, CH), 1.37–1.31 (m, 1H, CH_a_ of CH_2_), 1.09–1.02 (m, 1H, CH_a_ of CH_2_), 0.77–0.67 (m, 6H, 2CH_3_). ^13^C NMR (DMSO-d_6_, 500 MHz) δ: 173.7, 168.6 (C = O), 158.8, 150.4, 143.9, 142.8, 138.8, 133.0, 132.1, 129.7, 128.9, 127.2, 125.8, 113.9, 109.8 (thirteen aromatic carbons), 61.1, 37.7, 24.4, 21.4, 21.3, 16.0, 11.6 (seven aliphatic carbons). HRMS (ESI-TOF, m/z): 479.1888 (M^+^), calculated, 479.1879.

**(2S)-3-Methyl-2-[*N*-(4-methylbenzenesulfonyl)**-**1-phenylformamido]-*N*-(4-methylpyridin-2-yl) butanamide (7n)**

Appearance: yellowish oil; Yield (0.4554 g, 97.9%), FTIR (KBr, cm^-1^): 3322 (NH), 3064 (C-H aromatic), 2965 (C-H aliphatic), 1691, 1673 (C = O), 1623 (C = N), 1601, 1493, 1451 (C = C), 1386, 1316 (2S = O), 1161 (SO_2_N), 1093, 1071, 1025 (C-N). ^1^H NMR (DMSO-d_6_, 400 MHz) δ: 7.92–7.90 (m, 2H, ArH), 7.70 (d, J = 8.24 Hz, 1H, ArH), 7.61 (d, J = 8.24 Hz, 1H, ArH), 7.55 (t, J = 7.32 Hz, 1H, ArH), 7.44 (t, J = 7.80 Hz, 3H, ArH), 7.32–7.26 (m, 2H, ArH), 6.35 (d, J = 5.52 Hz, 1H, ArH), 6.32 (s, 1H, ArH), 3.42 (d, J = 5.52 Hz, 1H, CH-C = O), 2.31–2.25 (m, 3H, CH_3_-Ar), 2.12 (s, 3H, CH_3_-Py), 1.92–1.87 (m, 1H, CH), 0.79–0.74 (m, 6H, 2CH_3_). ^13^C NMR (DMSO-d_6_, 500 MHz) δ: 173.4, 168.3 (C = O), 159.1 (C = N), 149.7, 144.9, 142.8, 138.8, 133.2, 131.8, 129.8, 129.0, 127.1, 126.9, 113.9, 109.5 (thirteen aromatic carbons), 62.1, 30.9, 21.4, 21.2, 19.6, 18.4 (six aliphatic carbons). HRMS (ESI-TOF, m/z): 466.1392 (M+H), calculated, 466.1393.

**(2S)-4-Hydroxy**-**1-(4-methylbenzenesulfonyl)-*N*-(4-methylpyridin-2-yl)pyrrolidine-2-carboxamide (7o)**

Appearance: brownish oil; Yield (0.3754 g, 100%), FTIR (KBr, cm^-1^): 3421 (OH), 3362 (NH), 3028 (C-H aromatic), 2967, 2891 (C-H aliphatic), 1682 (C = O), 1612 (C = N), 1601, 1573, 1455 (C = C), 1342, 1312 (2S = O), 1176, 1121 (SO_2_N), 1092, 1073 (C-N). ^1^H NMR (DMSO-d_6_, 400 MHz) δ: 7.70 (d, J = 5.48 Hz, 1H, ArH), 7.63 (d, J = 8.24 Hz, 2H, ArH), 7.35 (d, J = 7.80 Hz, 2H, ArH), 6.34 (d, J = 5.52 Hz, 1H, ArH), 6.30 (s, 1H, ArH), 6.18 (s, 1H, NH), 4.17 (s, 1H, OH), 4.00 (t, J = 7.80 Hz, 1H, CH-C = O), 3.43–3.39 (m, 1H, CH-OH), 3.03 (d, J = 5.25 Hz, 2H, CH_2_N), 2.34 (s, 3H, CH_3_-Ar), 2.11 (s, 3H, CH_3_-Py), 1.91–1.85 (m, 2H, CH_2_). ^13^C NMR (DMSO-d_6_, 500 MHz) δ: 173.9 (C = O), 159.2 (C = N), 149.5, 145.4, 143.7, 135.0, 130.1, 129.4, 128.7, 127.9, 114.0, 109.3 (eleven aromatic carbons), 68.9, 60.3, 56.8, 21.5, 21.2 (five aliphatic carbons). HRMS (ESI-TOF, m/z): 376.1333 (M+H), calculated, 376.1336.

**(2S)-1-(4-Methylbenzenesulfonyl)-*N***-**(4-methylpyridin-2-yl)pyrrolidine-2-carboxamide (7p)**

Appearance: yellowish oil; Yield (0.3509 g, 97.7%), FTIR (KBr, cm^-1^): 3401 (NH), 3001 (C-H aromatic) 2980 (C-H aliphatic), 1673 (C = O), 1640 (C = N), 1598, 1494, 1451, 1402 (C = C), 1338, 1305 (2S = O), 1202, 1156 (SO_2_N), 1093, 1015 (C-N). ^1^H NMR (DMSO-d_6_, 400 MHz) δ: 7.71–7.67 (m, 3H, ArH), 7.39–7.37 (m, 2H, ArH), 6.33 (d, J = 5.15 Hz, 2H, ArH), 6.28 (s, 1H, ArH), 6.06 (s, 1H, NH), 4.44 (dd, J = 5.15, 4.00 Hz, 1H, CH-C = O), 4.20 (dd, J = 4.00, 4.00 Hz, 1H, CH_a_ of CH_2_N), 4.03–4.01 (m, 1H, CH_b_ of CH_2_N), 3.54–3.50 (m, 1H, CH), 2.36–2.32 (m, 3H, CH_3_-Ar), 2.11 (s, 3H, CH_3_-Py), 1.92–1.72 (m, 2H, CH_2_), 1.52–1.49 (m, 1H, CH). ^13^C NMR (DMSO-d_6_, 400 MHz) δ: 173.9, 159.6 (C = O), 148.9, 146.1, 135.7, 130.4, 130.3, 129.4, 128.7, 127.7, 113.9, 109.1 (nine aromatic carbons), 61.2, 59.5, 48.9, 30.9, 21.5, 21.2 (six aliphatic carbons). HRMS (m/z): 359.1057 (M^+^), calculated, 359.1059.

#### Ethics statement on animal use

The animal use and care was approved by University of Nigeria, Nsukka animal ethics committee in confirmation to international standards for the project number PG/PhD/14/69629. The animal house of the Department of Biochemistry was used for the experiment. The project was designed to use humane endpoints for death of animals. During the entire experimental session, the mice were well nursed to reduce all kinds of stress. All efforts were made to minimize the suffering of the mice by strictly adhering with the regulation of animal protection committee.

#### *In vitro* anti-inflammatory activities determination

Male albino rats weighing 300 g where purchased from the Department of Zoology and Environmental Biology, University of Nigeria, Nsukka and kept at room temperature in a light controlled animal house. They were fasted with free access to water at least 12 h prior to the experiments. The tested compounds were prepared as suspension in vehicle (0.5% methylcellulose) and celecoxib was used as a standard drug. The positive control received celecoxib while the negative control received only the vehicle. Edema was produced by injecting 0.2 mL of a solution of 1% carrageenan in the hind paw. The rats were injected intraperitoneally with 1 mL suspension in 0.5% methylcellulose of the tested compounds and reference drug. Paw volume was measured by water displacement with a plethysmometer (UGO BASILE) before, 30 min, 1 h, 2 h and 3 h after treatment. The percentage was calculated by the following equation [25].

Anti-inflammatory activity (%) = (1-D/C)-100, where D represents the difference in paw volume before and after drug administration to the rats and C represents the difference of volume in the control groups. The approval for the use of animal was obtained from the University of Nigeria committee on experimental animal use. No sign of death was observed during the experiment.

#### Determination of LD_50_ for the active anti-inflammatory compounds

Male mice were divided into various groups and test compounds were administered in various doses intraperitoneally. Following treatments, the animals were observed for up to 6 h continuously and were then kept under observation for 72 h. All behavioral changes and deaths during the observation periods were recorded. The percentage of death at each dose level was then calculated, converted to probits and the LD_50_ (μM/kg) values were calculated [26]. To observe the health status of the mice, they were monitored 4 times a day. Humane endpoints were used when the animal shows sign of weight loss, weakness accompanied by inability to get food, complete anorexia and convulsion for 24 h. For the purpose of ameliorating the suffering of the dying mice, CO_2_ euthanasia was applied. All the dead mice were disposed in bio-safety containers in accordance with local standard protocols. The mortality rate in each group was calculated according to the formula:

Mortality rate(%)=(the number of dead mice/the number of mice in the group)×100.

#### *In vitro* cyclooxygenase inhibitory assay

The *in vitro* ability of test compounds and celecoxib to inhibit the COX-1 and COX-2 isozymes was carried out using Cayman colorimetric COX (ovine) inhibitor screening assay kit supplied by Cayman chemicals, USA. The calculations were performed as per the kit guidelines [27].

## Results and discussion

### Chemistry

Considering the ubiquitous pharmacological activities of sulphonamides and carboxamides as found in the literature and the need for anti-inflammatory agents with reduced side effects, we undertook the design and synthesis of some novel hybrids which possess advantages of the three pharmacophores of sulphonamide, carboxamide and 2-picoline in single molecular backbone. Our design strategy employed the use of boric acid in the condensation of 2-picoline with substituted benzenesulphonamides derived from L-amino acids. The use of boric acid was aimed to achieve the successful formation of carboxamide from un-activated carboxylic acid end of the L-amino acid in the presence of sulphonamide functionality. During the course of this study, we synthesized and characterized hybrid compounds containing sulphonamide, carboxamide and 2-picoline motifs. We took into consideration the problems of oral bioavailability and transportation of drug molecules in the design and as such the pharmacokinetics prediction of the designed molecules were evaluated prior to synthesis to eliminate compounds with associated oral bioavailability and transport problems.

**Fig 1:**
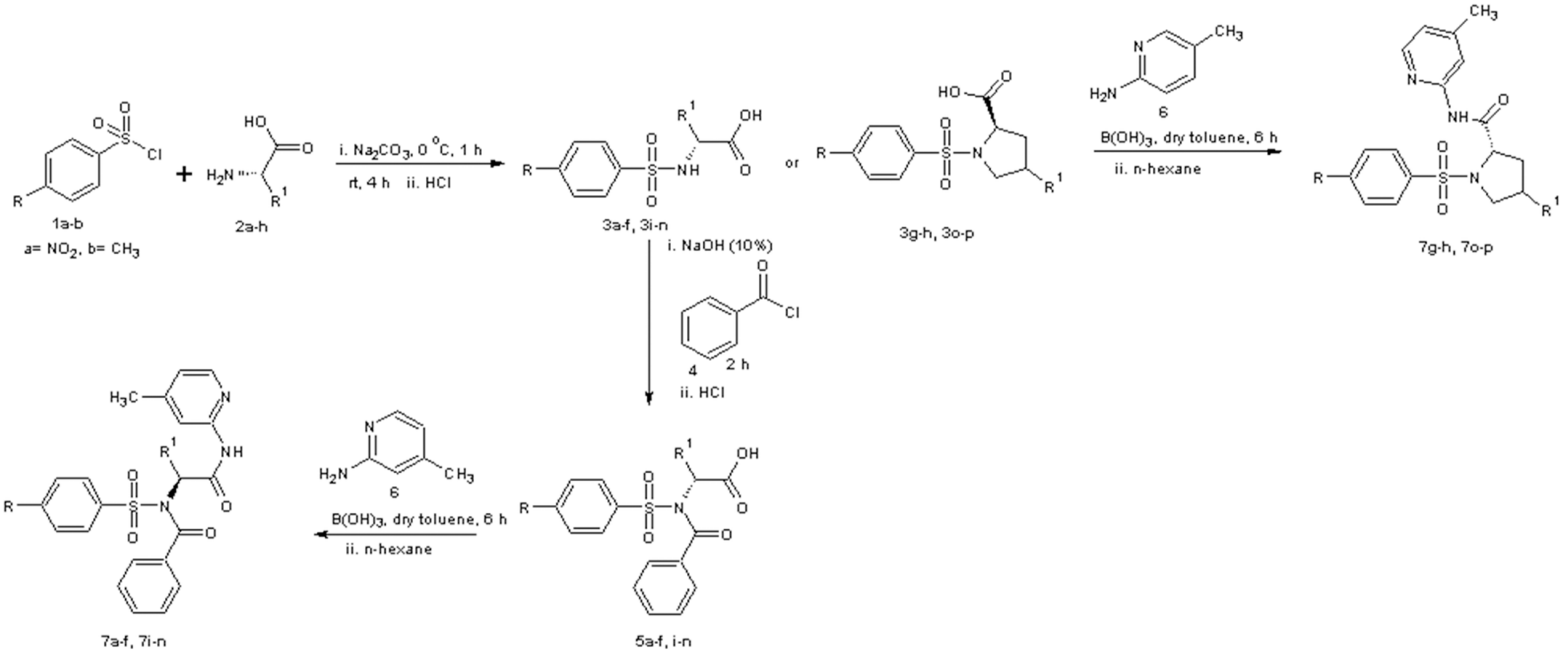
Synthesis protocol. A representation of the stepwise reactions leading to the desired compounds 7a-p.

**Fig 2:**
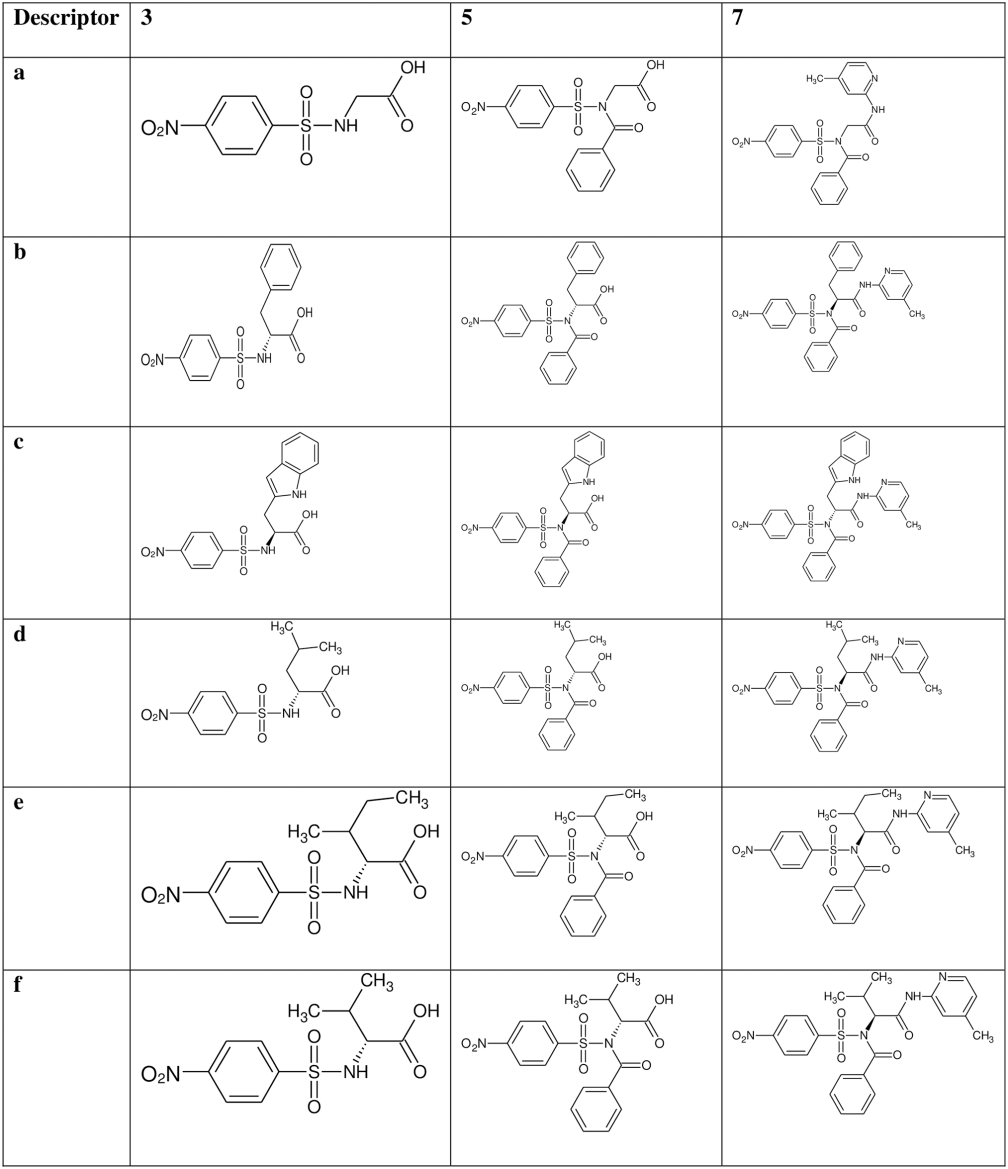
Description of *p*-nitrobenzenesulphonamide derivatives 7a-f. A structural representation of the *p*-nitrobenzenesulphonamide derivatives 7a-f and the intermediates 3a-f and 5a-f.

**Fig 3:**
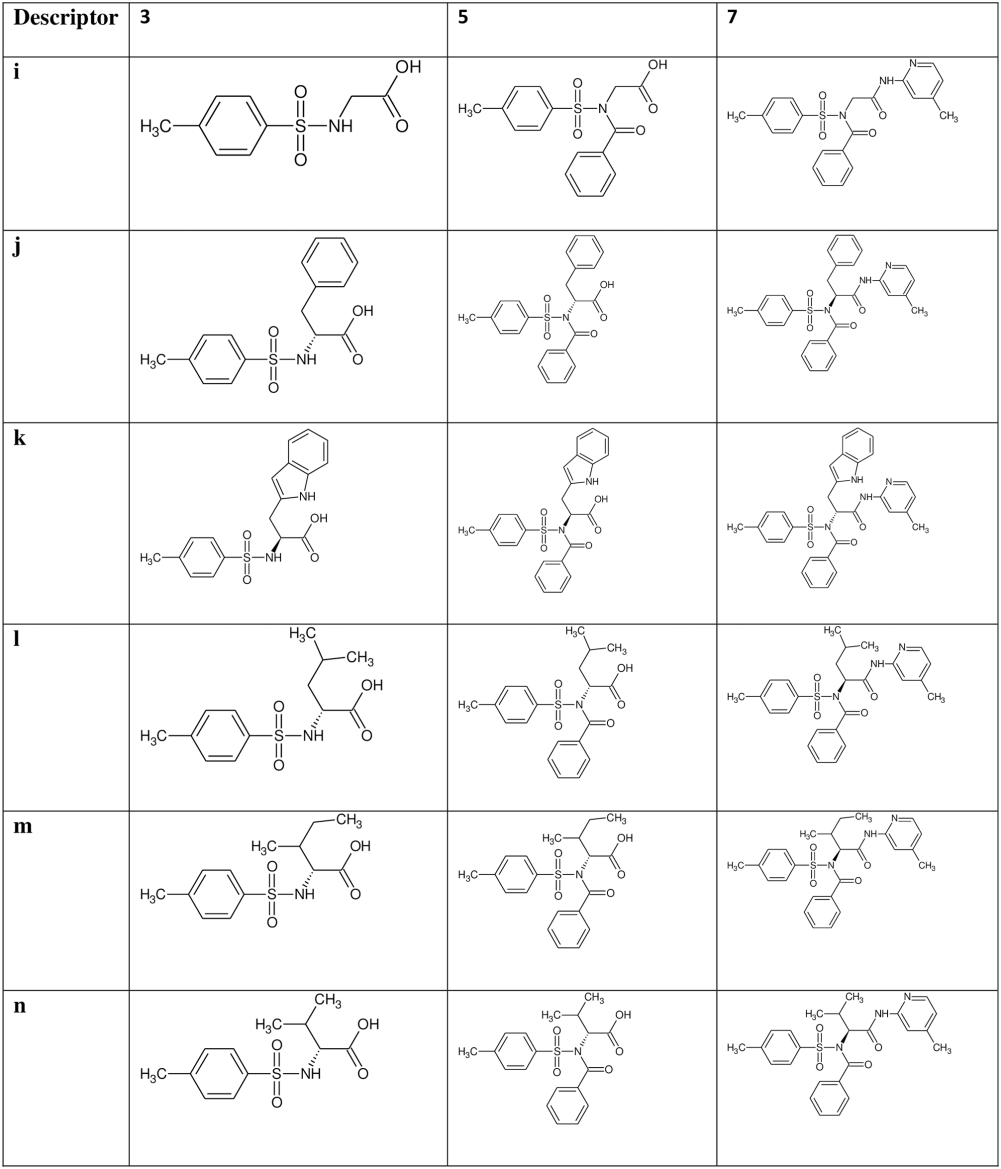
Description of *p*-toluenesulphonamide derivatives 7i-n. A structural representation of the *p*-toluenesulphonamide derivatives 7i-n and the intermediates 3i-n and 5i-n.

**Fig 4:**
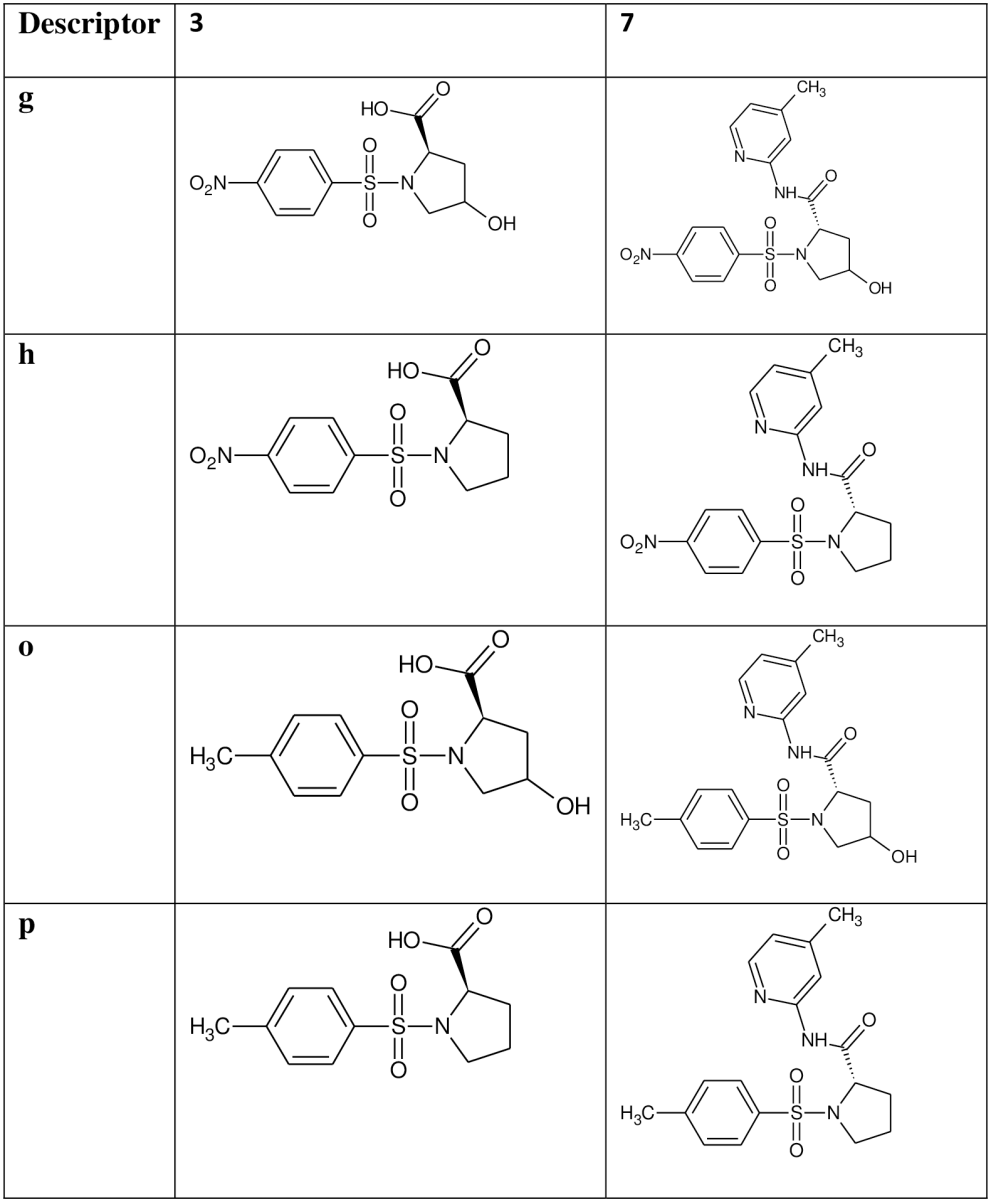
Description of L-proline derived sulphonamides 7g-h and 7o-p. A structural representation of the L-proline derivatives 7g, 7h, 7o and 7p and the intermediates 3g-h and 3o-p.

The reaction of substituted benzenesulphonyl chloride (**1a-b**) with various amino acids gave the substituted benzenesulphonamoyl alkanamides (**3a-p**). The base promoted reaction of **3a-f** and **3i-n** with benzoyl chloride under nitrogen at room temperature afforded the *N*-benzoylated derivatives (**5a-f** and **5i-n**). Further reaction of compounds **3g-h, 3o-p, 5a-f** and **5i-n** with 2-amino-4-picoline in the presence of catalytic amount of boric acid gave the new carboxamides **7a-p** in excellent yield (Figs 1–4).

In the FTIR of the substituted benzenesulphonamoyl alkanamides (**3a-p**), the bands between 3297–3253 cm^-1^ was due to NH group; the band between 1753–1705 cm^-1^ were assigned to the C = O of the carboxylic acid. In compounds **3a-h**, the N-O of NO_2_ appeared between 1532–1403 which were absent in the *p*-toluene derivatives **3i-p**. In the ^1^H NMR, the diagnostic NH peak appeared as a triplet in compounds **3a** and **3i** around 8.44–8.41 ppm; a doublet in compounds **3b-3f** and **3j-3n** around 8.69–8.42 ppm and disappeared in compounds **3g-h** and **3o-p**. In the ^13^C NMR, the diagnostic carbonyl appeared between 173.4–170.7 ppm. Additionally, all the carbons were accounted for.

In the benzoyl derivatives (**5a-f** and **5i-n**), the FTIR showed additional C = O band between 1691–1685 cm^-1^. The disappearance of the NH bands in the benzoyl derivatives further indicate successful coupling of the benzoyl chloride with substituted benzenesulphonamoyl alkanamides. In the ^1^H NMR, the disappearance of the NH peak is diagnostic. In addition, the benzoyl protons were accounted for in the aromatic region.

The FTIR spectra of the *p*-nitro derivatives **7a-h** (Fig 2) showed diagnostic bands at 3422–3265, 1773–1670, 1671–1661, 1641–1618 and 1535–1521 cm^-1^ corresponding to NH of carboxamide, C = O of carboxamide, C = O of benzoyl group, C = N of pyridine and NO_2_ of *p*-nitrobenzene respectively. The ^1^H NMR showed peaks between 7.75–7.73 and 2.17–2.16 ppm are diagnostic of pyridine ring and methyl of pyridine respectively. The ^13^C NMR of the compounds at 159.7–157.6, 152.0–148.9 and 21.5–21.2 ppm corresponds to C = N, C-N and methyl carbon of pyridine.

The *p*-toluenebenzenesulphonamide derivatives (**7i-p**, Fig 3) showed similar pattern of absorption in the FTIR, ^1^H NMR, ^13^C NMR and HRMS as recorded in compounds **7a-h** worthy of mention in the FTIR is the absorptions between 3401–3196 cm^-1^ due to NH stretch, 1692–1673 cm^-1^ due to C = O stretch and 1641–1612 cm^-1^ due to **C = N** of picoline. We observed a reduction in the bands of the C = O in the derivatives which is attributable to successful coupling. These bands are indicative of successful coupling of the 4-aminopicoline with the carboxylic end of the 4-methylbenzenesulphonamides. In the ^1^H NMR (S1–S57 Figs), the peaks between δ7.71–7.69 (d, 1H); δ6.35–6.32 (d, 1H) and δ6.82–6.27 (s, 1H) were assigned to the three hydrogen of picoline in all the derivatives. In the ^13^C NMR spectra (S1–S57 Figs) of the 4-methylbenzenesulphonamide derivatives **7i-p**, the appearance of the azomethine peak between 159.6–158.8 ppm indicated the successful formation of the target molecules. The spectral analysis above in addition to the molecular ion peaks showed that there was successful coupling of the amino group of 4-aminopicooline with the carboxylic acid of the 4-methylbenzenesulphonamoyl ethanamides.

**Fig 5:**
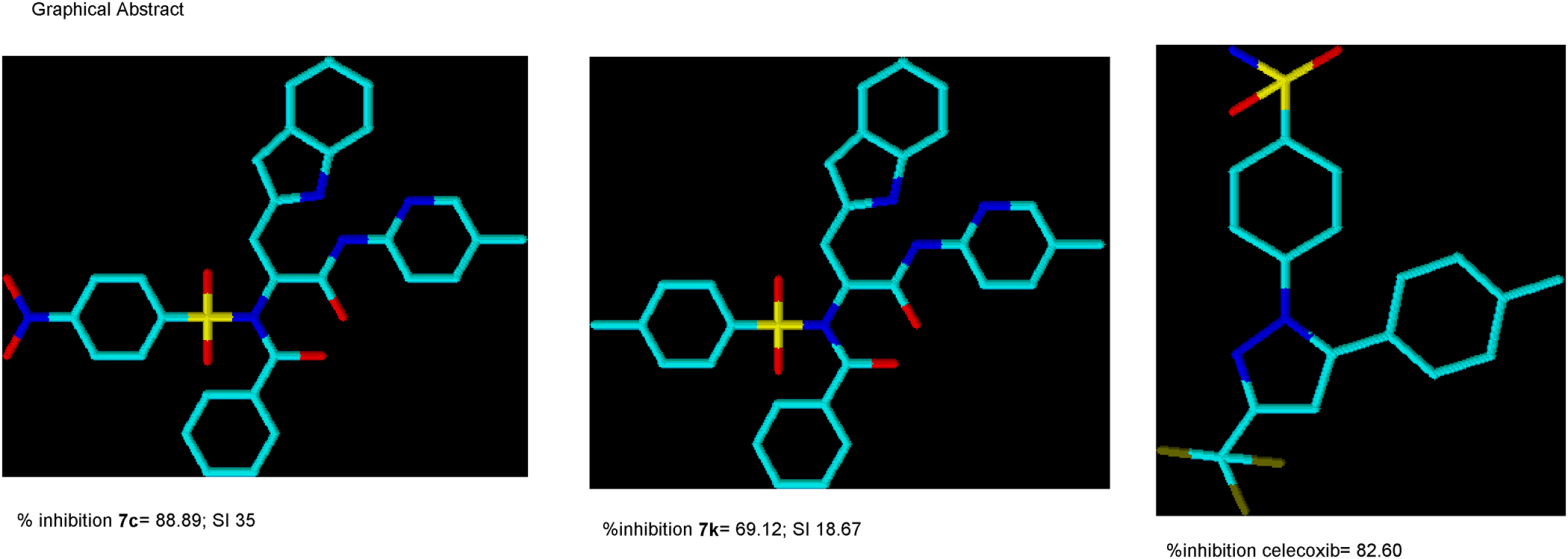
Graphical abstract.

The yield suggests that compounds **7i-p** had better yield than compounds **7a-h**, this suggests that the presence of an electron donating group at the para position in the benzenesulphonamoyl group favours the reaction more than an electron withdrawing nitro group. Nonetheless, all the derivatives had excellent yields ranging from 97.5–100 in the para nitro derivatives and 97.6–100 in the para methyl derivatives. The 3D structures of the most active derivatives **7c** and **7k** in comparison with celecoxib are shown in the graphical abstract (Fig 5).

### Biological evaluations

#### Anti-inflammatory activity

The anti-inflammatory activity was performed to get percentage inhibition of carrageenan induced rat paw edema for each of the tested compound after 0.5, 1, 2 and 3 h and to compare it with reference drug celecoxib, however, the significant decrease in the activity of all compounds except **7c, 7g, 7k** and celecoxib after 2 h was noticed (Table 1). Only compounds **7c, 7g** and **7k** (% inhibition = 88.89, 69.12 and 61.58% respectively) showed anti-inflammatory activities comparable with celecoxib (% inhibition = 82.60%) [28]. The L-tryptophan and L-4hydroxyproline derivatives were the most active derivatives. This result suggests that derivatives with having atoms capable of forming hydrogen bond had better anti-inflammatory activity. The substitution of the methyl group in *p*-toluenesulphonyl derivative **7k** with a nitro group (atom capable of hydrogen bonding) led to a better anti-inflammatory activity. The presence of nitro group at the para position seem to increase the anti-inflammatory activities as shown in Table 1; compounds **7a-h** having the para nitro group showed better anti-inflammatory activities than the corresponding methyl derivatives (**7i-p**). In addition, LD_50_ of compound **7c, 7g** and **7k** (LD_50_ = 8.3, 9.9 and 9.2 μM/kg respectively) related to celecoxib (LD_50_ = 9.8 μM/kg).

**Table 1:** *In vitro* anti-inflammatory activity.

S/N	0.5 h (%)	1 h (%)	2 h (%)	3 h (%)	LD_50_ (μM/kg)	COX-1 (%)	COX-2 (%)	S I
**7a**	24.62	18.82	14.24	11.85	12.9	14	49	3.5
**7b**	32.31	17.06	17.03	12.96	14.2	8	52	6.5
**7c**	49.23	62.35	75.75	88.89	8.3	2	70	35
**7d**	52.31	17.65	10.54	10.15	12.6	10	20	2.00
**7e**	26.15	25.88	19.38	16.05	13.1	4	17	4.25
**7f**	24.62	17.65	14.84	1.85	15.8	15	54	3.6
**7g**	46.92	53.53	58.84	61.58	9.9	18	20	1.11
**7h**	27.69	17.06	16.59	16.05	10.7	30	13	0.43
**7i**	24.62	23.53	16.78	12.47	18.2	3	18	6.00
**7j**	41.54	40.59	39.84	36.42	17.6	10	30	3.00
**7k**	42.12	56.12	70.89	69.12	9.2	3	56	18.67
**7l**	26.15	19.88	18.28	16.67	12.5	20	19	0.95
**7m**	13.85	12.35	8.59	8.58	11.9	17	25	1.47
**7n**	21.54	16.47	10.56	3.09	20.1	32	48	1.50
**7o**	21.54	11.23	8.78	5.56	10.1	6	18	3.00
**7p**	29.15	28.24	30.34	33.26	9.9	8	20	2.5
**Celecoxib**	56.92	63.53	64.84	82.60	9.8	2	61	30.5

#### *In vitro* COX inhibitory activity

Percentage inhibition of COX-1 and COX-2 and selectivity (COX-2/COX-1) of test compounds at concentration of 2.0 μM were illustrated in Table 1. Compounds **7g, 7h, 7l, 7m** and **7n** are non-selective for COX-2 inhibitors whereas compound **7c** showed better selectivity towards COX-2. Its COX-2/COX-1 selectivity is 35. Compound **7k** showed good selectivity towards COX-2. Its COX-2/COX-1 selectivity is 18.67, more than half of selectivity of celecoxib (COX-2/COX-1 = 30.5). This results indicate that the mechanism of action of test compounds could be COX-2 inhibition but we discovered that compound **7g** and **7k** did not show comparable selectivity in-spite of their comparable anti-inflammatory activity with celecoxib. The implication therefore is that the mechanism of test compounds needs further investigations.

#### Assessment of oral bioavailability property

Lipinski’s rule of five (ro5) alongside topological polar surface area (TPSA) properties were used to assess the oral bioavailability potential of the newly synthesized 4-picoline derivatives. Topological polar surface area is frequently used in drug design as surrogate property for cell permeability with a rule of thumb that a molecule with TPSA of less than 140 Å^2^ would be able to permeate the cell. It has also been used as a surrogate for penetrating the blood-brain-barrier (BBB) if the TPSA is ≤ 90 Å^2^. The results showed that all the new compounds can permeate cells but only compounds **7i-p** can permeate blood-brain-barriers and hence can be used in treating brain inflammation.

Lipinski’s ro5, derived from 90^th^ percentile of drug candidates that reach phase II clinical trials, to be drug-like, a drug candidate should have lipophilicity (logP) ≤ 5, number of hydrogen bond acceptor (HBA) ≤ 10, molecular weight (MW) ≤ 500 and number of hydrogen bond donor (HBD) ≤ 5. This rule claims that violation of more than one property will disqualify a drug candidate based on associated bioavailability problem. The results (Table 2) showed that all the synthesized compounds are drug-like considering ro5 since none of the compounds violated more than one property. Verber *et al* [29] reported that the number of rotatable bond (nRB) influences bioavailability in rats and recommended NRB ≤ 10 for good oral bioavailability property. Compounds **7b-7e** were disqualified because they had NRB of 11 which was greater than the recommended value. Other derivatives reported had good oral bioavailability with respect to number of rotatable bond. Additionally, the number of acid was zero which was a good requirement for blood-brain-barriers.

**Table 2:** Physicochemical properties for drug-likeness.

S/N	MW	logP	HBA	HBD	TPSA (Å^2^)	nRB	nAc	NV
**7a**	454	0.319	7	0	135.4	9	0	0
**7b**	544	2.094	7	0	135.4	11	0	1
**7c**	583	1.701	8	0	135.4	11	0	1
**7d**	509	2.28	7	0	135.4	11	0	1
**7e**	509	2.069	7	0	135.4	11	0	1
**7f**	496	1.50	7	0	135.4	10	0	0
**7g**	406	-0.95	7	0	118.33	6	0	0
**7h**	390	0.077	6	0	118.33	6	0	0
**7i**	423	0.705	7	0	92.26	8	0	0
**7j**	513	2.48	7	0	92.26	10	0	0
**7k**	552	2.087	8	0	92.26	10	0	0
**7l**	479	2.666	7	0	92.26	10	0	0
**7m**	479	2.455	7	0	92.26	10	0	0
**7n**	465	1.886	7	0	92.26	9	0	0
**7o**	375	0.463	6	0	75.19	5	0	0
**7p**	359	-0.57	7	0	75.19	5	0	0

MW = molecular weight; HBA = hydrogen bond acceptor; HBD = hydrogen bond donor; TPSA = topological polar surface area; nRB = number of rotatable bond; nAc = number of acid and NV = number of violations

## Conclusion

In conclusion, 3-(1*H*-indol-2-yl)-*N*-(4-methylpyridin-2-yl)-2-[*N*-(4-nitrobenzenesulfonyl)-1-phenyl formamido]propanamide, 3-(1*H*-indol-2-yl)-2-[*N*-(4-methylbenzenesulfonyl)-1-phenylformamido]-*N*-(4-methylpyridin-2-yl)propanamide and 4-Hydroxy-*N*-(4-methylpyridin-2-yl)-1-(4-nitrobenzene sulfonyl)pyrrolidine-2-carboxamide **7c, 7k** and **7g** were found to have excellent anti-inflammatory activity with percentage inhibition of carrageenan induced rat paw edema of 88.89, 69.12 and 61.58% respectively comparable with that of celecoxib (% inhibition = 82.60%) after 3 h of administration. Compound **7c** appeared COX-2/COX-1 selectivity higher than celecoxib while compound **7k** appeared COX-2/COX-1 selectivity a little higher than half of celecoxib. Compound **7g** however is non-selective for COX-2. These finding suggests further investigation to the mechanism of action of the test compounds. On a broad basis, compounds **7c, g** and **k** seemed to be a promising anti-inflammatory agents.

## Supporting information

S1 Fig C-13 NMR spectrum of 7a. (TIF)

S2 Fig ^1^H NMR spectrum of 7b. (TIF)

S3 Fig ^1^H NMR spectrum of 7b (expansion). (TIF)

S4 Fig C-13 NMR spectrum of 7b. (TIF)

S5 Fig ^13^C NMR spectrum of 7b (expansion). (TIF)

S6 Fig ^1^H NMR spectrum of 7c. (TIF)

S7 Fig ^1^H NMR spectrum of 7c (expansion). (TIF)

S8 Fig ^1^H NMR spectrum of 7c (expansion). (TIF)

S9 Fig ^13^C NMR spectrum of 7c. (TIF)

S10 Fig ^13^C NMR spectrum of 7c (expansion). (TIF)

S11 Fig ^1^H NMR spectrum of 7d. (TIF)

S12 Fig ^1^H NMR spectrum of 7d (expansion). (TIF)

S13 Fig ^1^H NMR spectrum of 7d (expansion). (TIF)

S14 Fig ^13^C NMR spectrum of 7d (expansion). (TIF)

S15 Fig ^1^H NMR spectrum of 7e. (TIF)

S16 Fig ^1^H NMR spectrum of 7e (expansion). (TIF)

S17 Fig ^13^C NMR spectrum of 7e. (TIF)

S18 Fig ^1^H NMR spectrum of 7f. (TIF)

S19 Fig ^1^H NMR spectrum of 7f (expansion). (TIF)

S20 Fig ^13^C NMR spectrum of 7f. (TIF)

S21 Fig ^1^H NMR spectrum of 7g. (TIF)

S22 Fig ^1^H NMR spectrum of 7g (expansion). (TIF)

S23 Fig ^13^C NMR spectrum of 7g. (TIF)

S24 Fig ^1^H NMR spectrum of 7h. (TIF)

S25 Fig ^1^H NMR spectrum of 7h (expansion). (TIF)

S26 Fig ^1^H NMR spectrum of 7h (expansion). (TIF)

S27 Fig ^13^C NMR spectrum of 7d. (TIF)

S28 Fig ^1^H NMR spectrum of 7i. (TIF)

S29 Fig ^1^H NMR spectrum of 7i (expansion). (TIF)

S30 Fig ^13^C NMR spectrum of 7i. (TIF)

S31 Fig ^1^H NMR spectrum of 7j. (TIF)

S32 Fig ^1^H NMR spectrum of 7j (expansion). (TIF)

S33 Fig ^1^H NMR spectrum of 7j (expansion). (TIF)

S34 Fig ^13^C NMR spectrum of 7j. (TIF)

S35 Fig ^1^H NMR spectrum of 7k. (TIF)

S36 Fig ^1^H NMR spectrum of 7k (expansion). (TIF)

S37 Fig ^1^H NMR spectrum of 7k (expansion). (TIF)

S38 Fig ^13^C NMR spectrum of 7k. (TIF)

S39 Fig ^13^C NMR spectrum of 7k. (TIF)

S40 Fig ^1^H NMR spectrum of 7l. (TIF)

S41 Fig ^1^H NMR spectrum of 7l (expansion). (TIF)

S42 Fig ^1^H NMR spectrum of 7l (expansion). (TIF)

S43 Fig ^13^C NMR spectrum of 7l. (TIF)

S44 Fig ^1^H NMR spectrum of 7m. (TIF)

S45 Fig ^1^H NMR spectrum of 7m (expansion). (TIF)

S46 Fig ^1^H NMR spectrum of 7m (expansion). (TIF)

S47 Fig ^13^C NMR spectrum of 7m. (TIF)

S48 Fig ^1^H NMR spectrum of 7n. (TIF)

S49 Fig ^1^H NMR spectrum of 7n. (TIF)

S50 Fig ^13^C NMR spectrum of 7n. (TIF)

S51 Fig ^1^H NMR spectrum of 7o. (TIF)

S52 Fig ^13^C NMR spectrum of 7o. (TIF)

S53 Fig ^1^H NMR spectrum of 7p. (TIF)

S54 Fig ^1^H NMR spectrum of 7p (expansion). (TIF)

S55 Fig ^1^H NMR spectrum of 7p (expansion). (TIF)

S56 Fig ^1^H NMR spectrum of 7p (expansion). (TIF)

S57 Fig ^13^C NMR spectrum of 7p. (TIF)
