# Supplementary figures and images for: New carboxamide derivatives bearing benzenesulphonamide as a selective COX-II inhibitor: Design, synthesis and structure-activity relationship
Source: PLoS One. 2017 Sep 18;12(9):e0183807. doi: 10.1371/journal.pone.0183807 (PMC5602572; doi:10.1371/journal.pone.0183807)

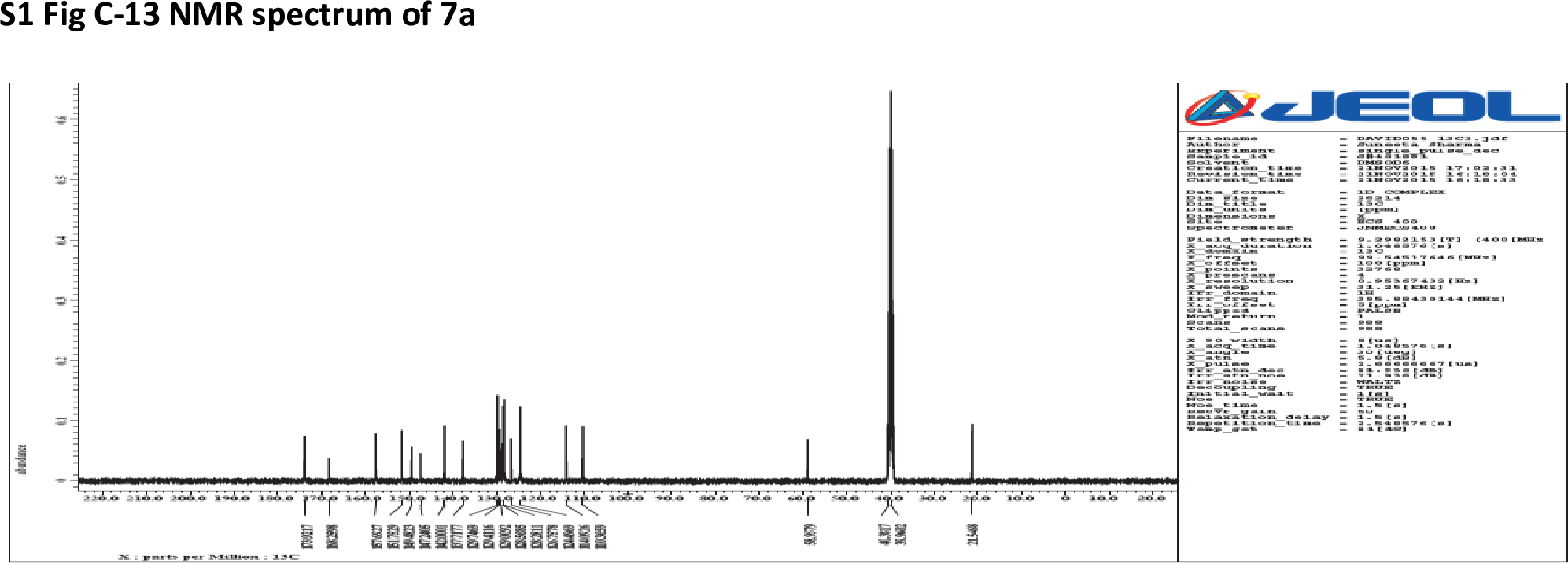

Supplement: S1 Fig — (TIF) [file pone.0183807.s001.tif]

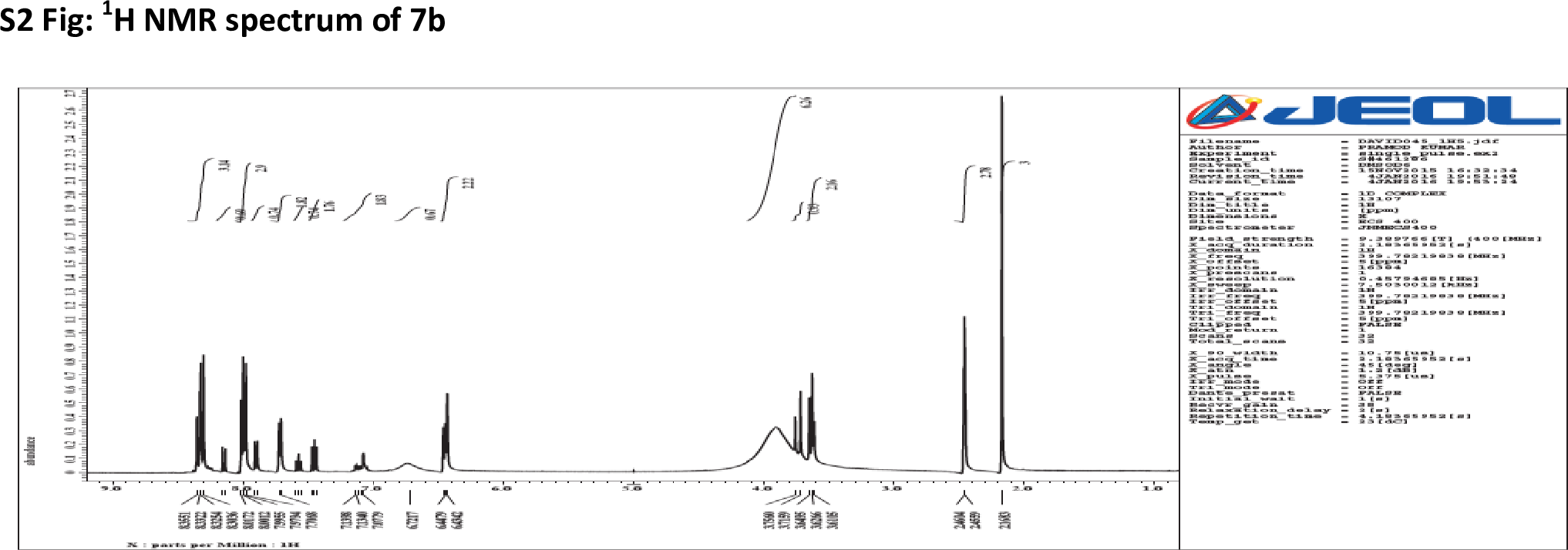

Supplement: S2 Fig — (TIF) [file pone.0183807.s002.tif]

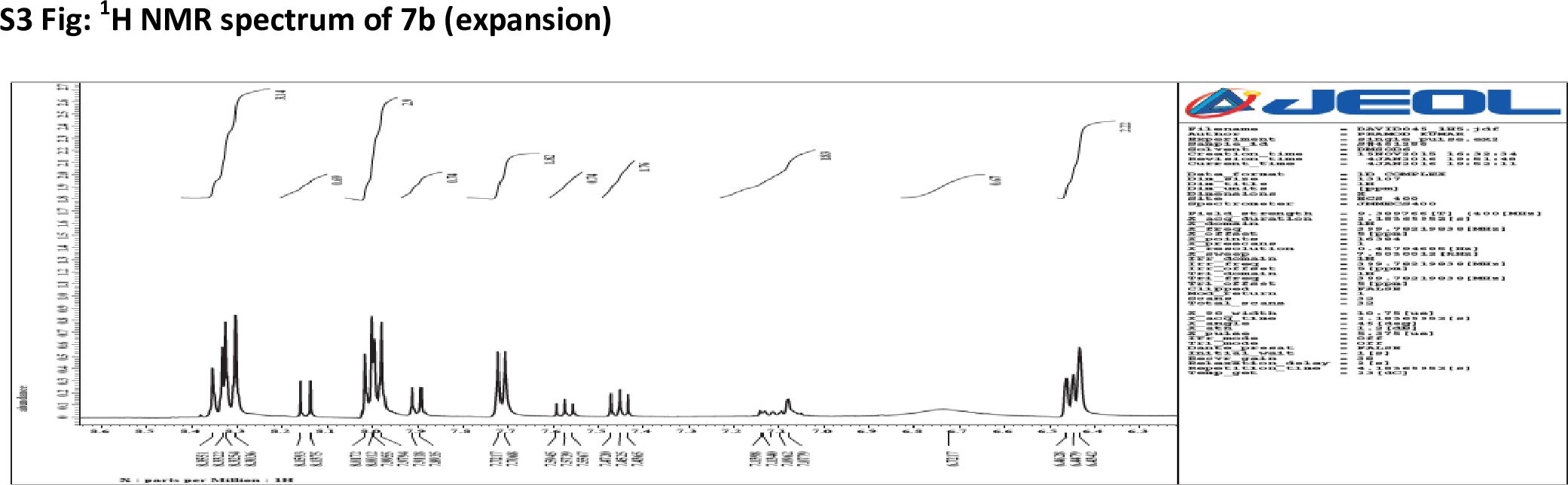

Supplement: S3 Fig — (TIF) [file pone.0183807.s003.tif]

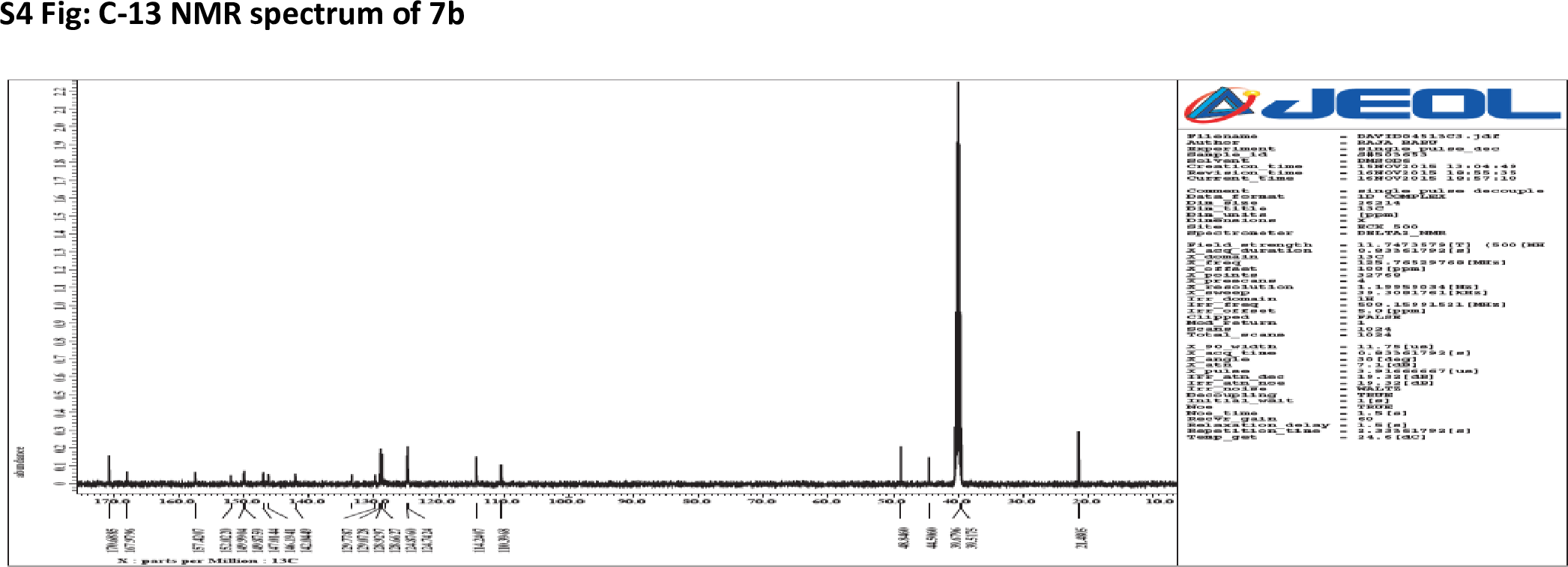

Supplement: S4 Fig — (TIF) [file pone.0183807.s004.tif]

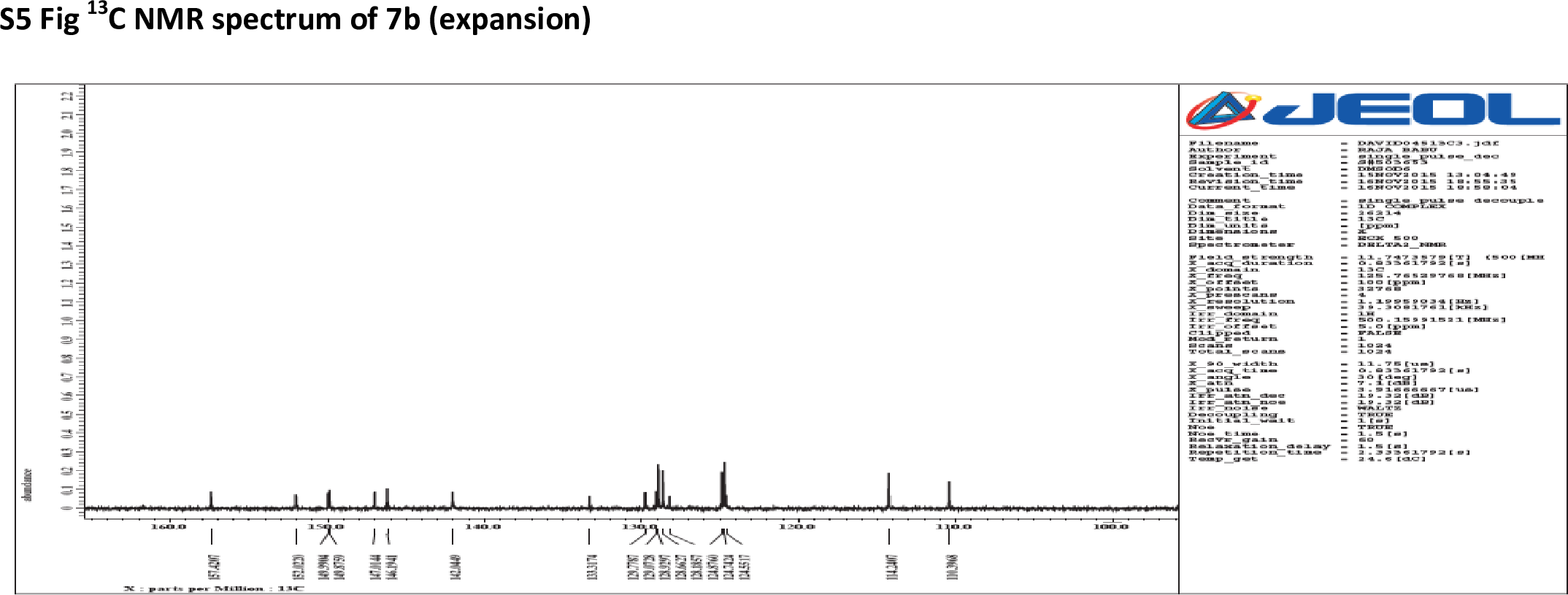

Supplement: S5 Fig — (TIF) [file pone.0183807.s005.tif]

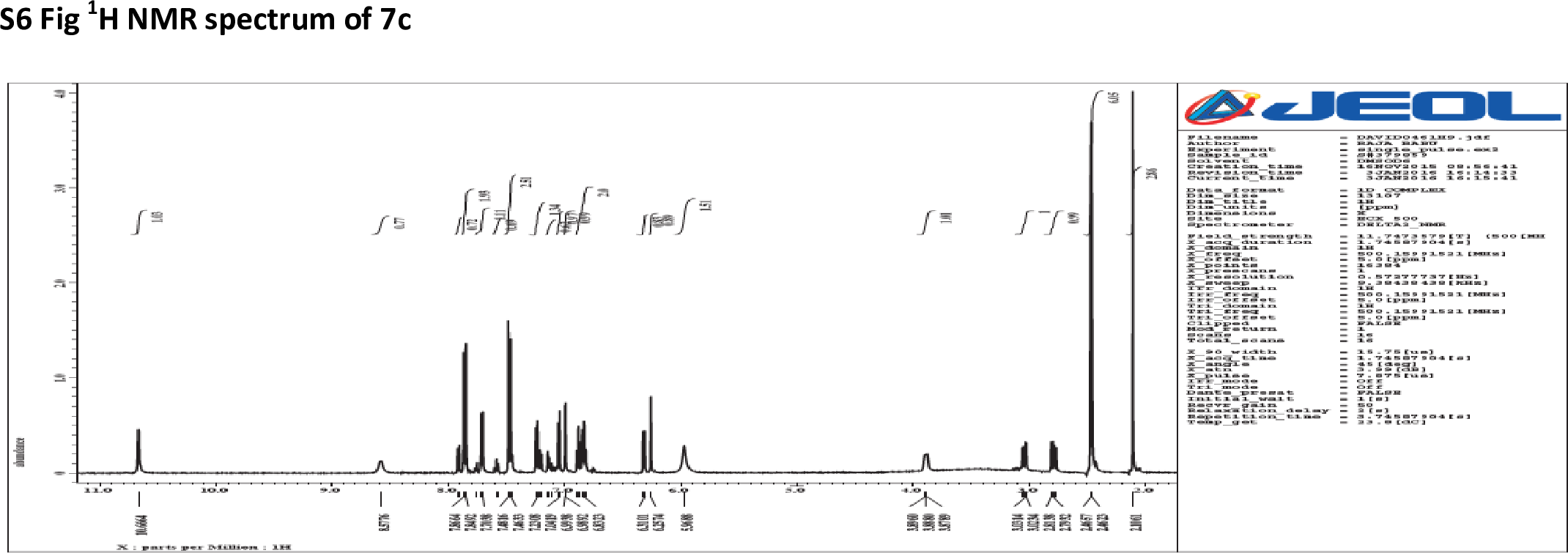

Supplement: S6 Fig — (TIF) [file pone.0183807.s006.tif]

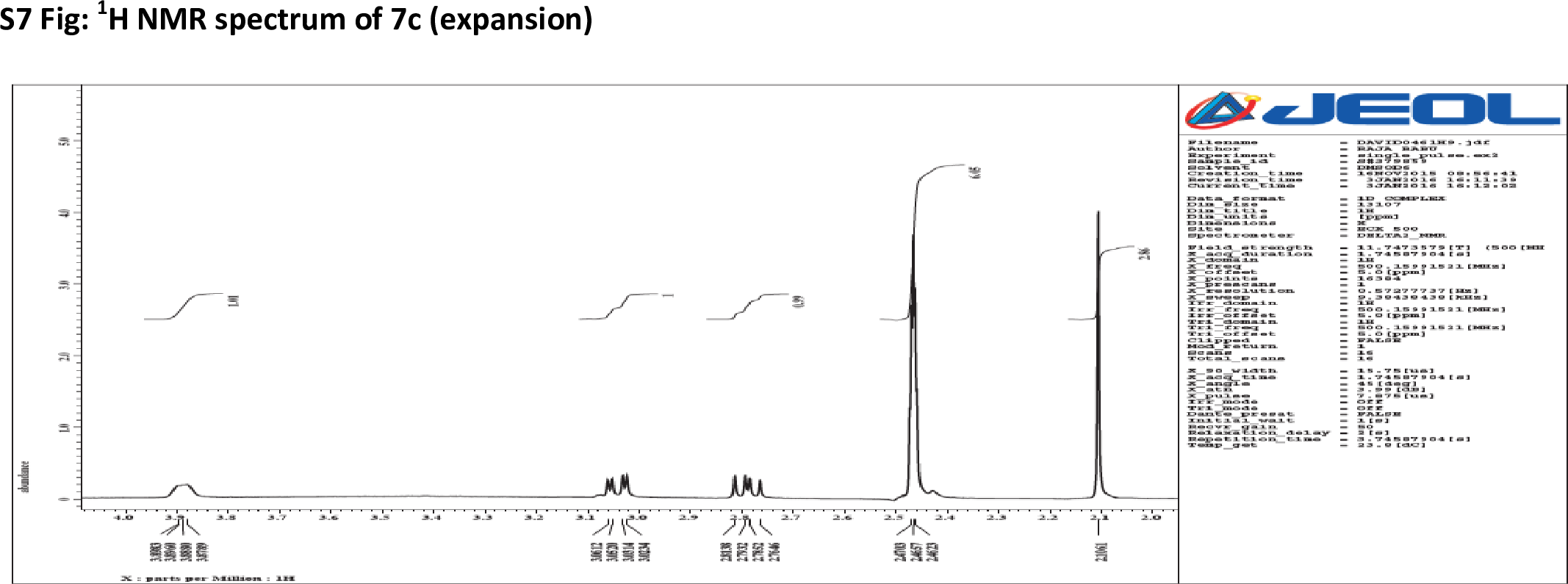

Supplement: S7 Fig — (TIF) [file pone.0183807.s007.tif]

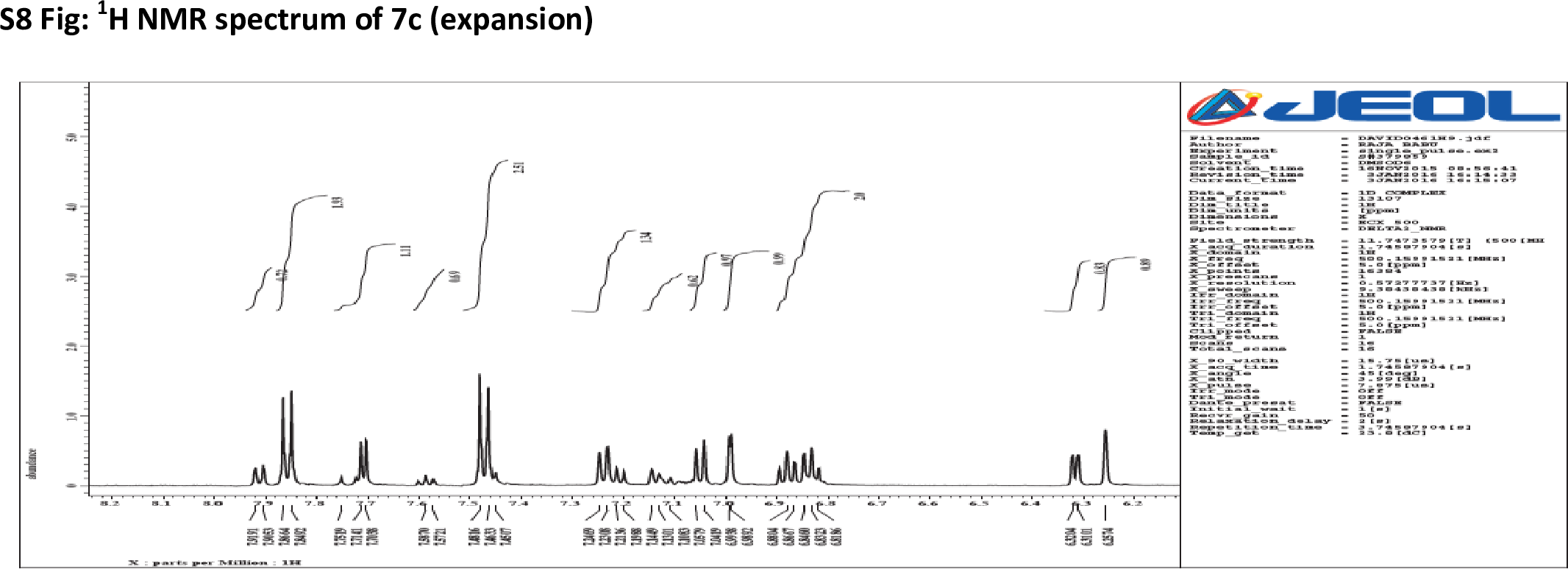

Supplement: S8 Fig — (TIF) [file pone.0183807.s008.tif]

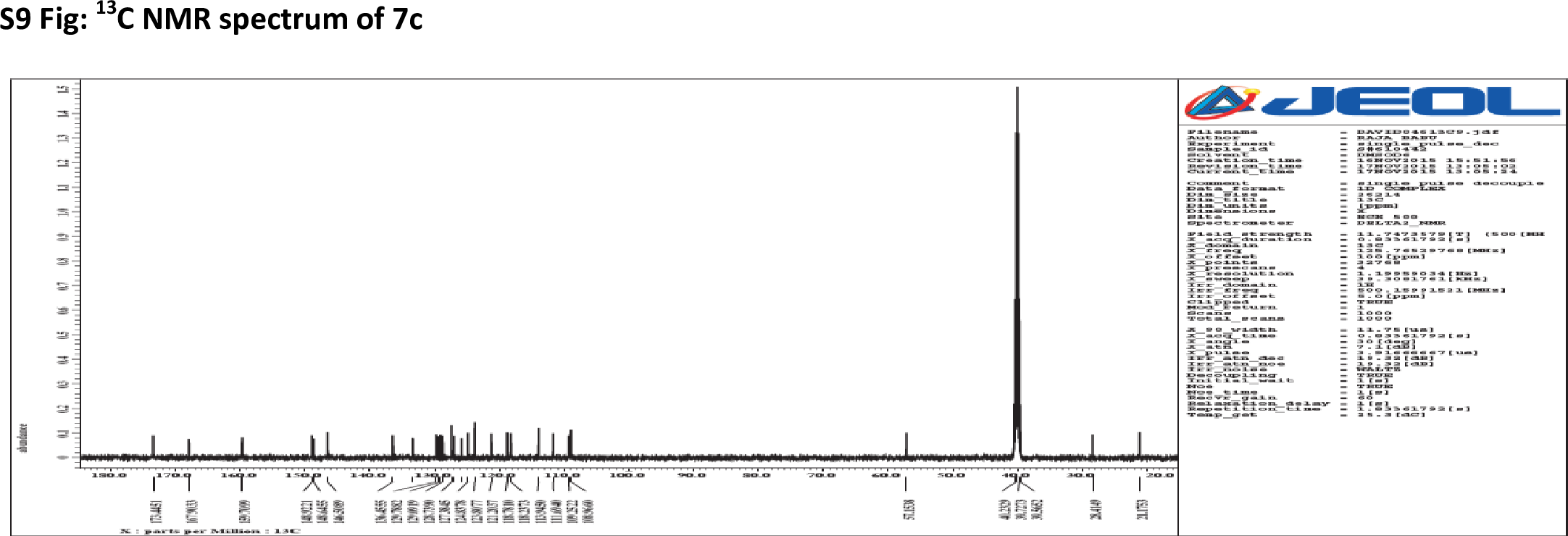

Supplement: S9 Fig — (TIF) [file pone.0183807.s009.tif]

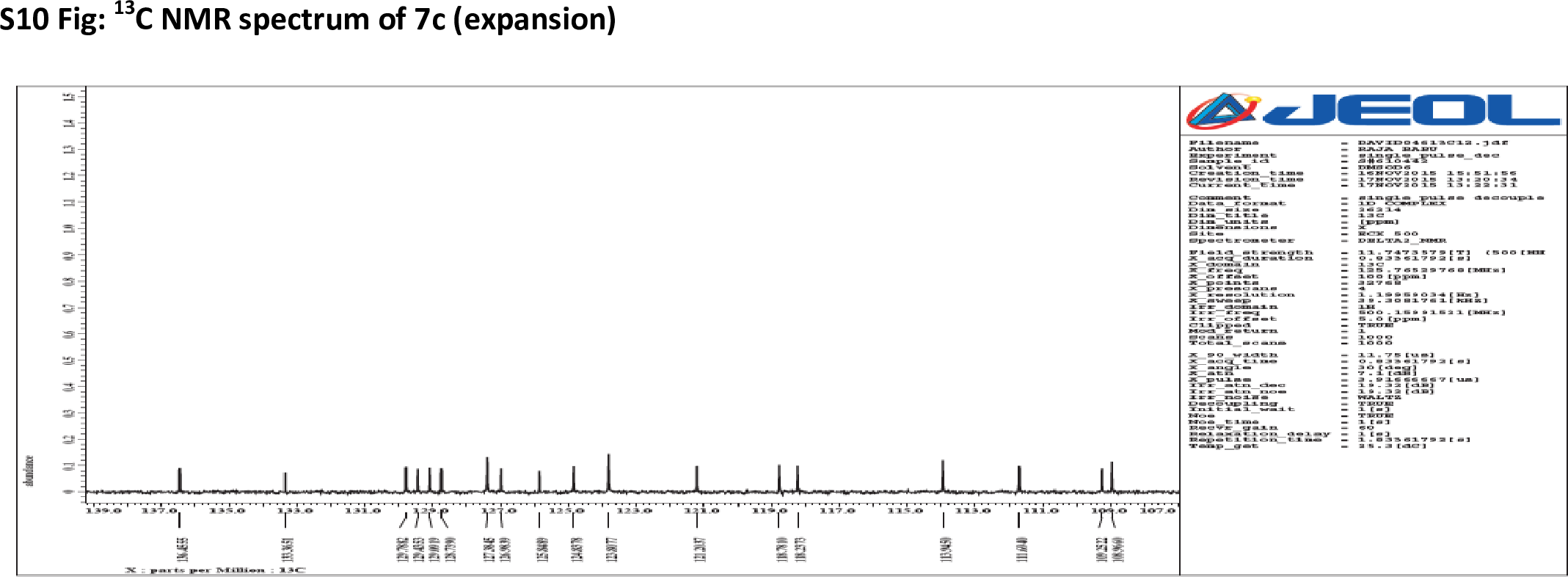

Supplement: S10 Fig — (TIF) [file pone.0183807.s010.tif]

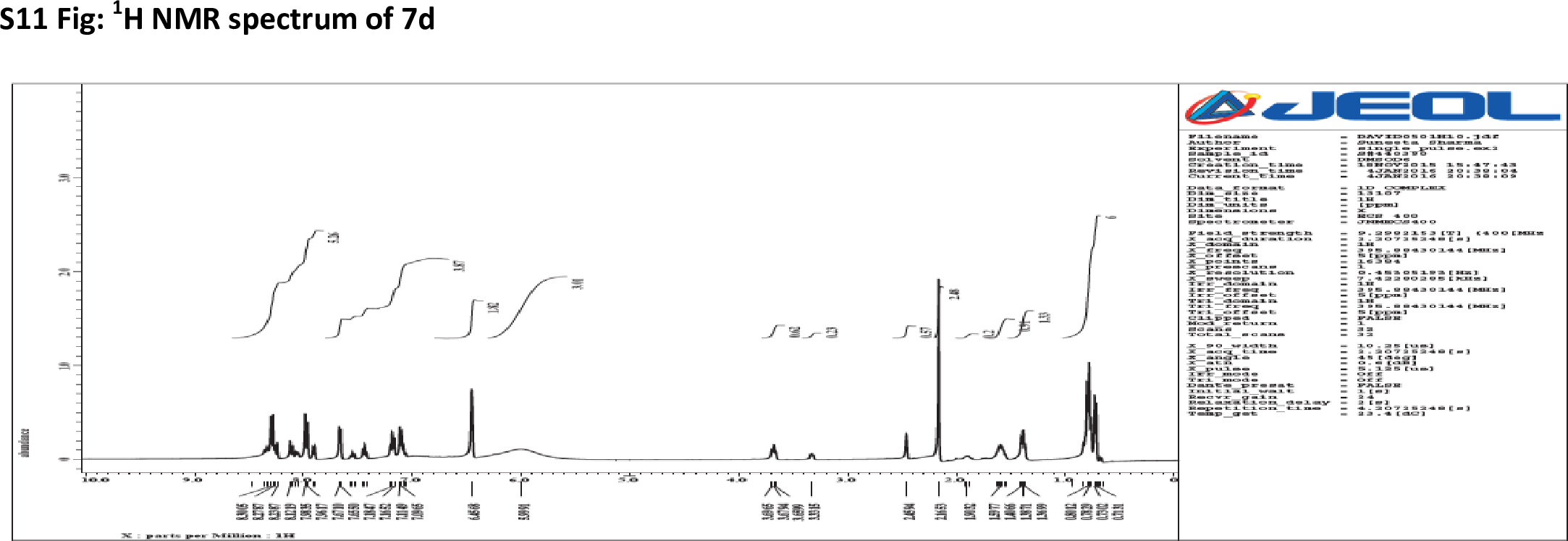

Supplement: S11 Fig — (TIF) [file pone.0183807.s011.tif]

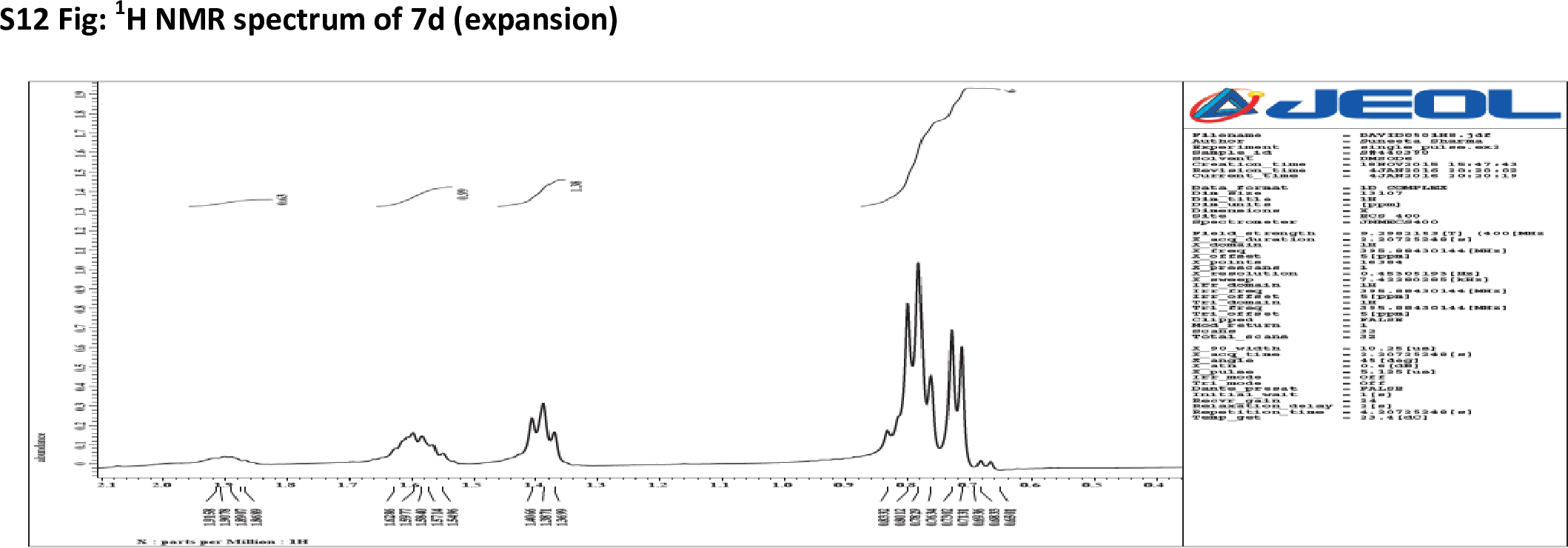

Supplement: S12 Fig — (TIF) [file pone.0183807.s012.tif]

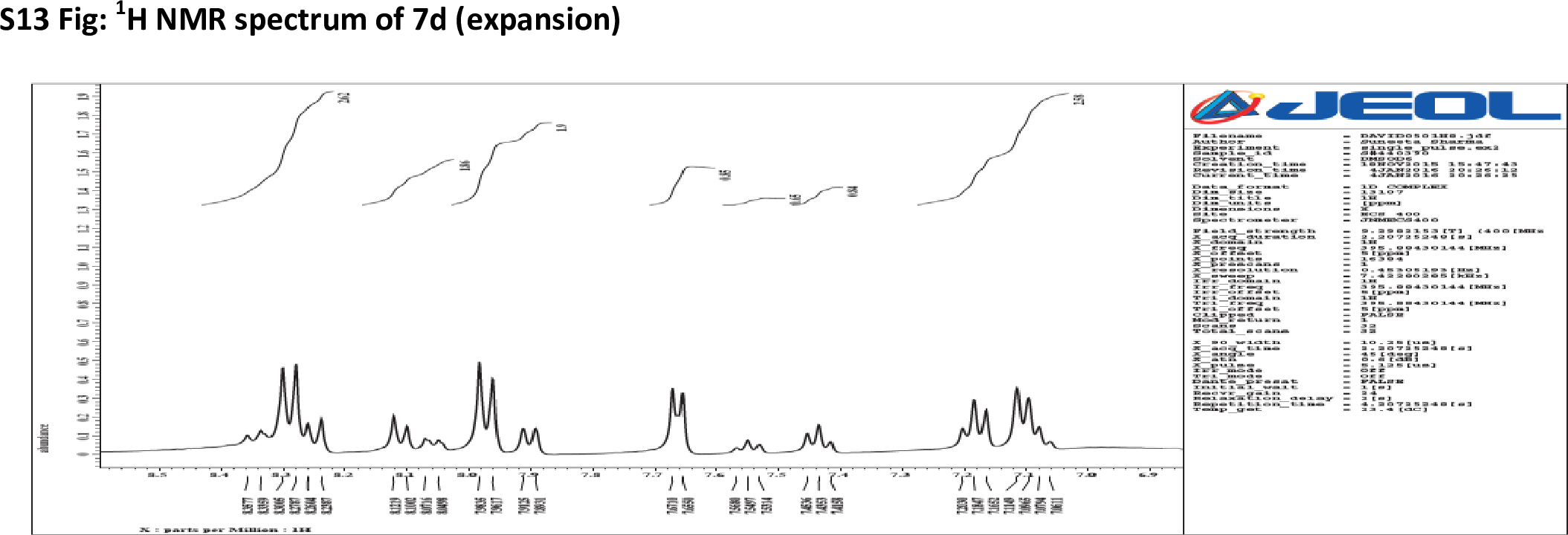

Supplement: S13 Fig — (TIF) [file pone.0183807.s013.tif]

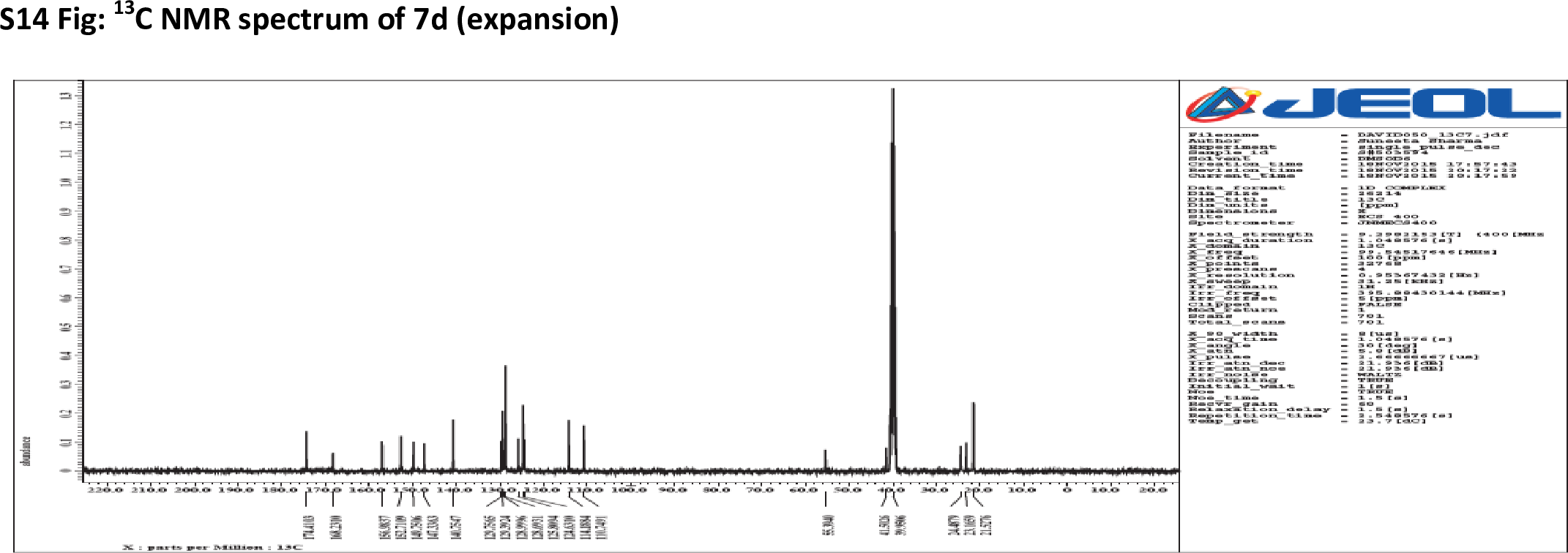

Supplement: S14 Fig — (TIF) [file pone.0183807.s014.tif]

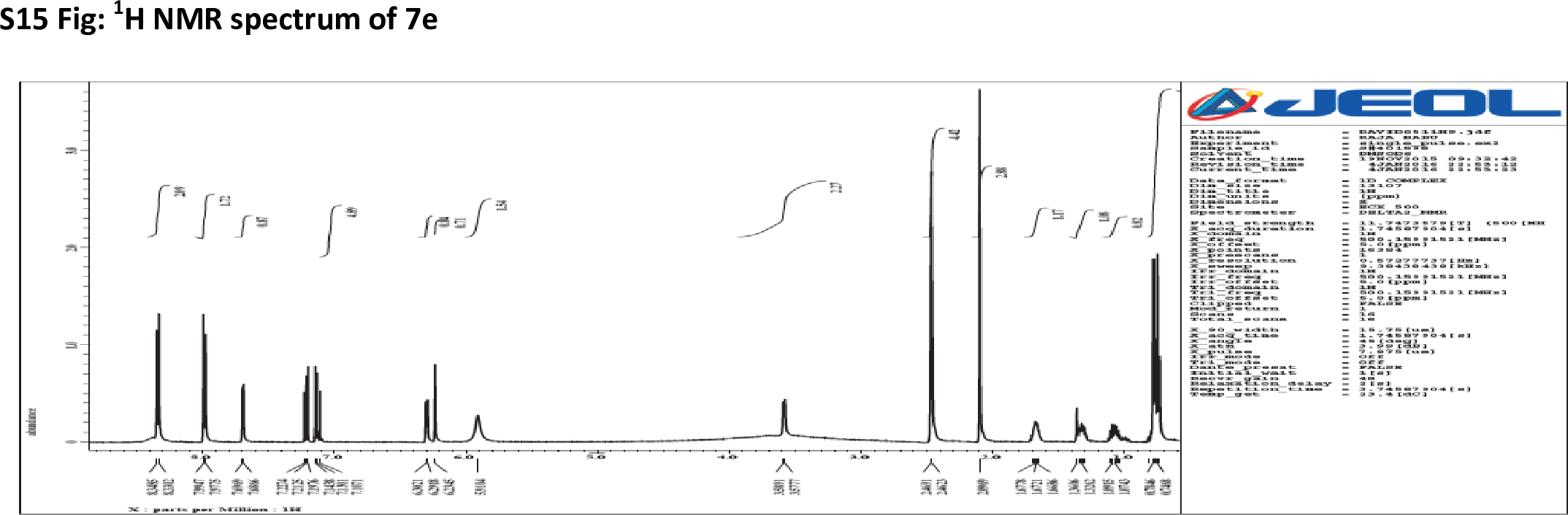

Supplement: S15 Fig — (TIF) [file pone.0183807.s015.tif]

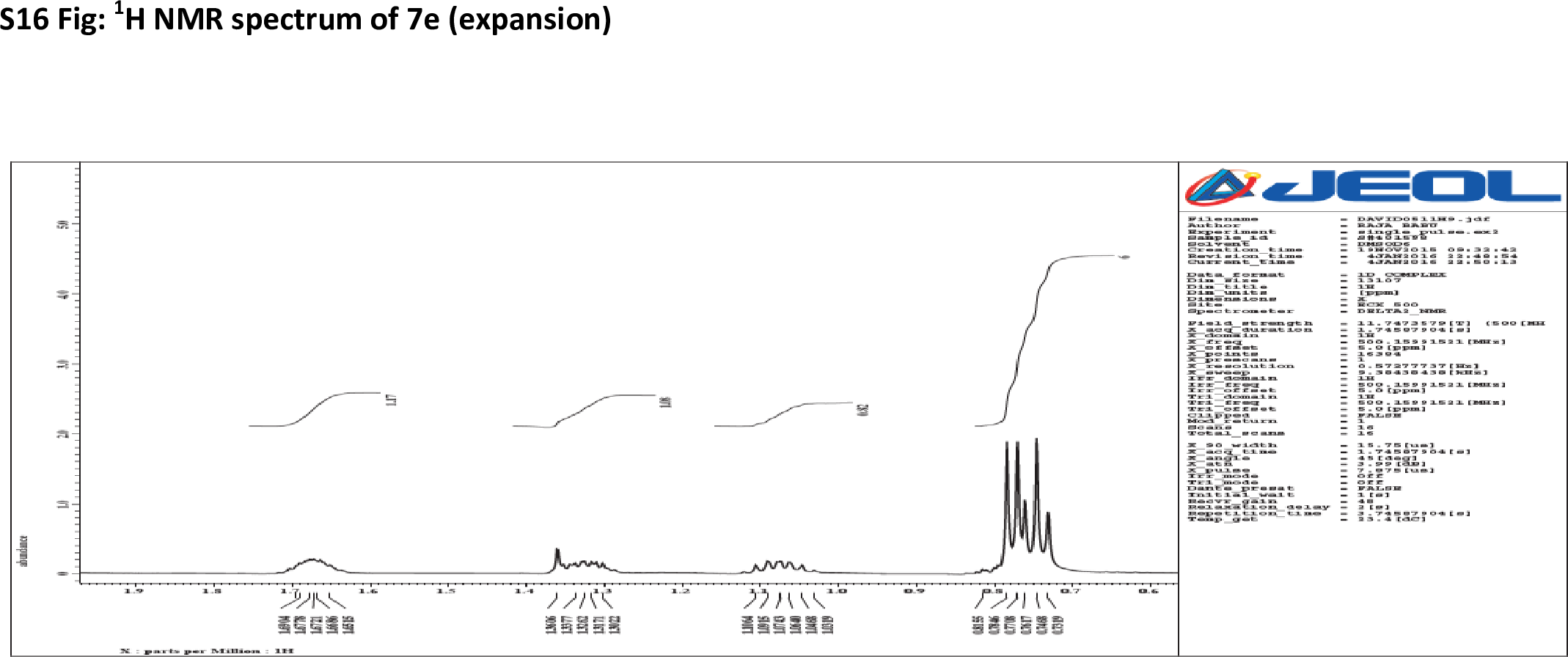

Supplement: S16 Fig — (TIF) [file pone.0183807.s016.tif]

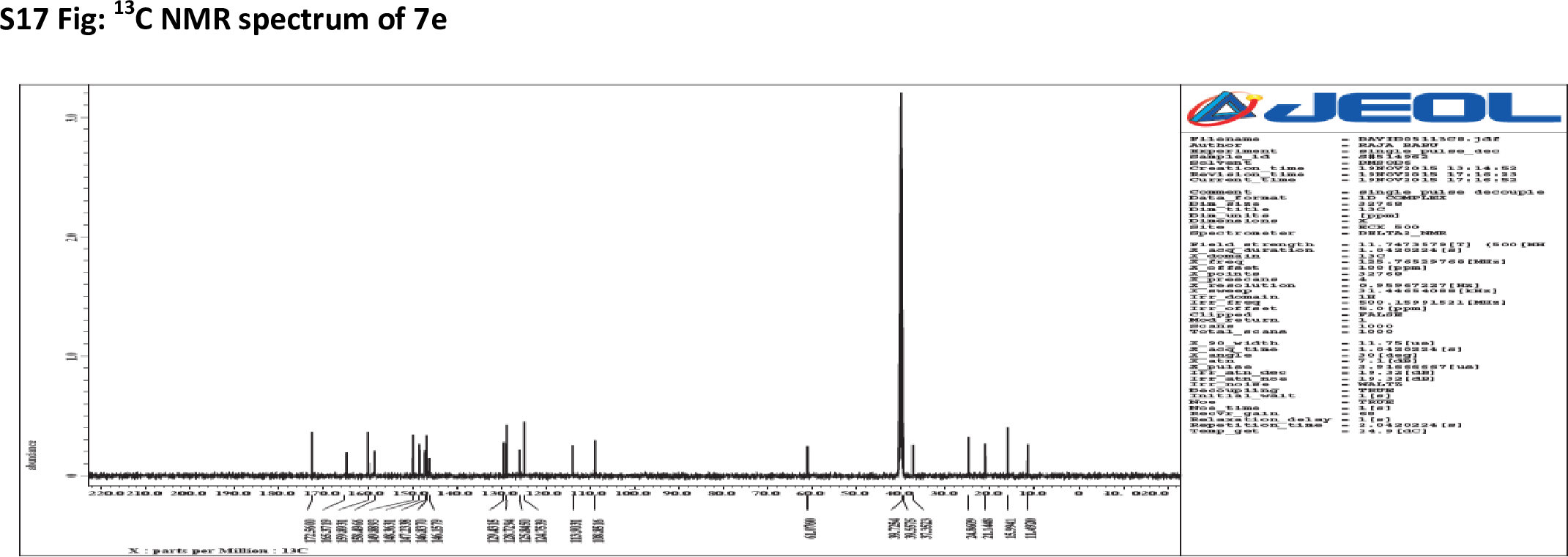

Supplement: S17 Fig — (TIF) [file pone.0183807.s017.tif]

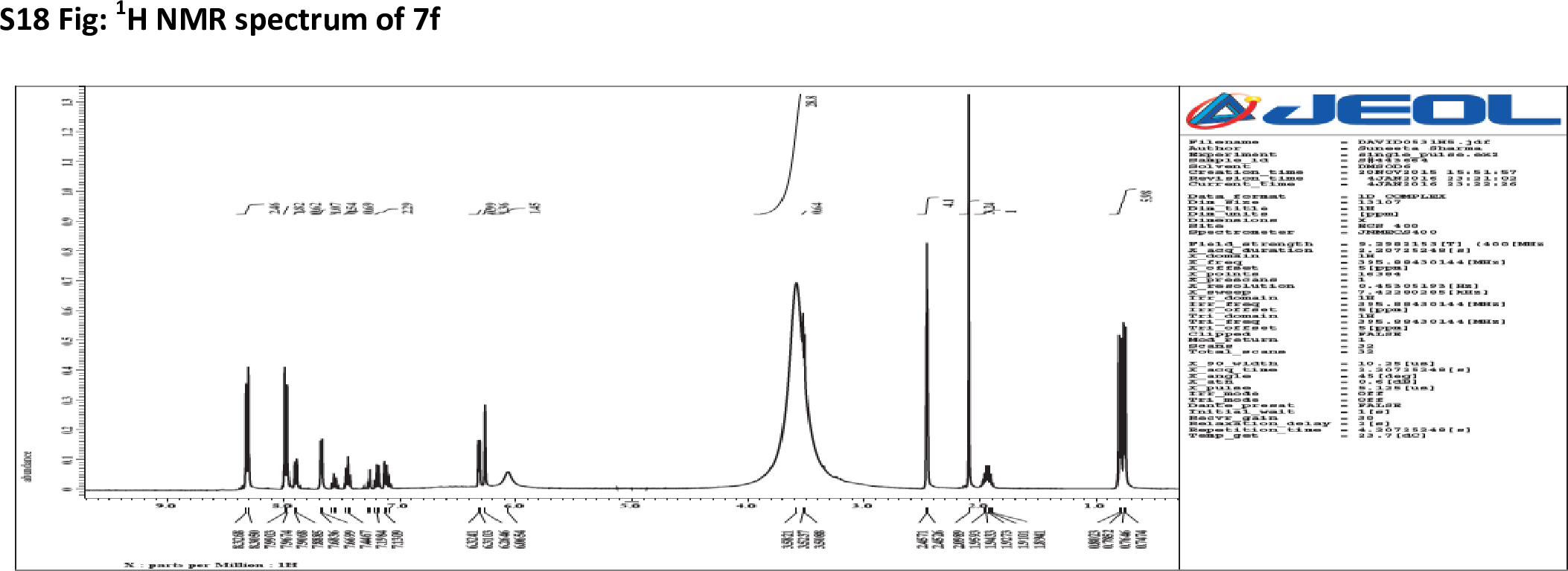

Supplement: S18 Fig — (TIF) [file pone.0183807.s018.tif]

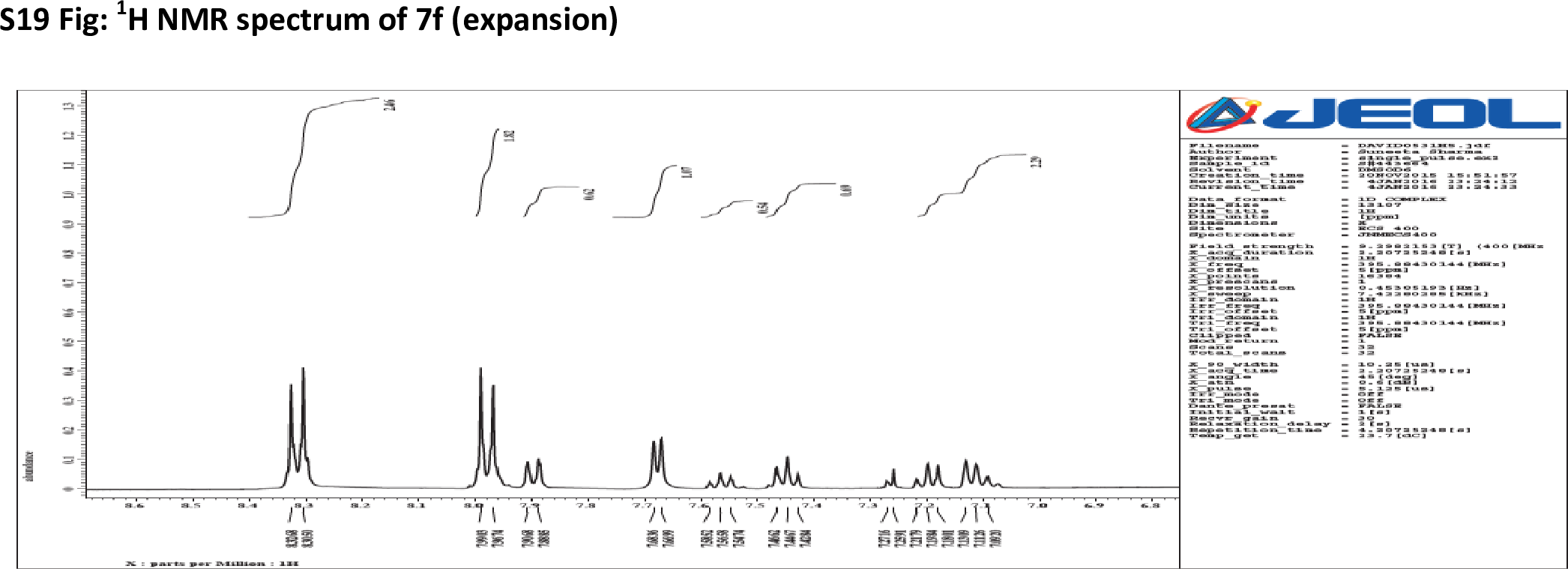

Supplement: S19 Fig — (TIF) [file pone.0183807.s019.tif]

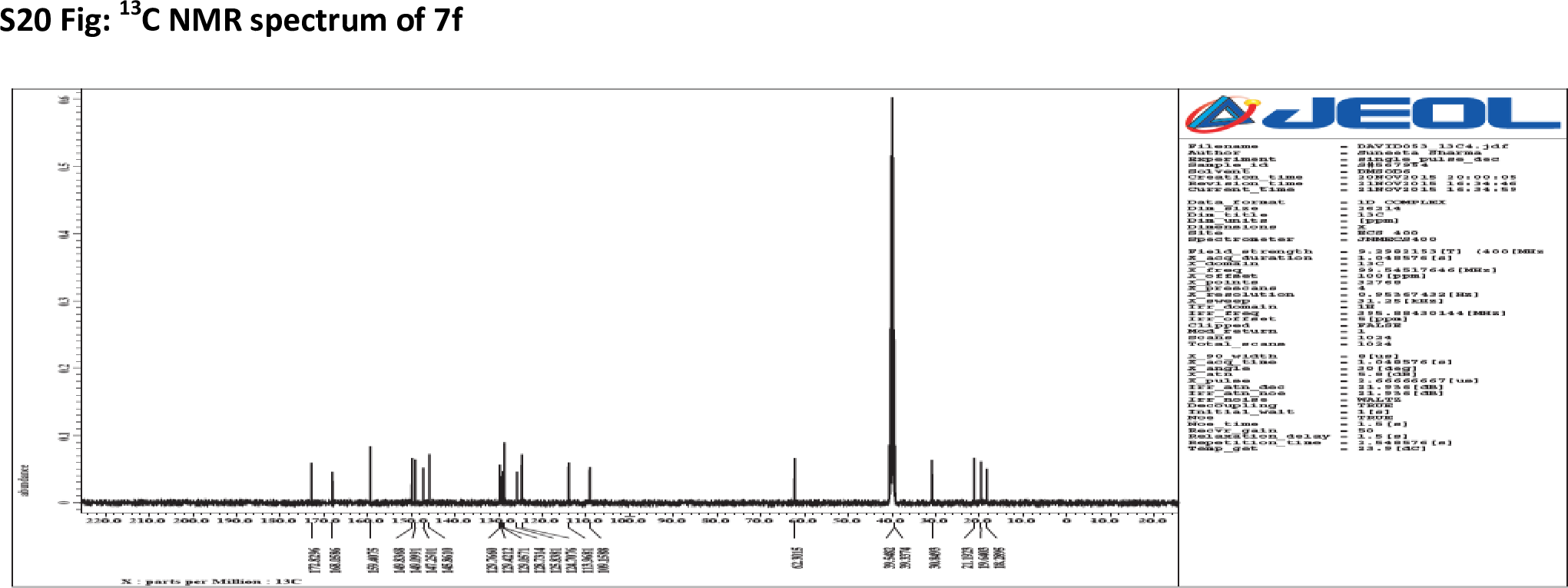

Supplement: S20 Fig — (TIF) [file pone.0183807.s020.tif]

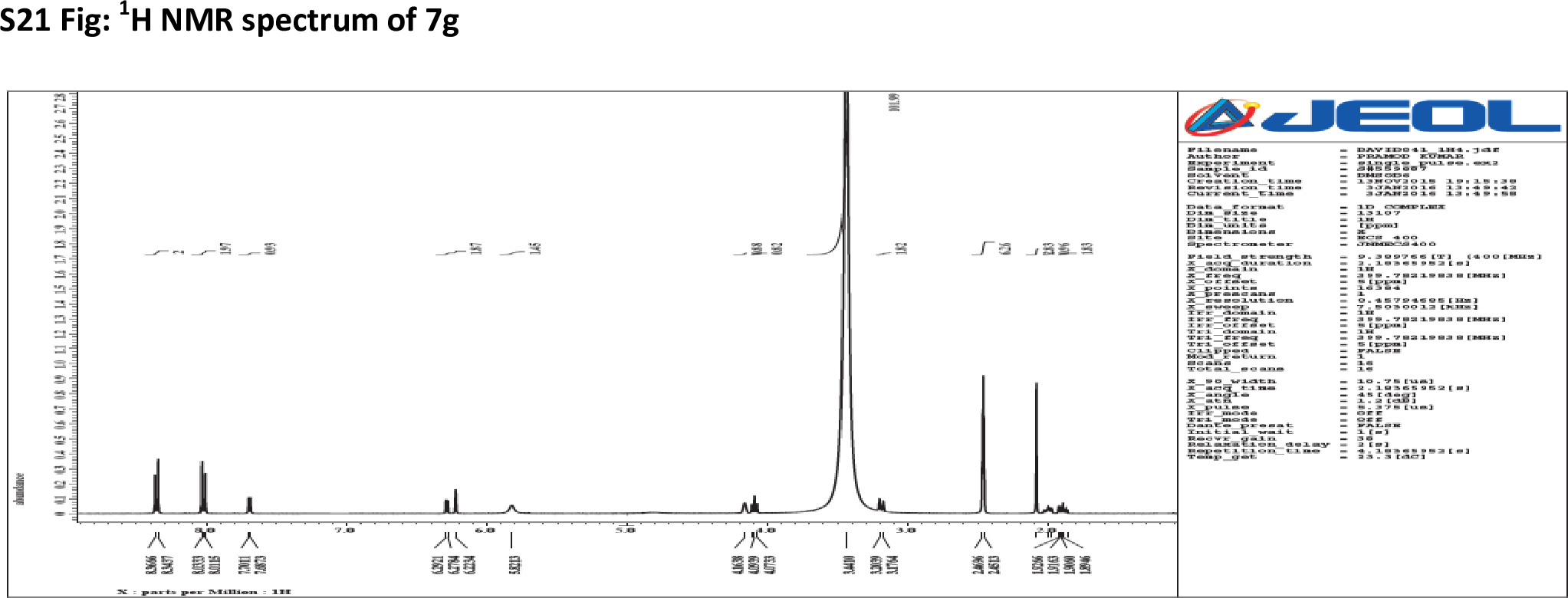

Supplement: S21 Fig — (TIF) [file pone.0183807.s021.tif]

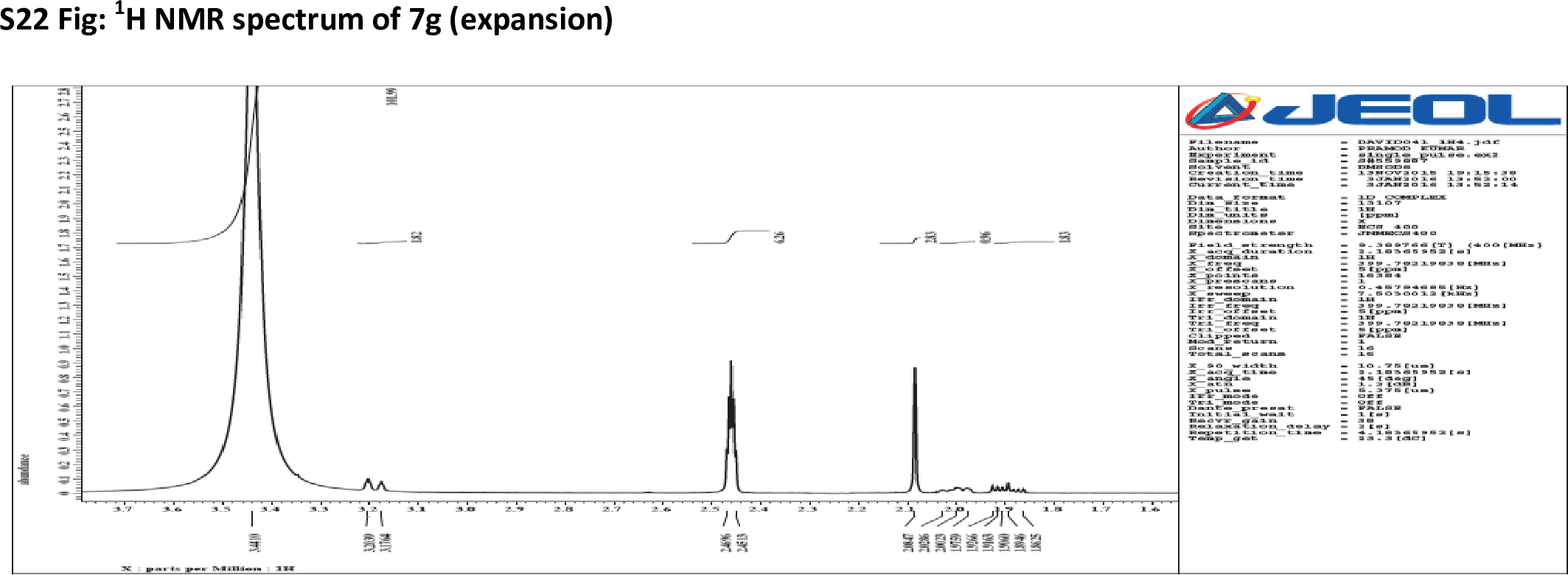

Supplement: S22 Fig — (TIF) [file pone.0183807.s022.tif]

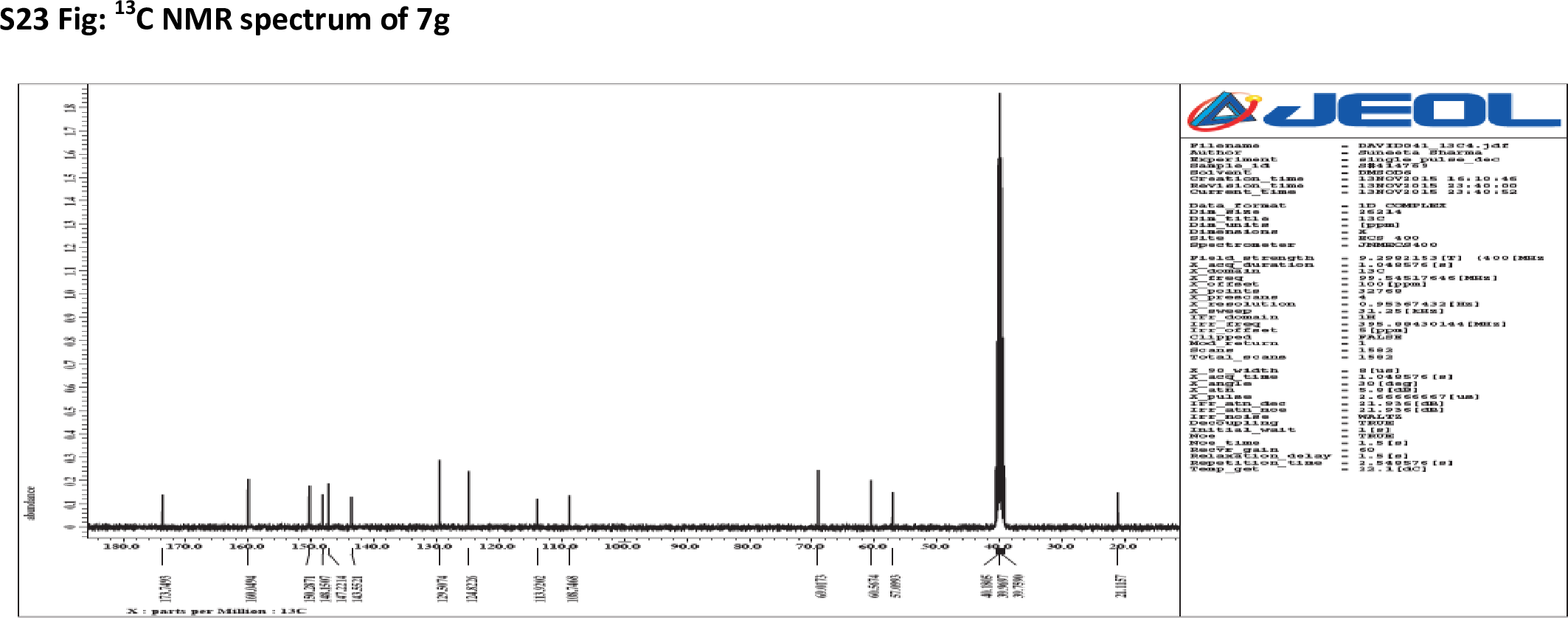

Supplement: S23 Fig — (TIF) [file pone.0183807.s023.tif]

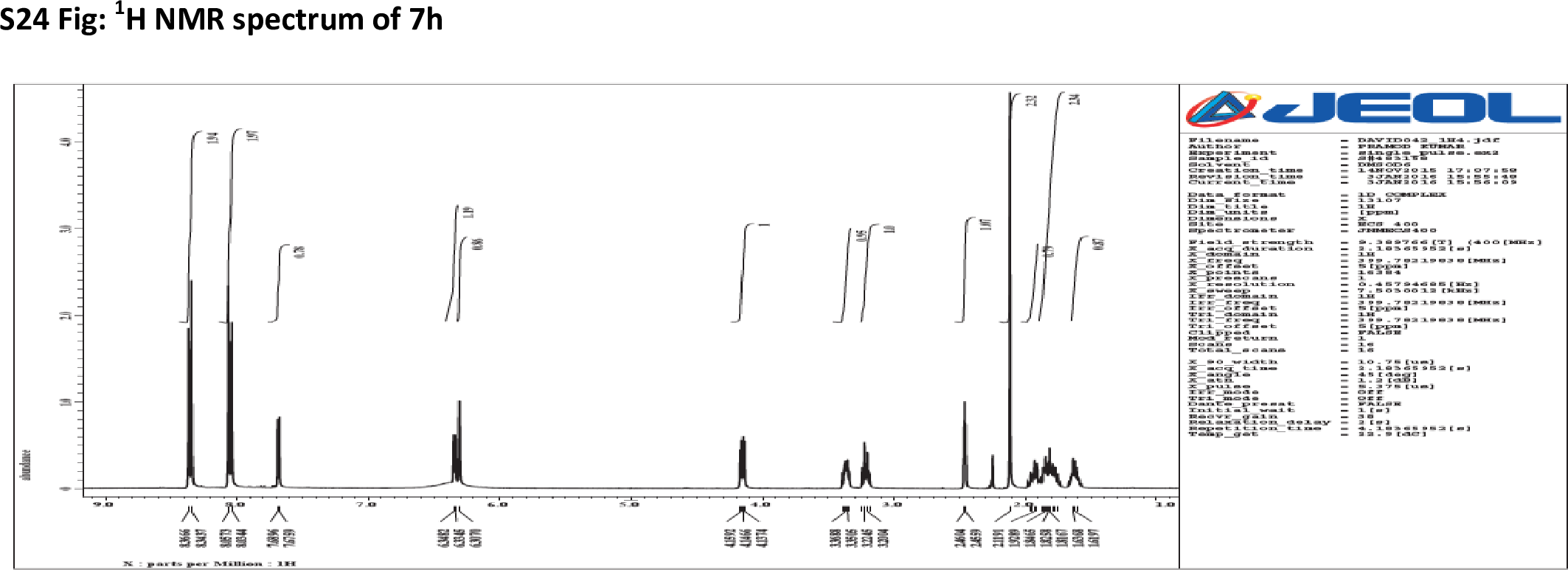

Supplement: S24 Fig — (TIF) [file pone.0183807.s024.tif]

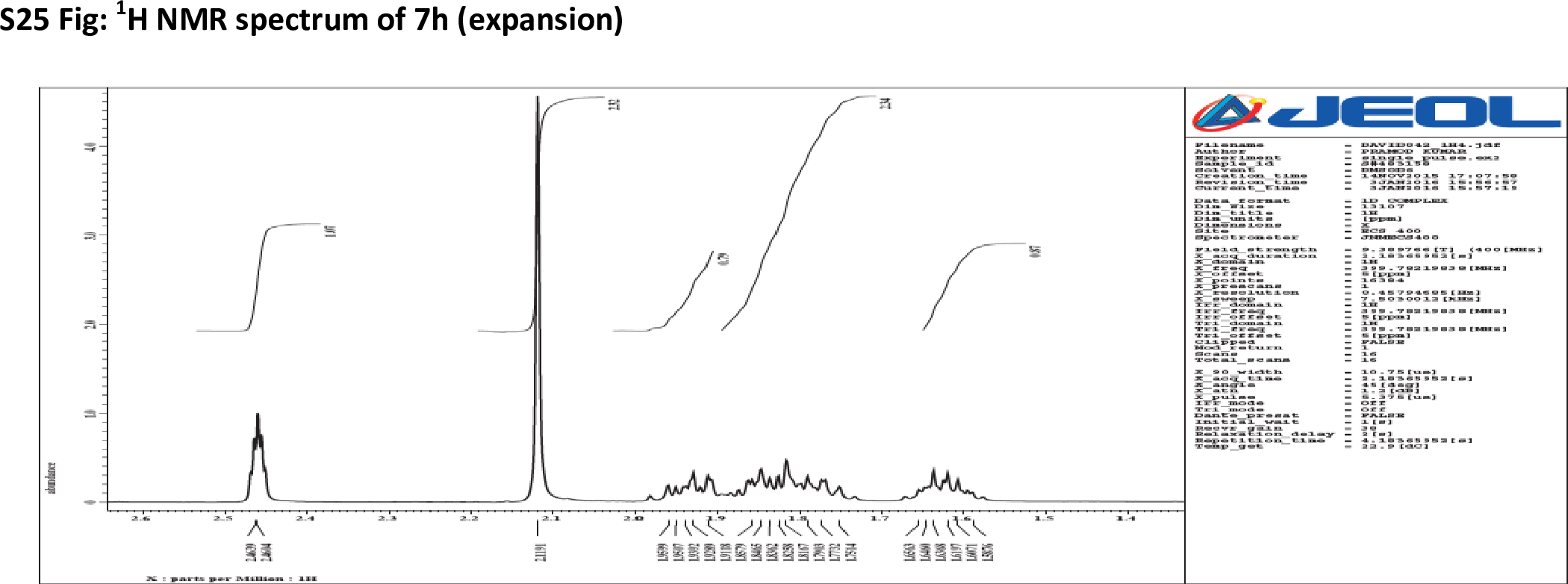

Supplement: S25 Fig — (TIF) [file pone.0183807.s025.tif]

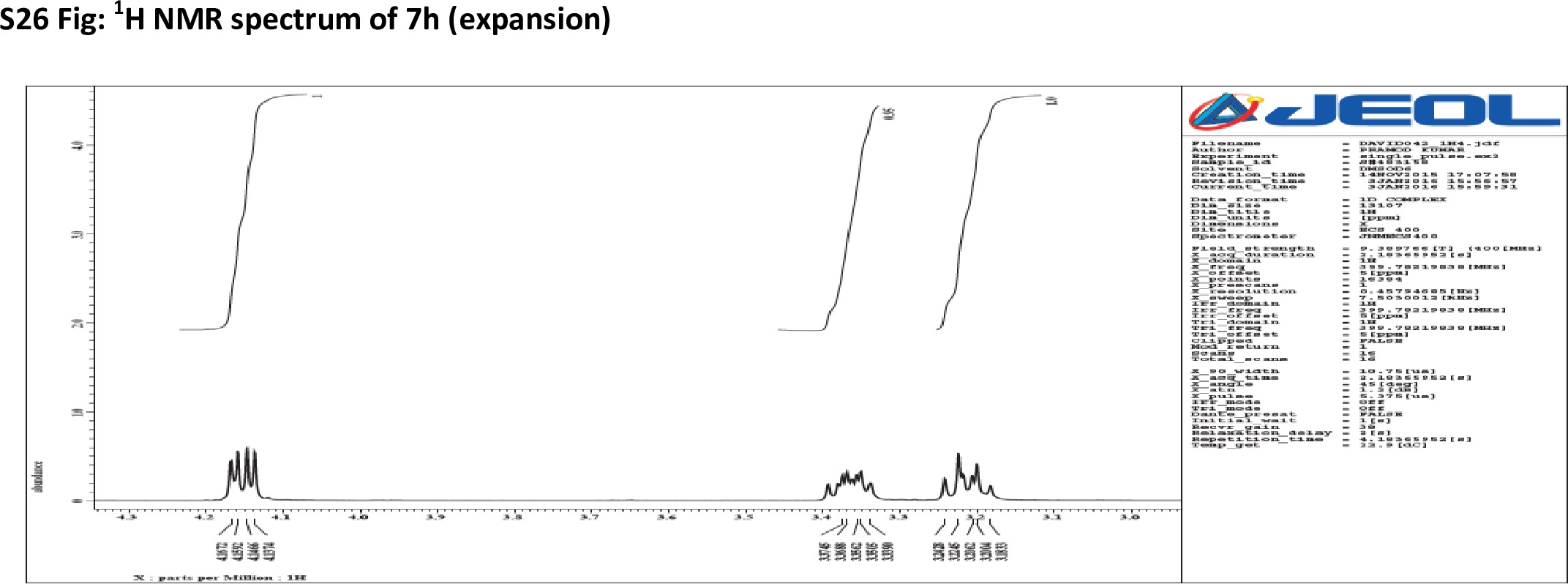

Supplement: S26 Fig — (TIF) [file pone.0183807.s026.tif]

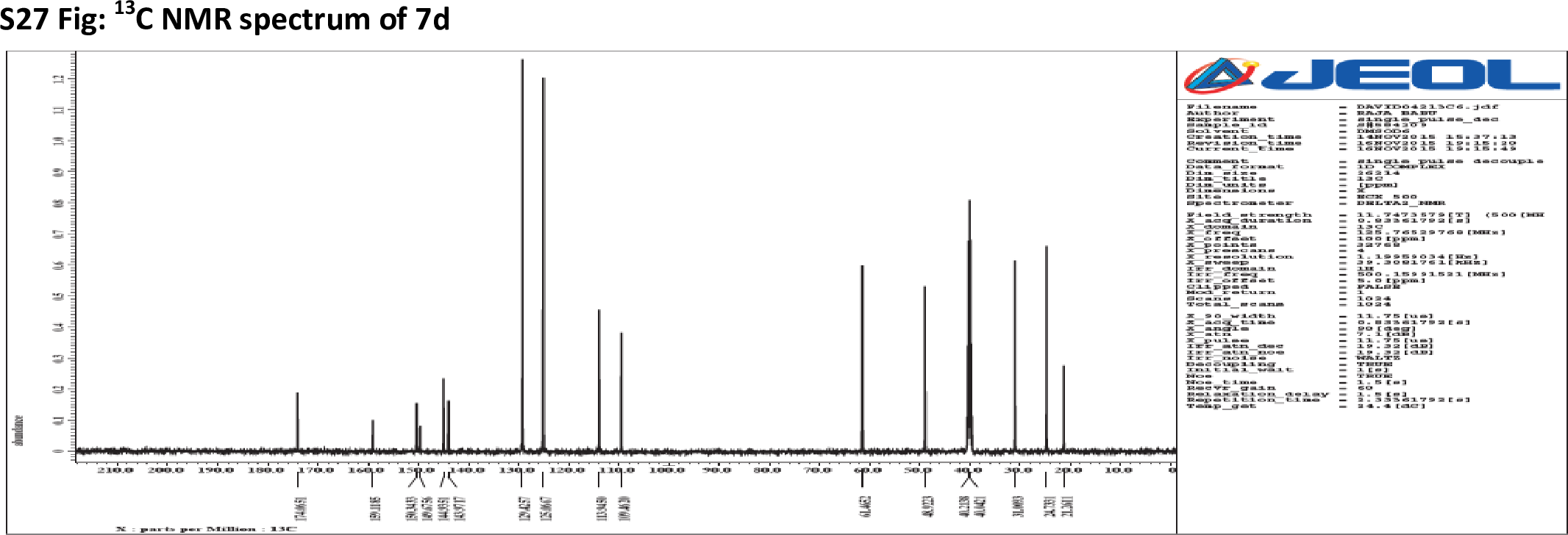

Supplement: S27 Fig — (TIF) [file pone.0183807.s027.tif]

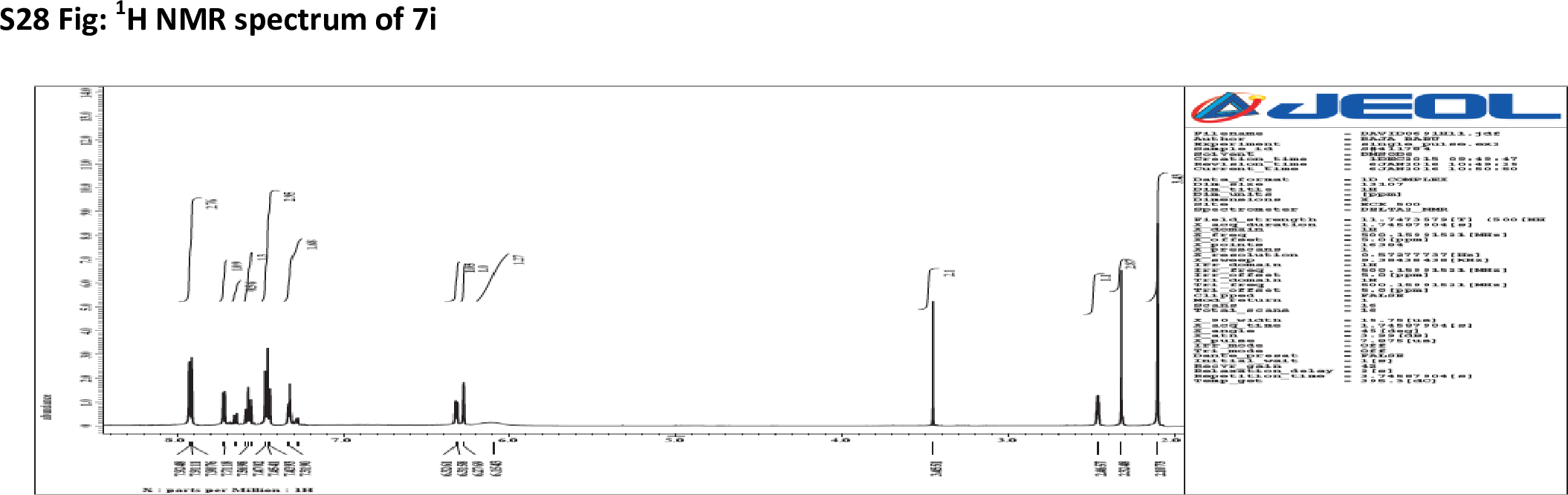

Supplement: S28 Fig — (TIF) [file pone.0183807.s028.tif]

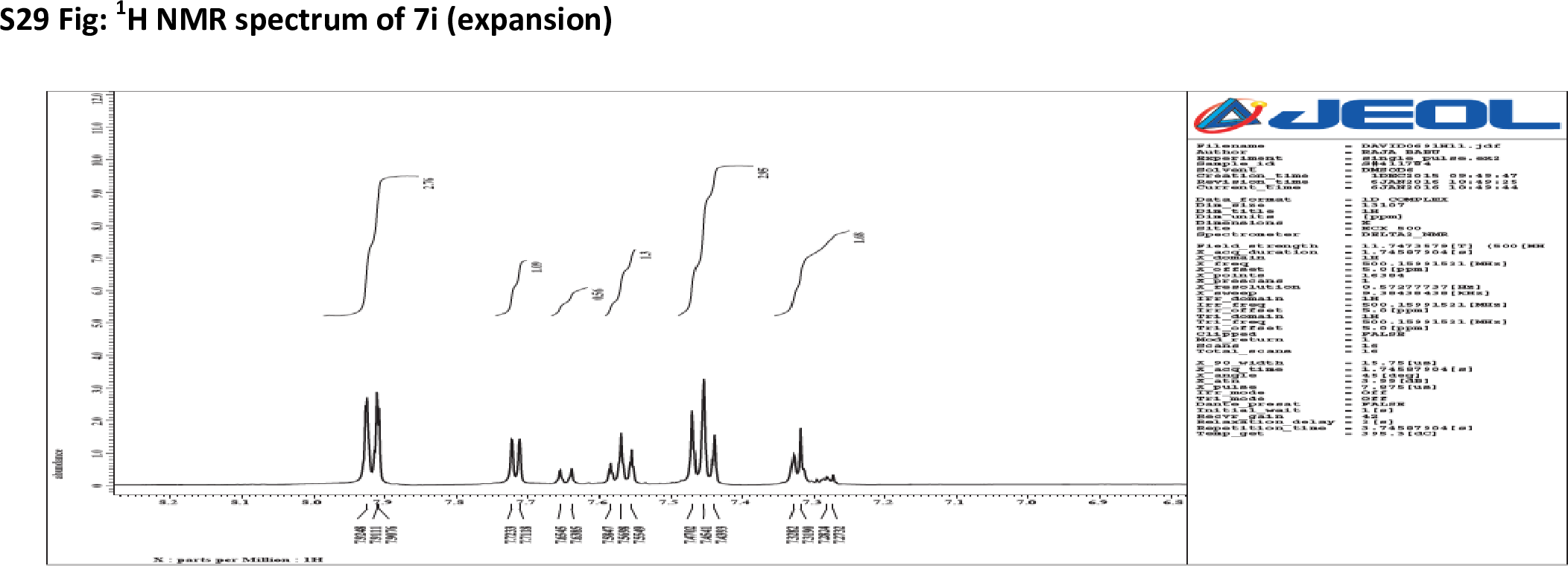

Supplement: S29 Fig — (TIF) [file pone.0183807.s029.tif]

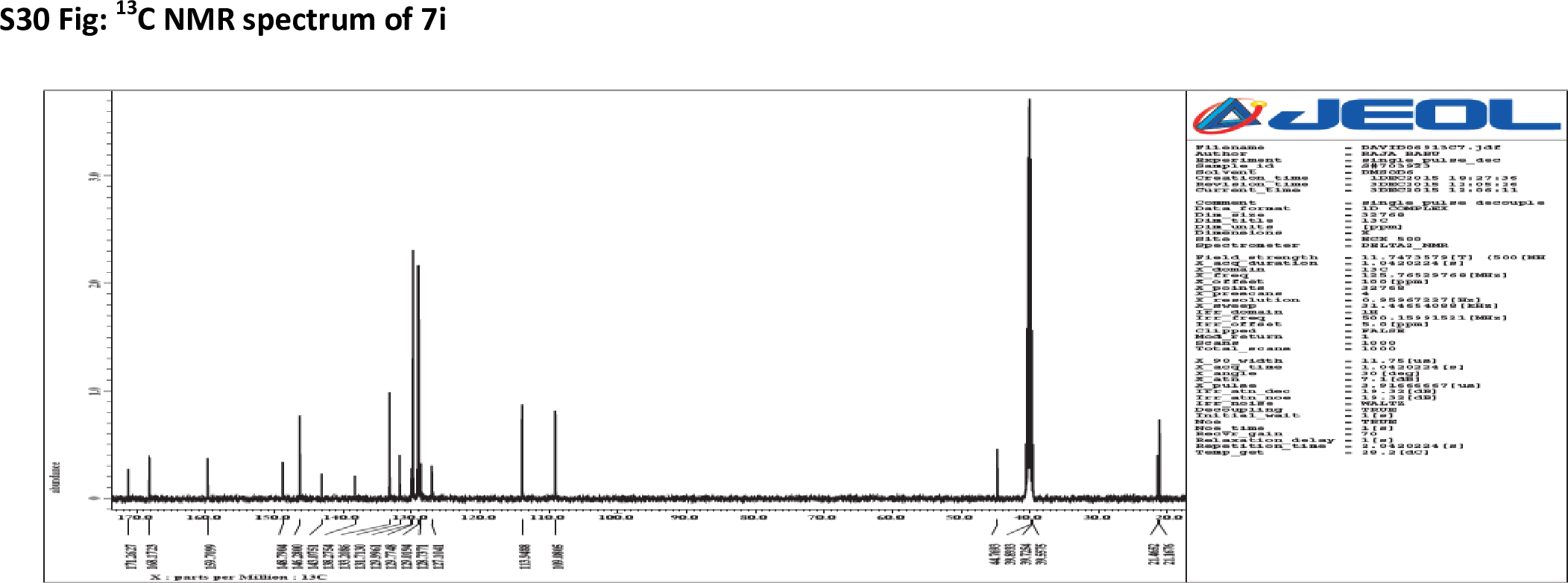

Supplement: S30 Fig — (TIF) [file pone.0183807.s030.tif]

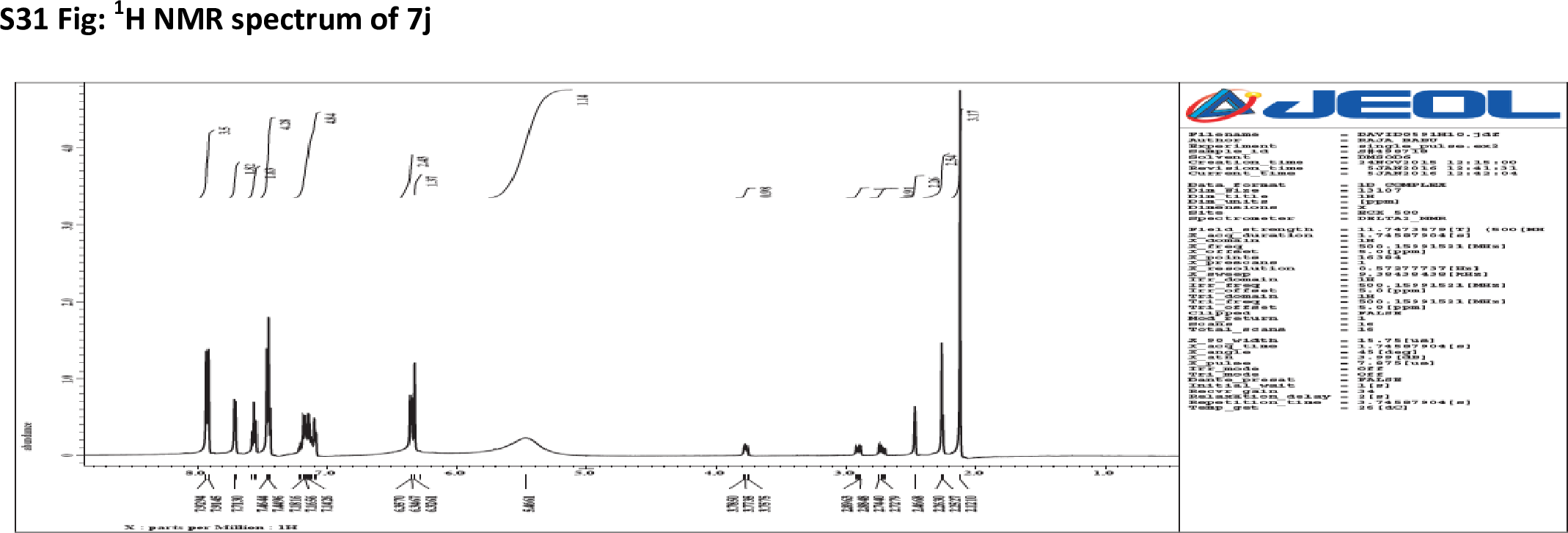

Supplement: S31 Fig — (TIF) [file pone.0183807.s031.tif]

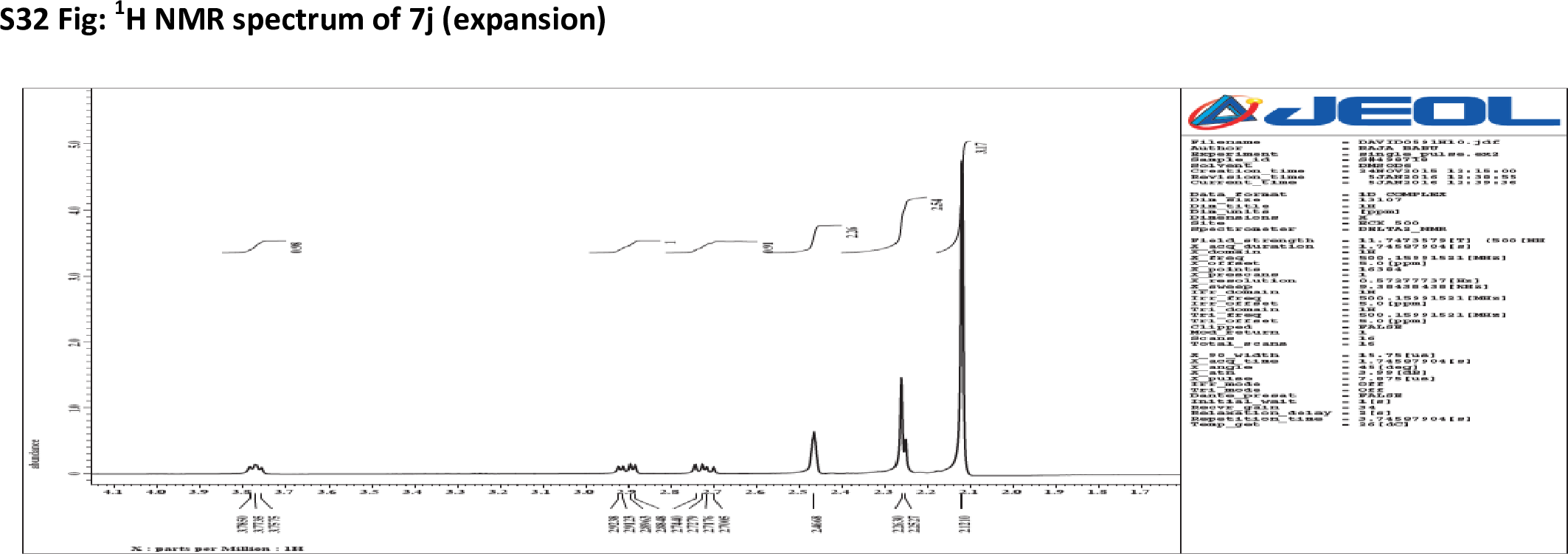

Supplement: S32 Fig — (TIF) [file pone.0183807.s032.tif]

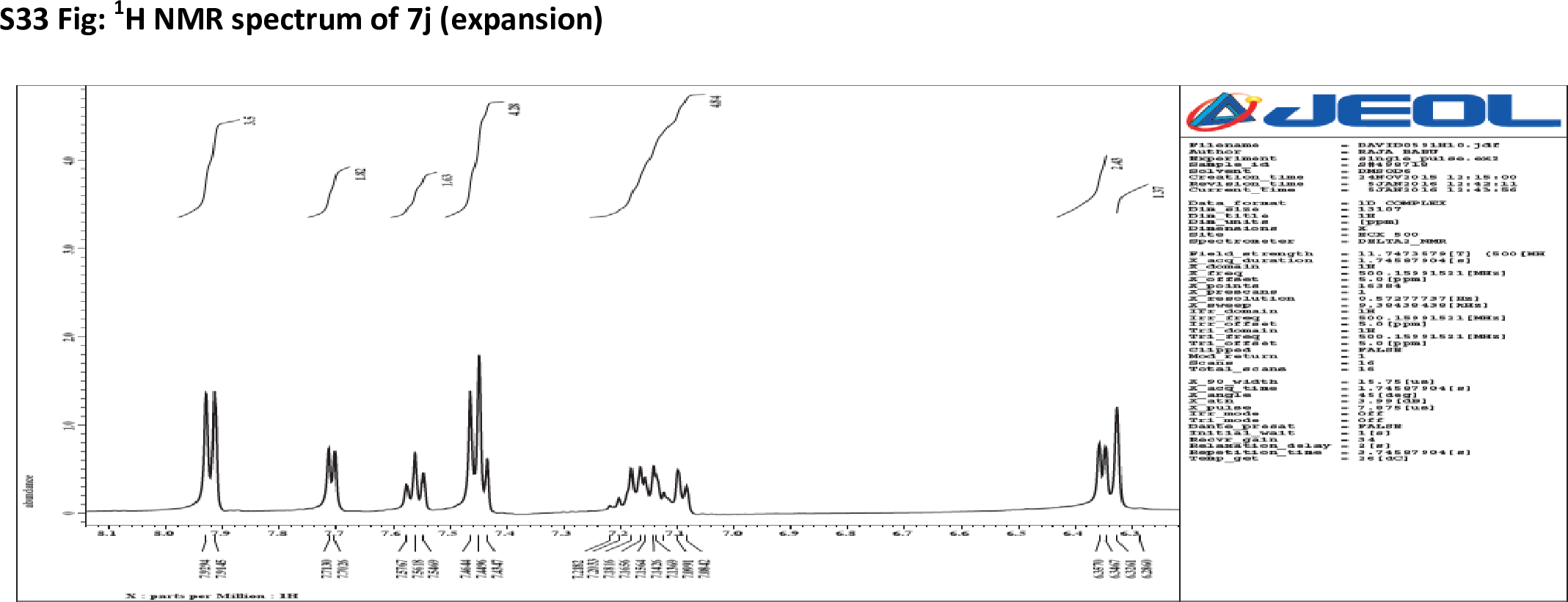

Supplement: S33 Fig — (TIF) [file pone.0183807.s033.tif]

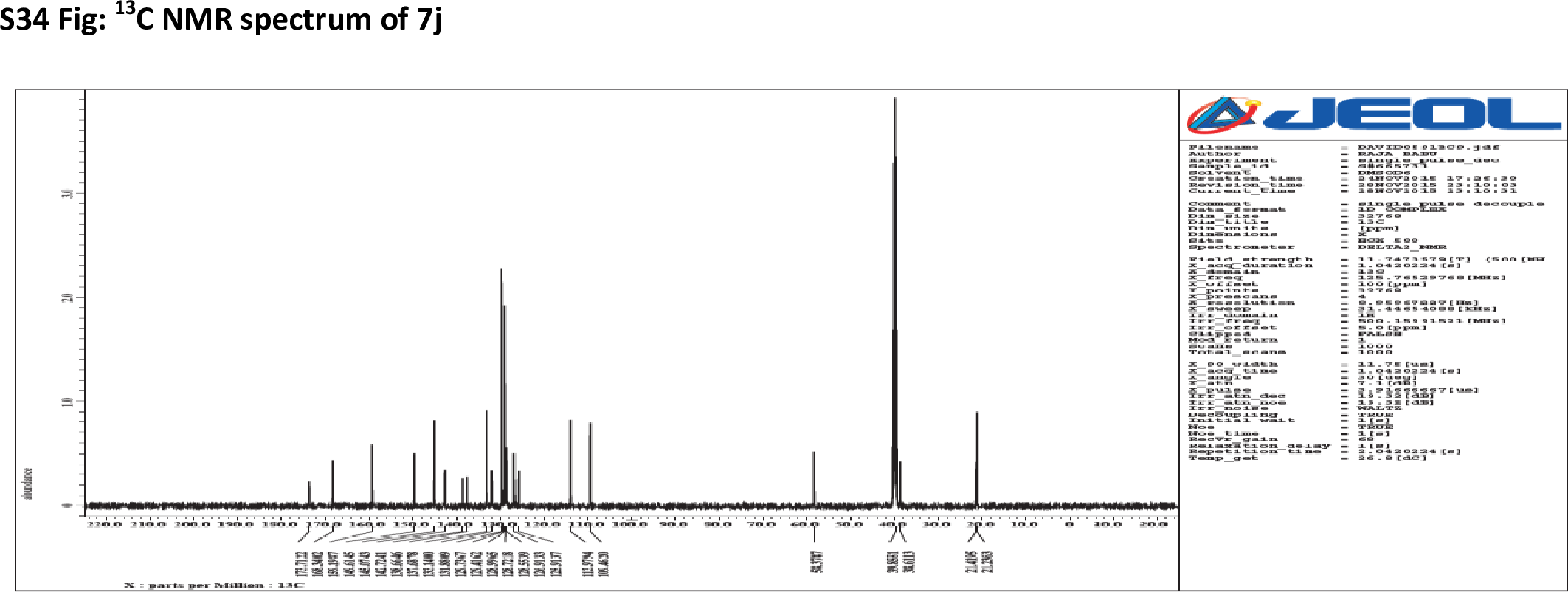

Supplement: S34 Fig — (TIF) [file pone.0183807.s034.tif]

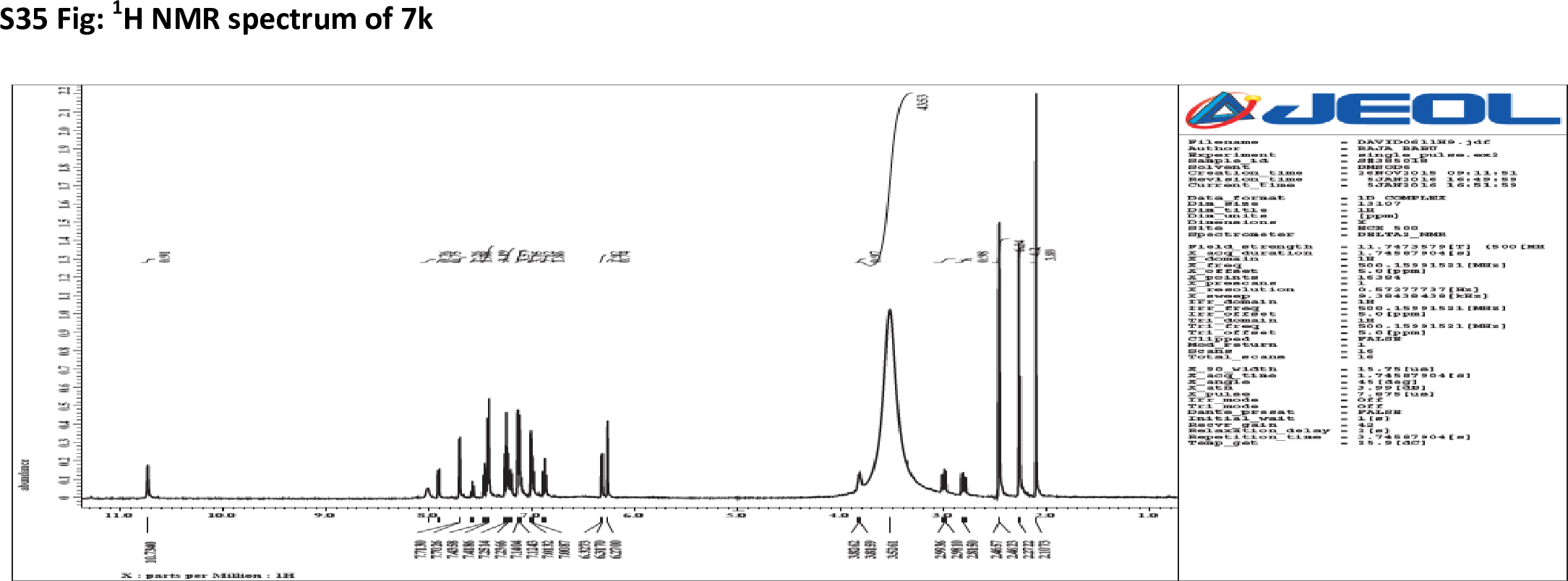

Supplement: S35 Fig — (TIF) [file pone.0183807.s035.tif]

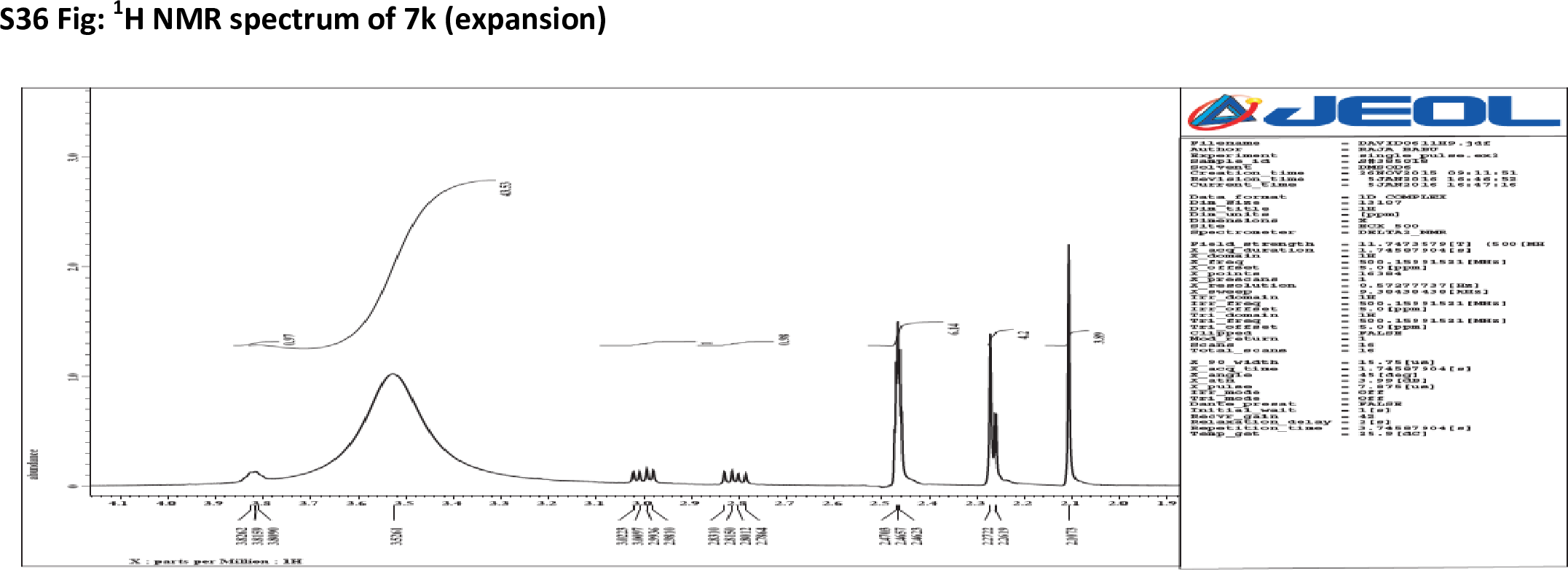

Supplement: S36 Fig — (TIF) [file pone.0183807.s036.tif]

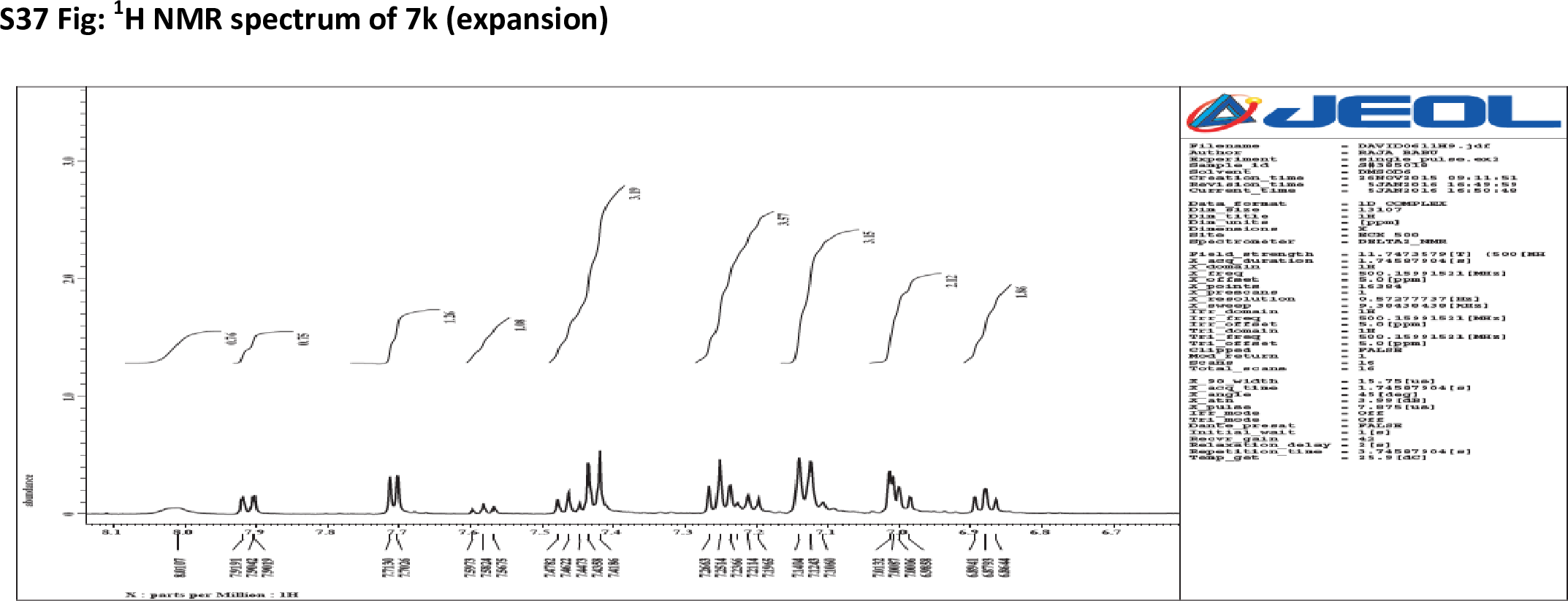

Supplement: S37 Fig — (TIF) [file pone.0183807.s037.tif]

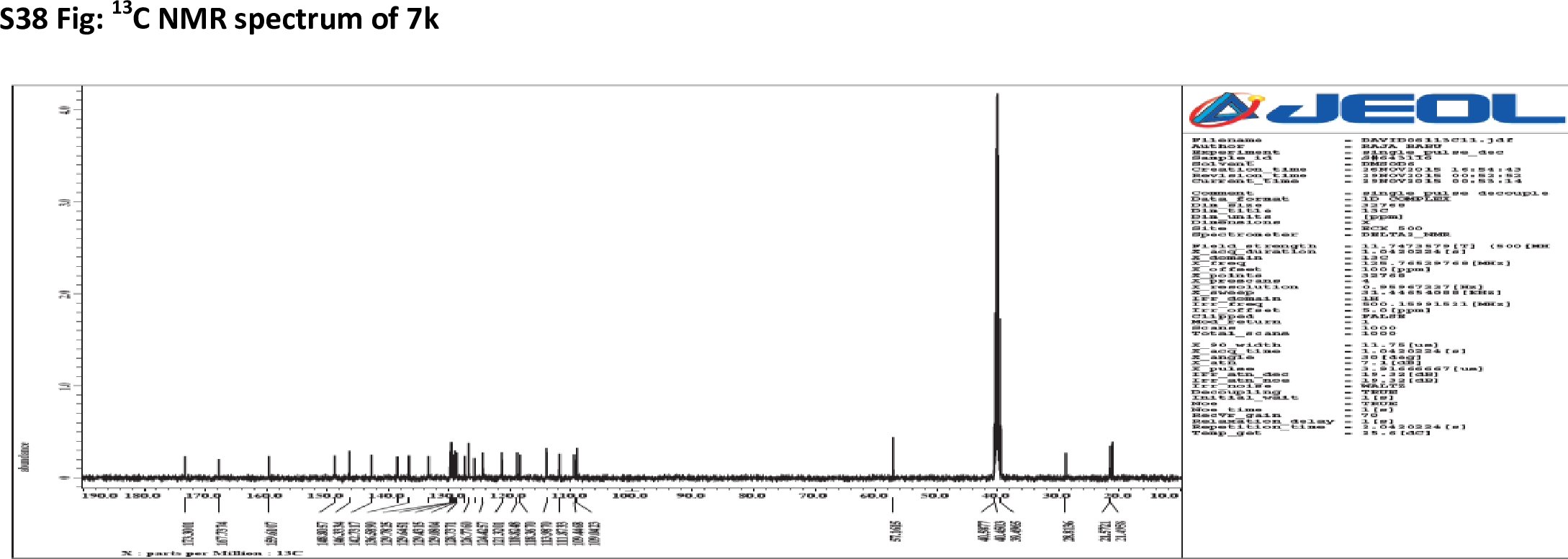

Supplement: S38 Fig — (TIF) [file pone.0183807.s038.tif]

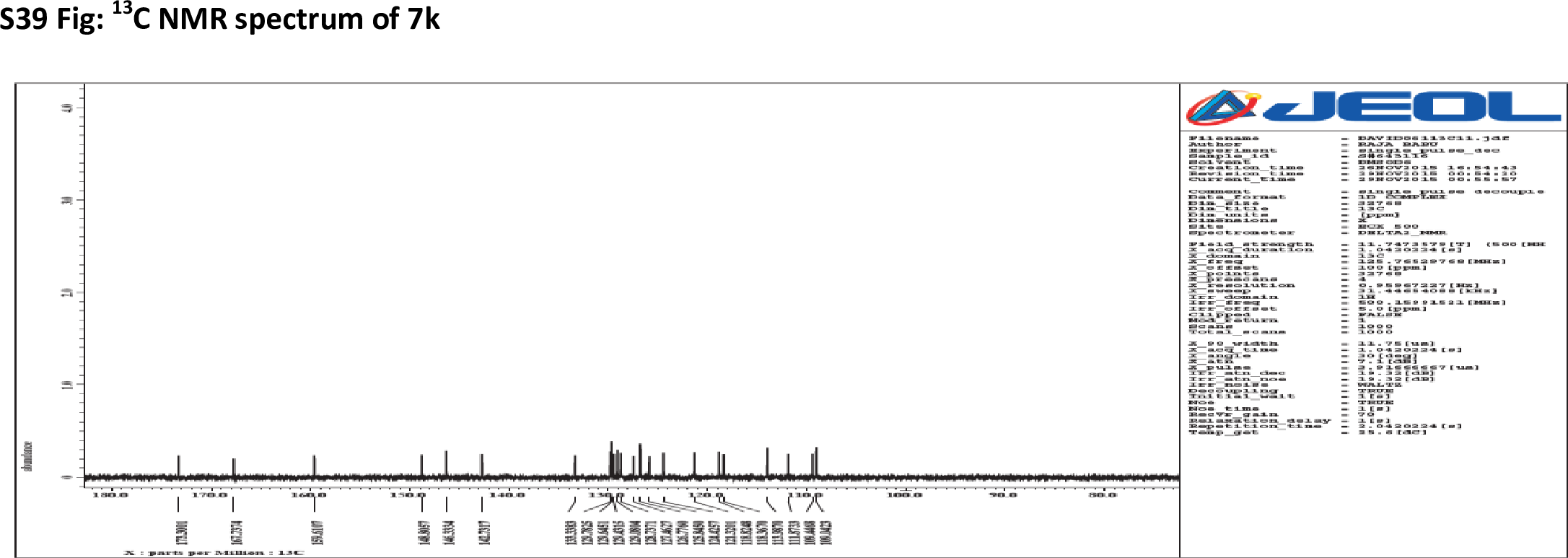

Supplement: S39 Fig — (TIF) [file pone.0183807.s039.tif]

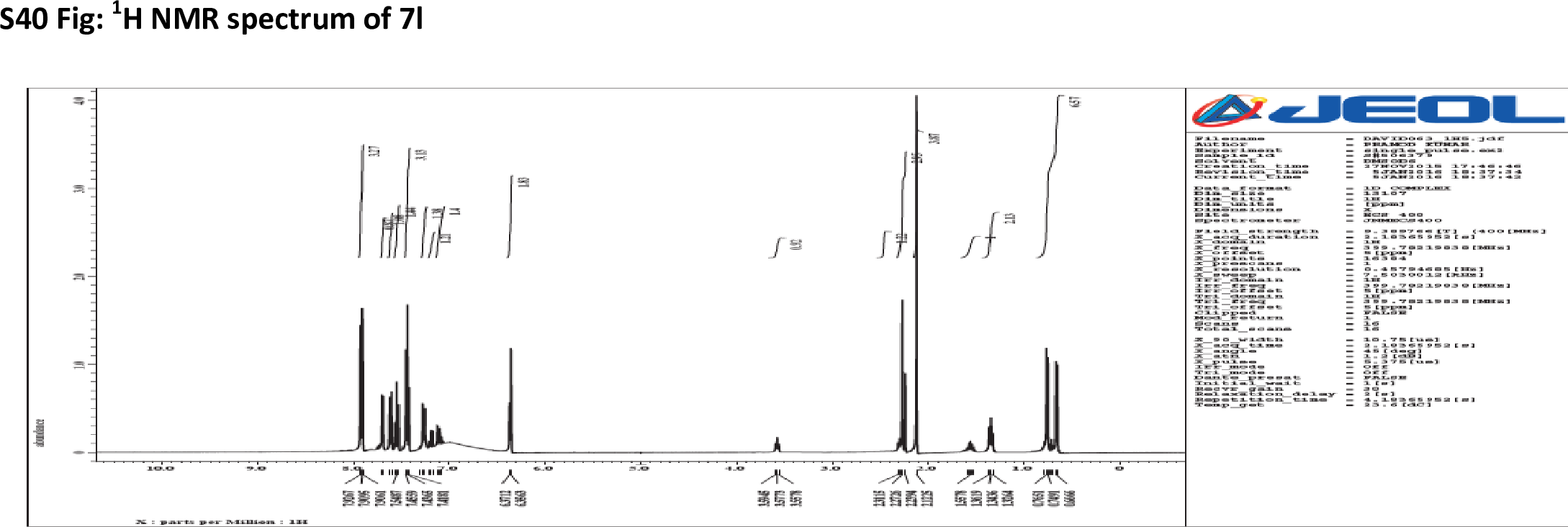

Supplement: S40 Fig — (TIF) [file pone.0183807.s040.tif]

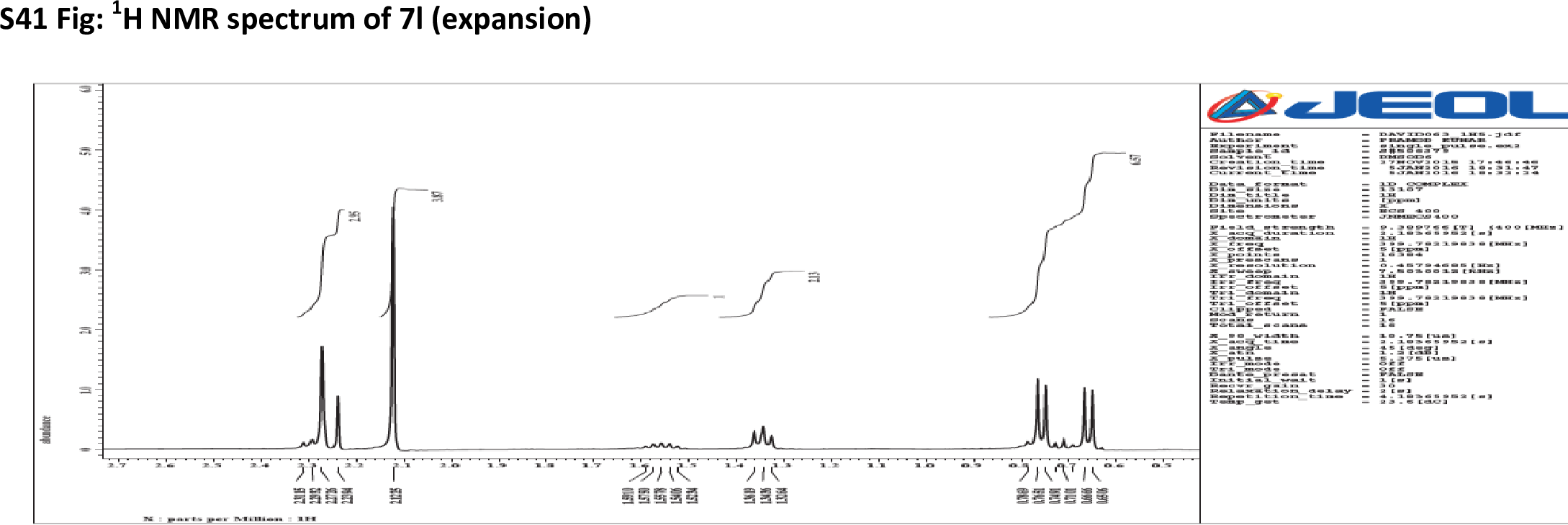

Supplement: S41 Fig — (TIF) [file pone.0183807.s041.tif]

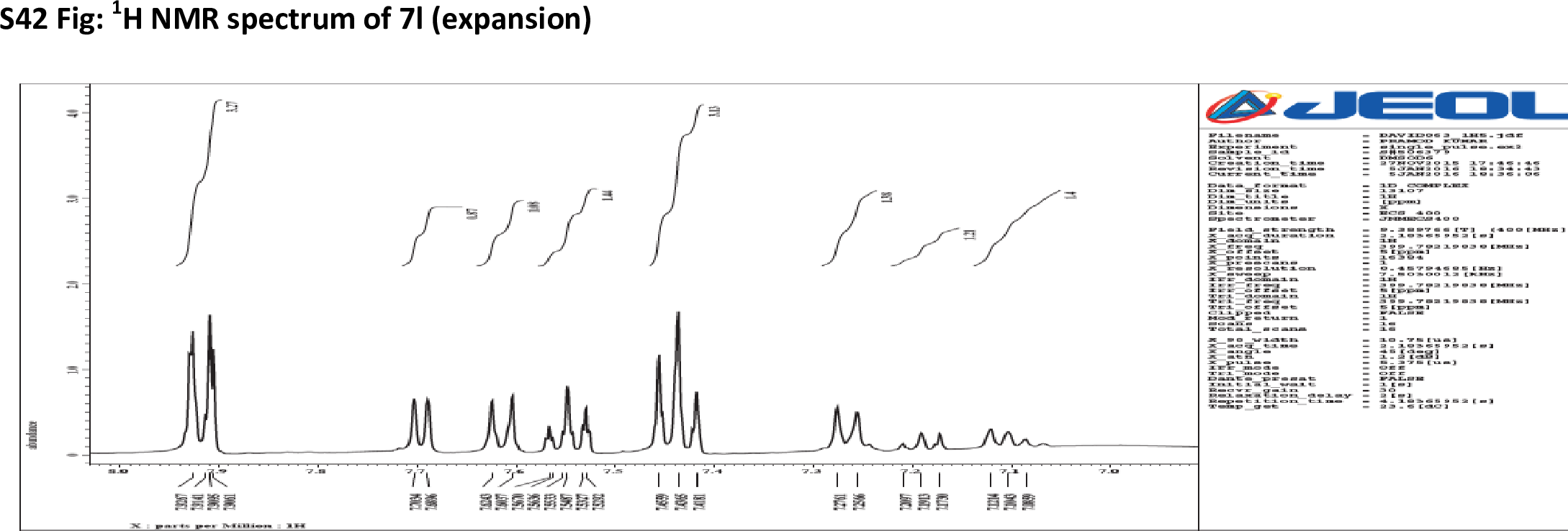

Supplement: S42 Fig — (TIF) [file pone.0183807.s042.tif]

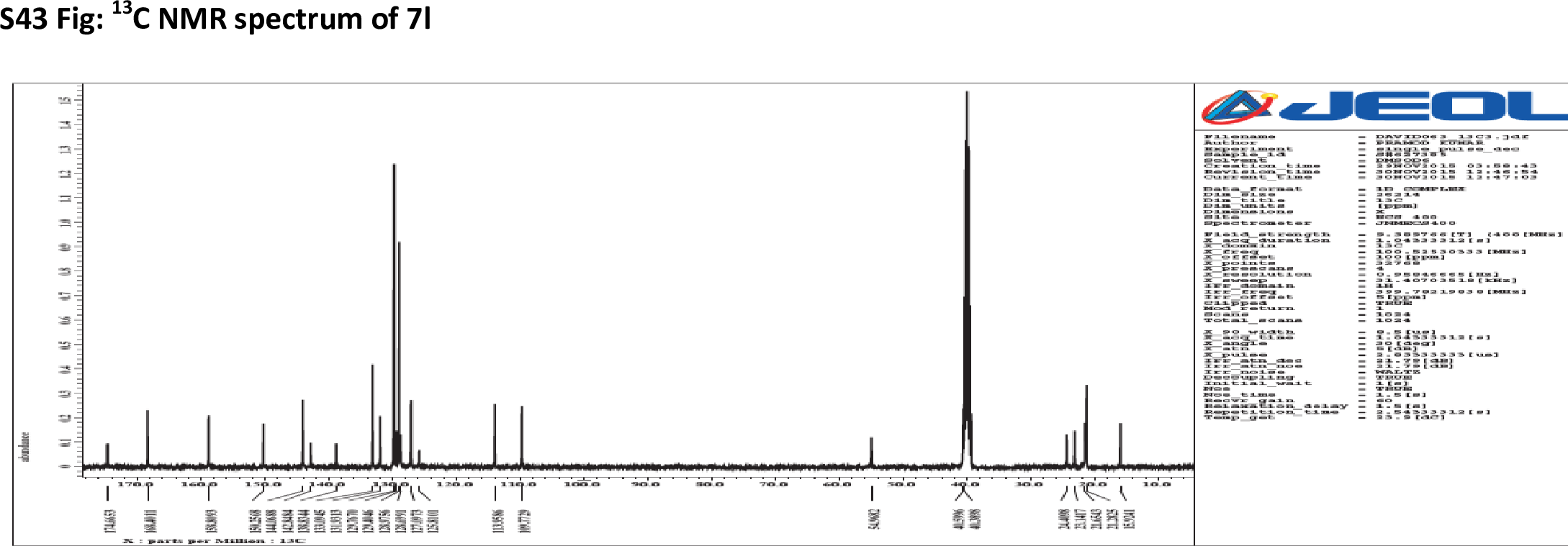

Supplement: S43 Fig — (TIF) [file pone.0183807.s043.tif]

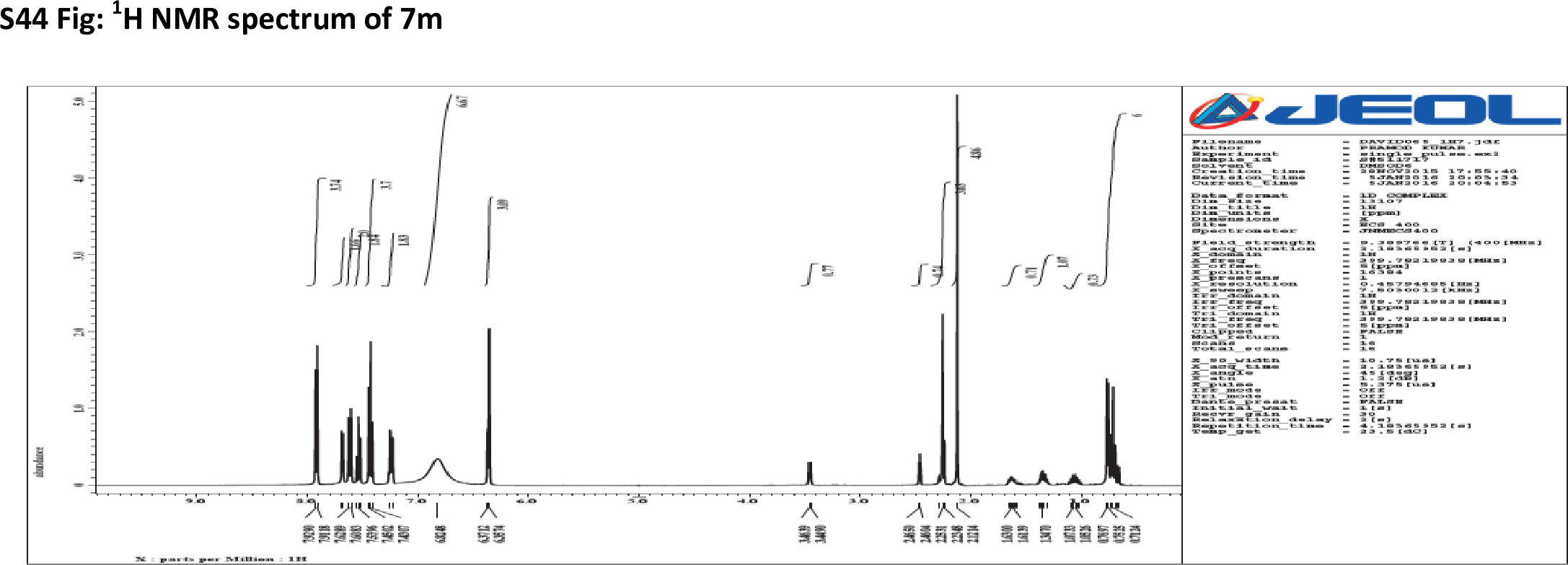

Supplement: S44 Fig — (TIF) [file pone.0183807.s044.tif]

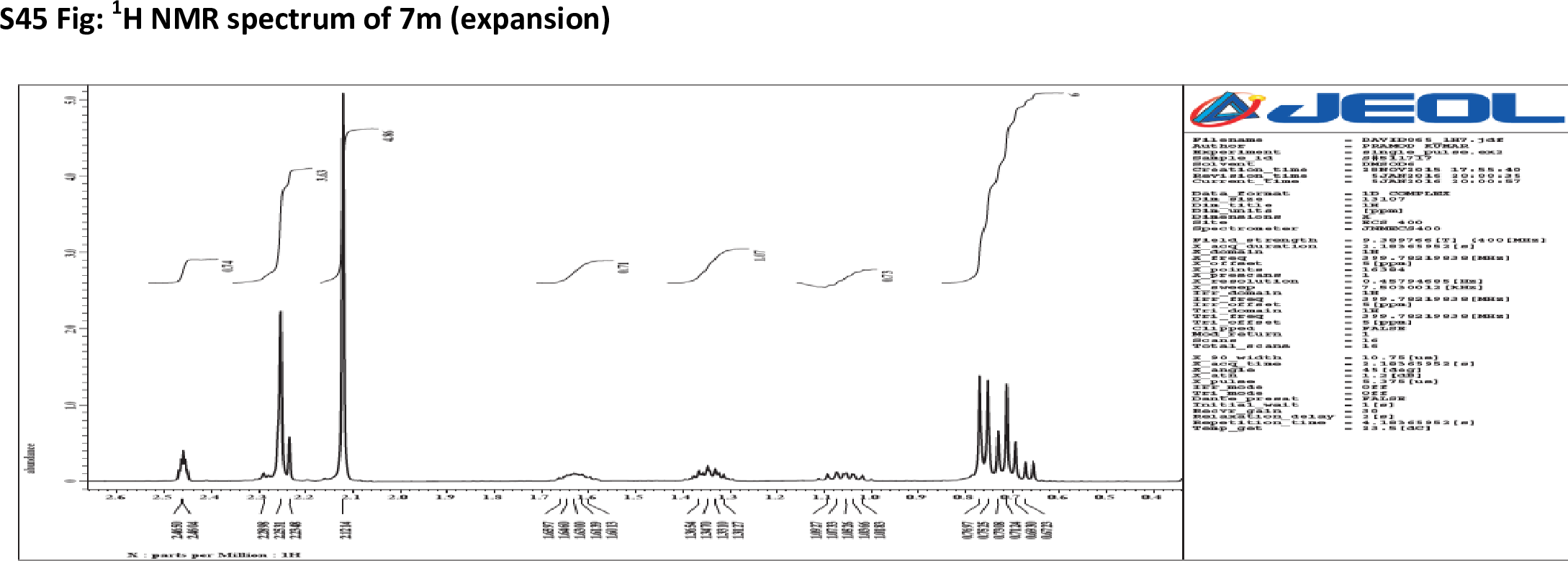

Supplement: S45 Fig — (TIF) [file pone.0183807.s045.tif]

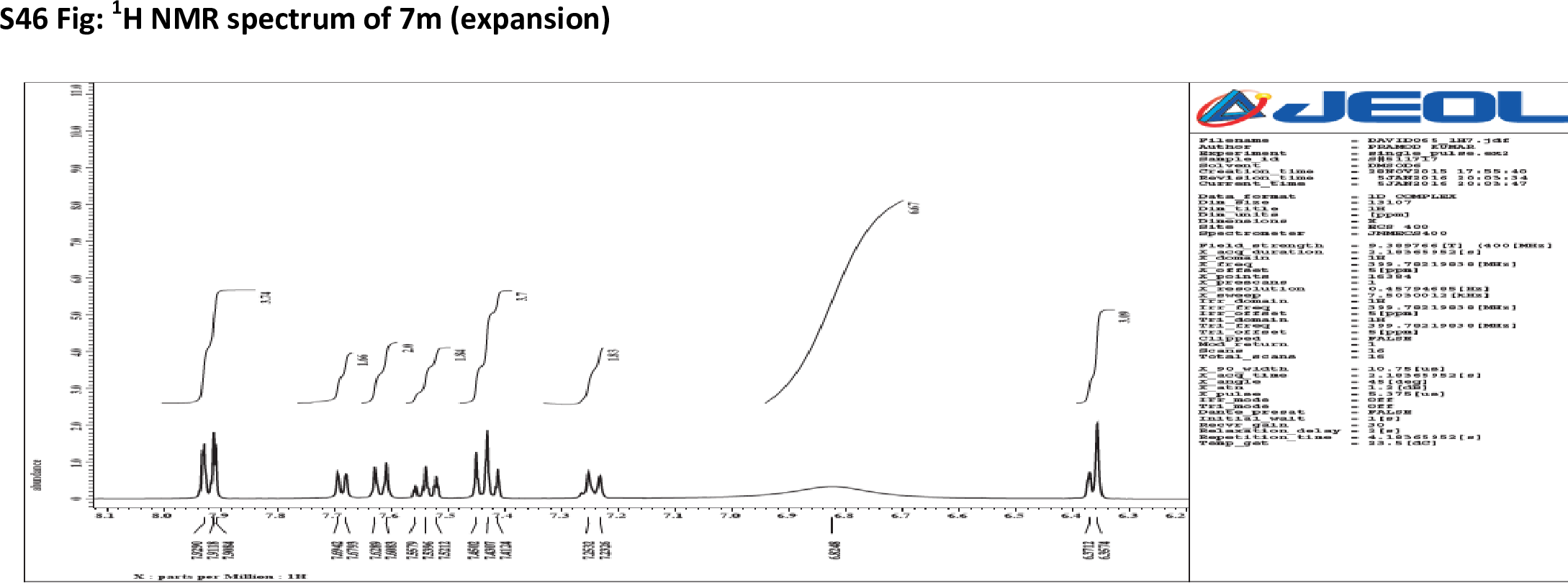

Supplement: S46 Fig — (TIF) [file pone.0183807.s046.tif]

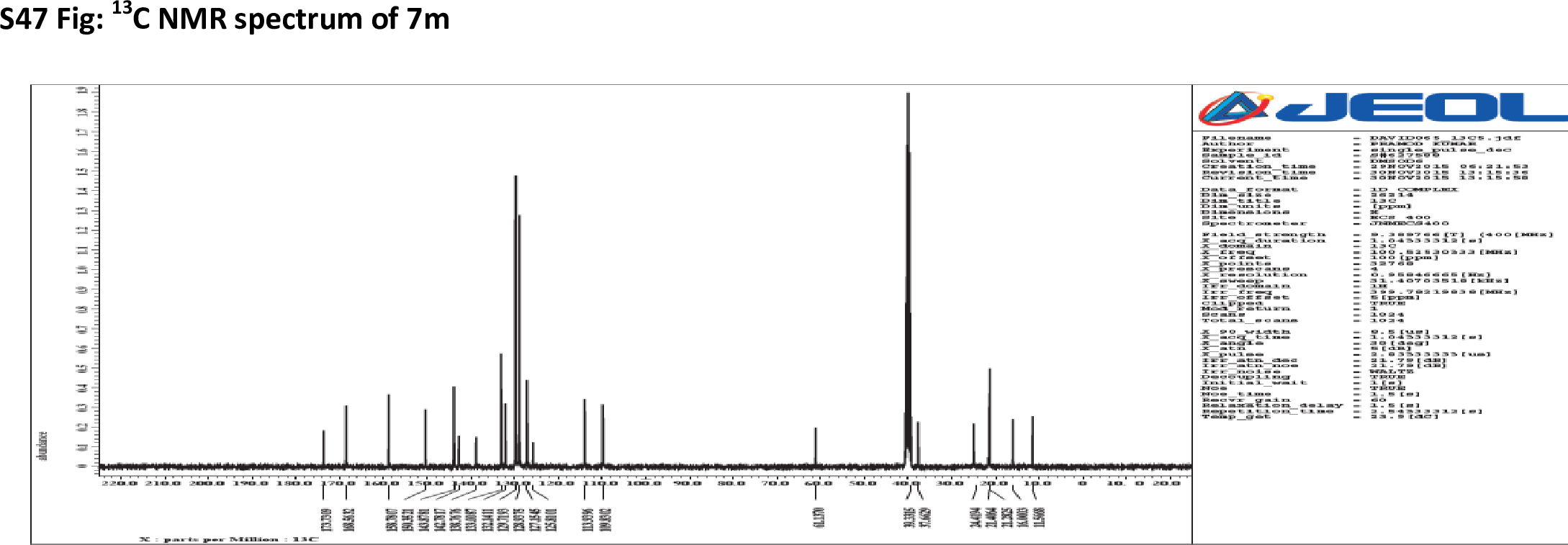

Supplement: S47 Fig — (TIF) [file pone.0183807.s047.tif]

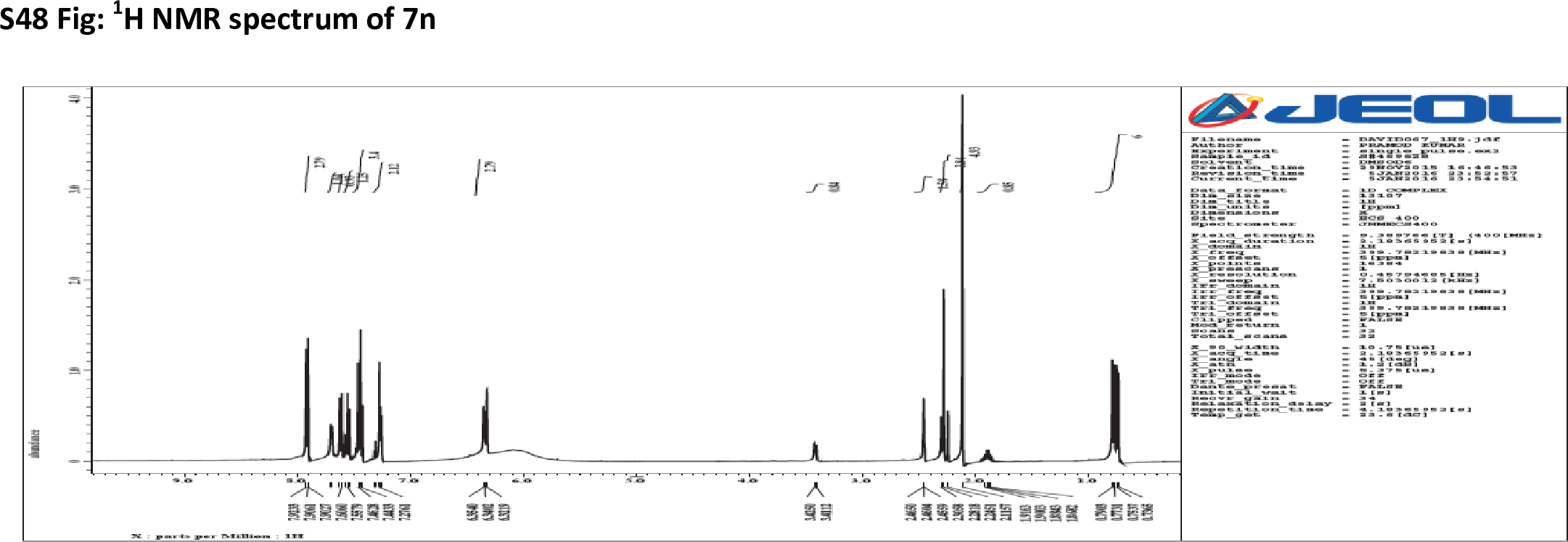

Supplement: S48 Fig — (TIF) [file pone.0183807.s048.tif]

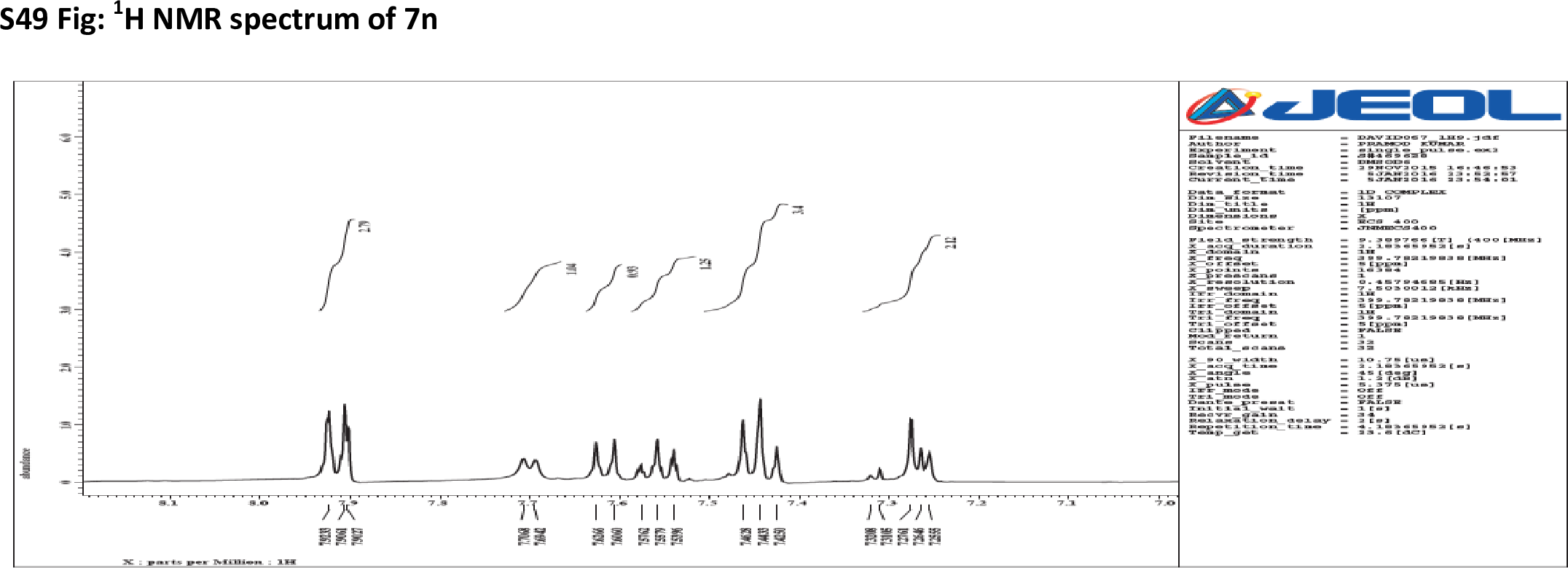

Supplement: S49 Fig — (TIF) [file pone.0183807.s049.tif]

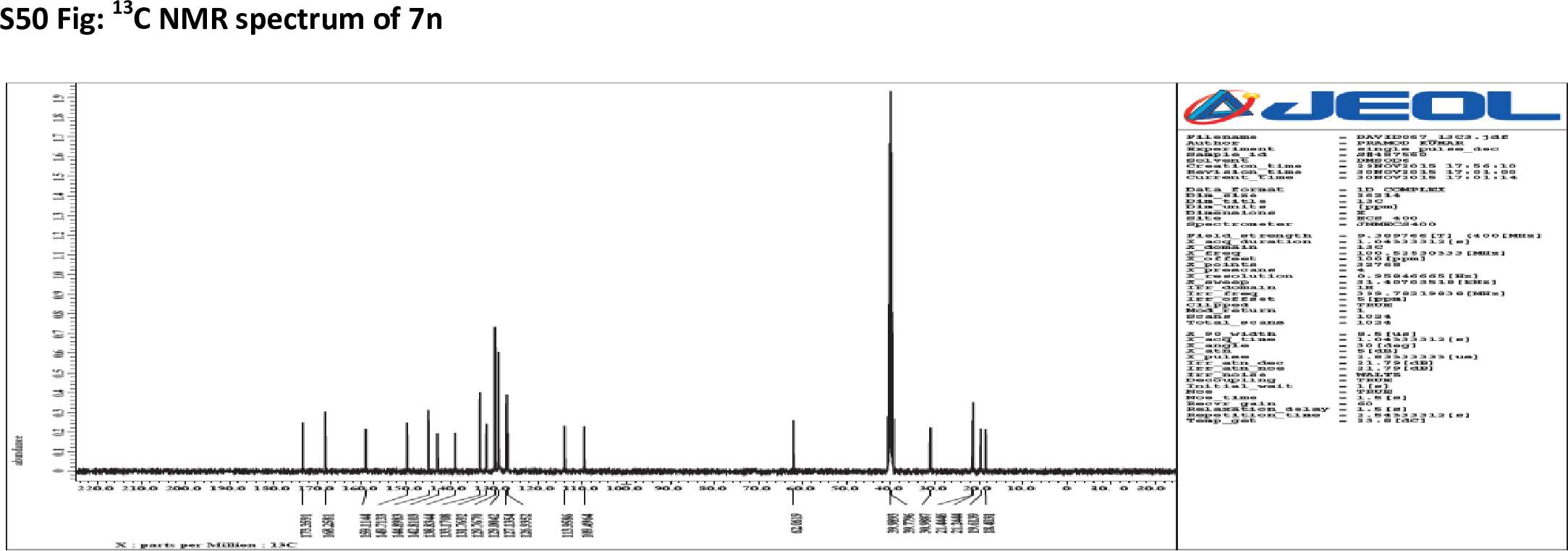

Supplement: S50 Fig — (TIF) [file pone.0183807.s050.tif]

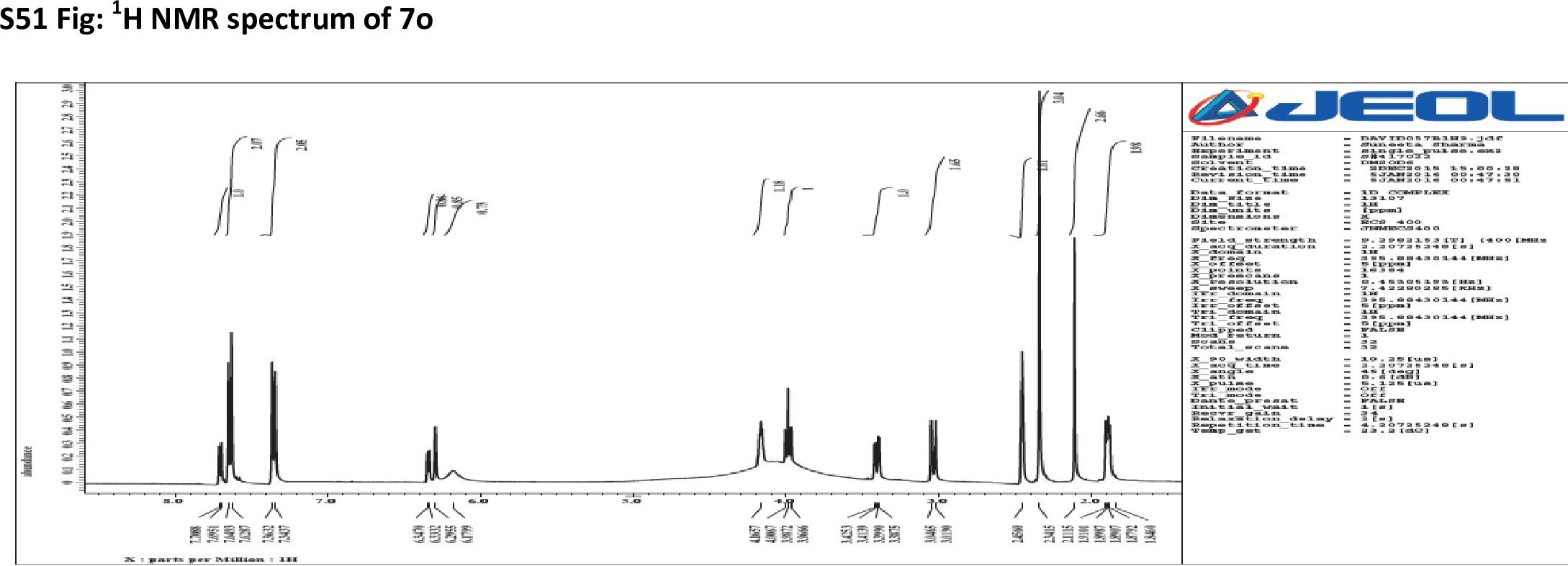

Supplement: S51 Fig — (TIF) [file pone.0183807.s051.tif]

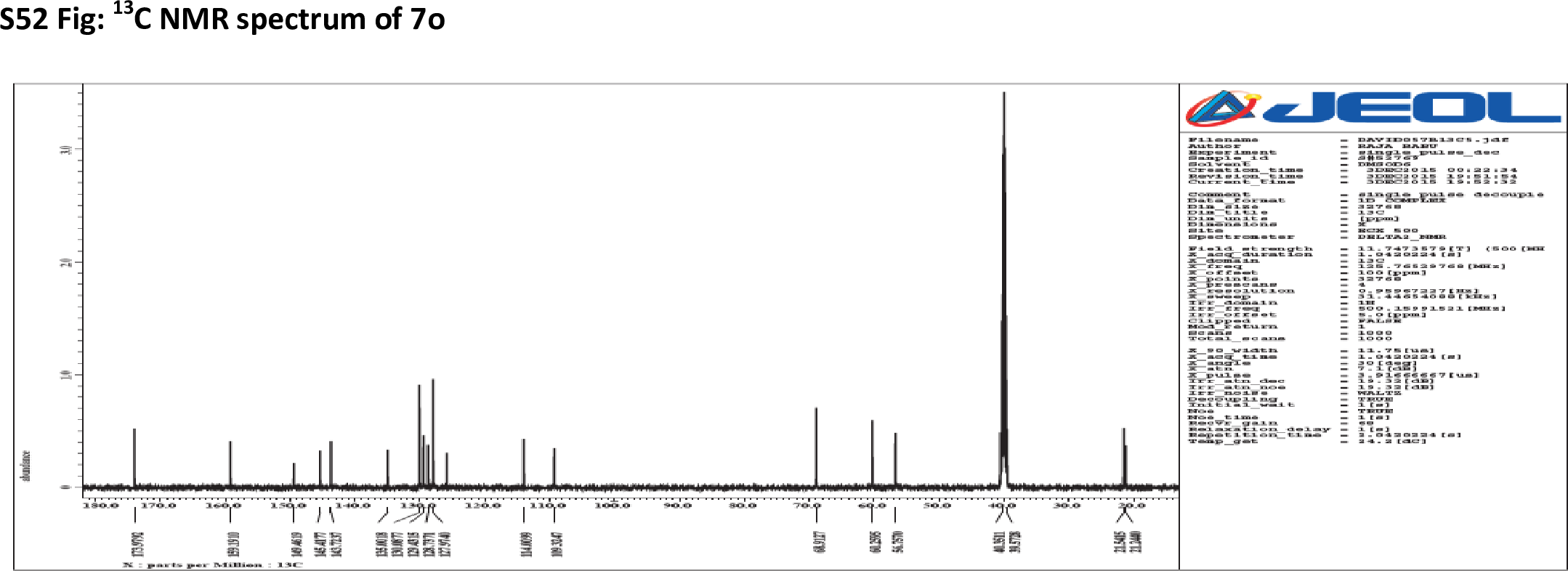

Supplement: S52 Fig — (TIF) [file pone.0183807.s052.tif]

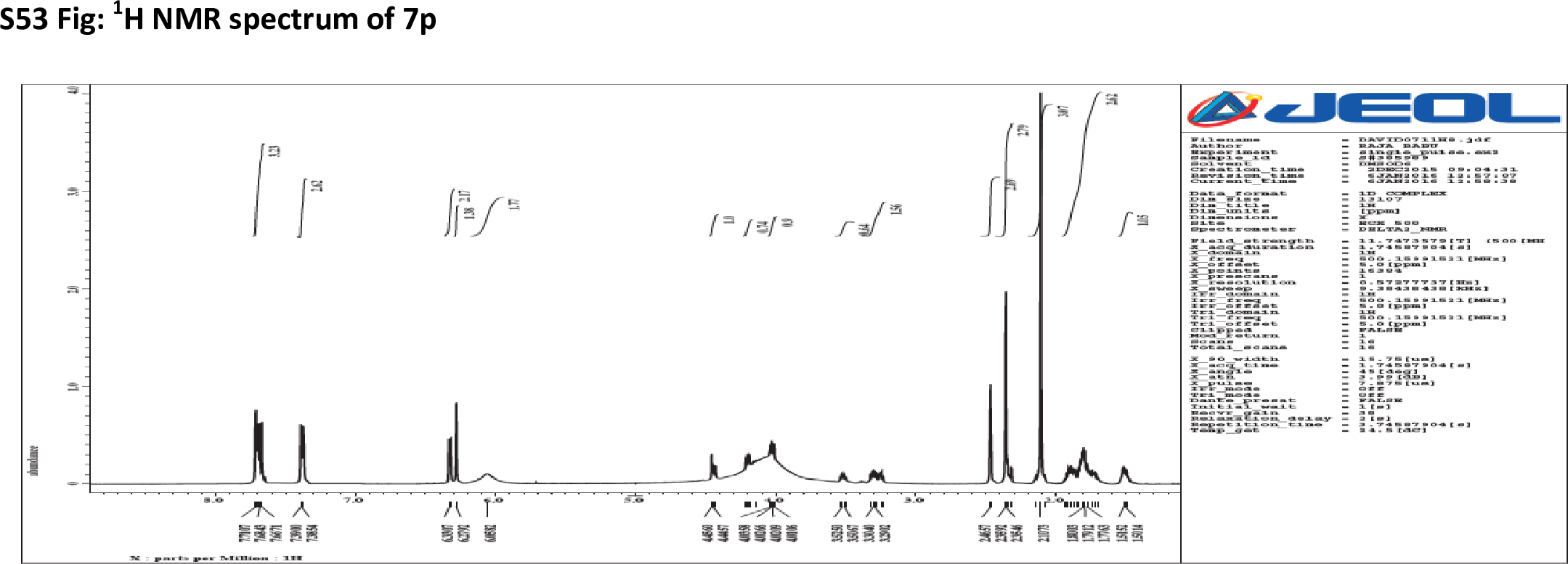

Supplement: S53 Fig — (TIF) [file pone.0183807.s053.tif]

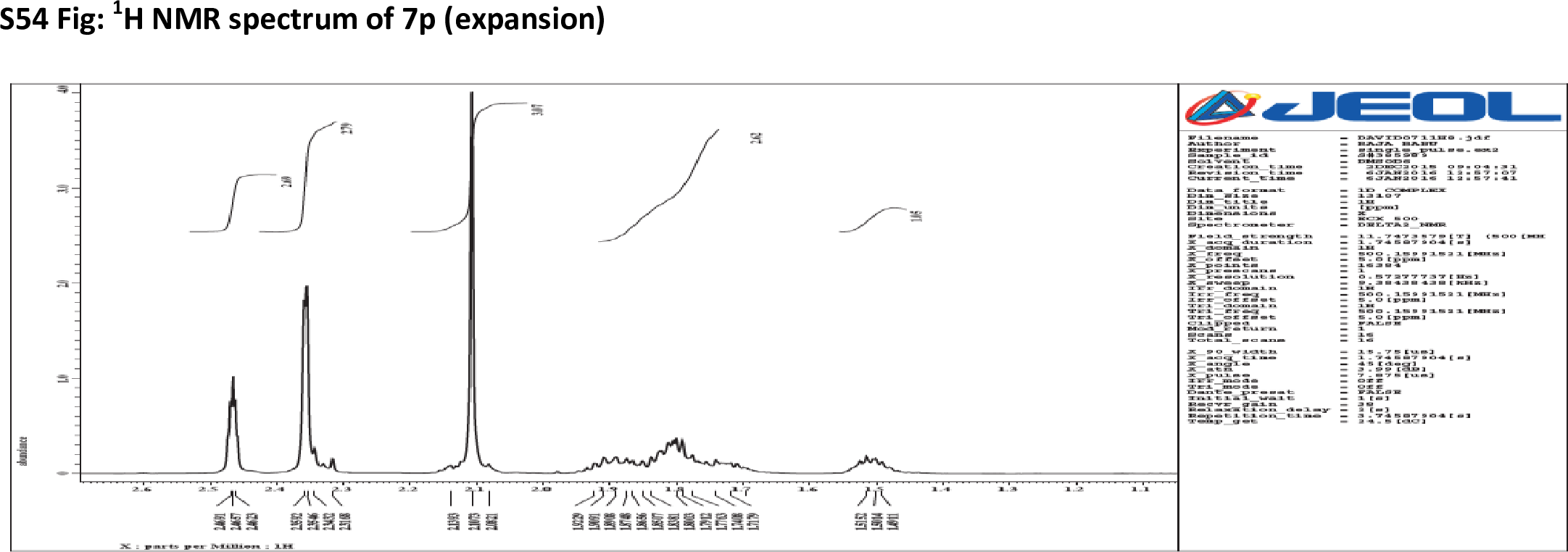

Supplement: S54 Fig — (TIF) [file pone.0183807.s054.tif]

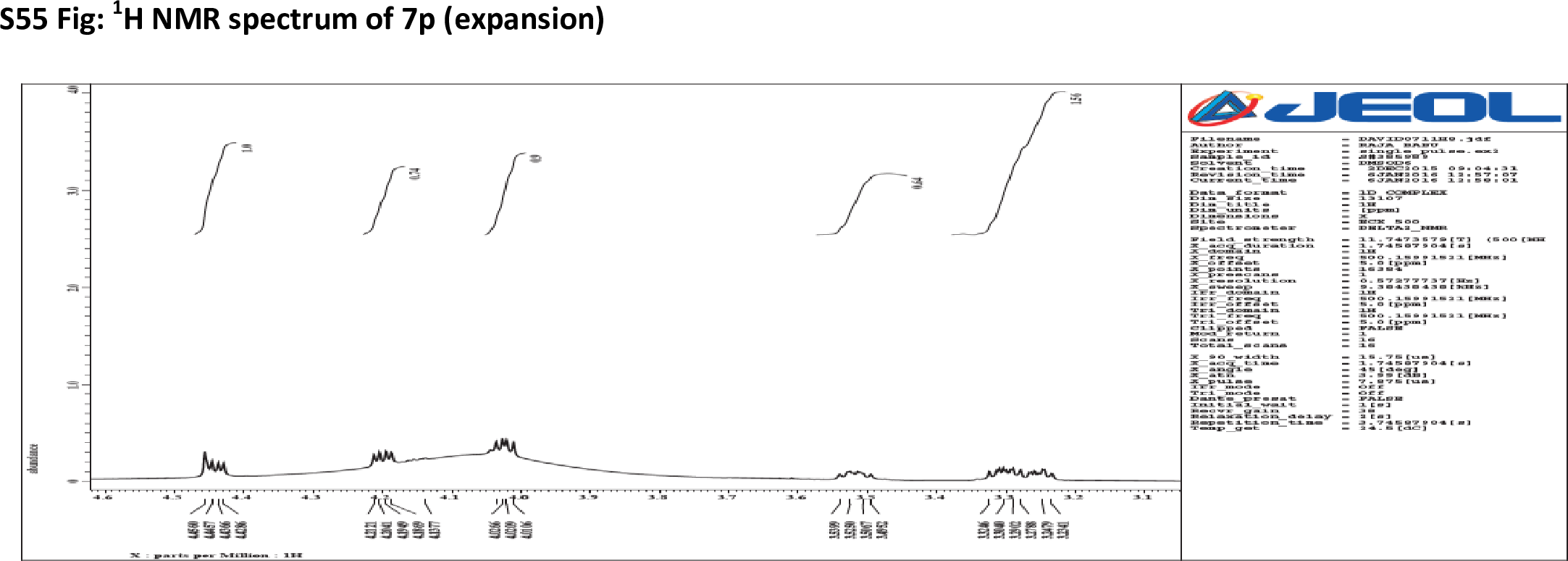

Supplement: S55 Fig — (TIF) [file pone.0183807.s055.tif]

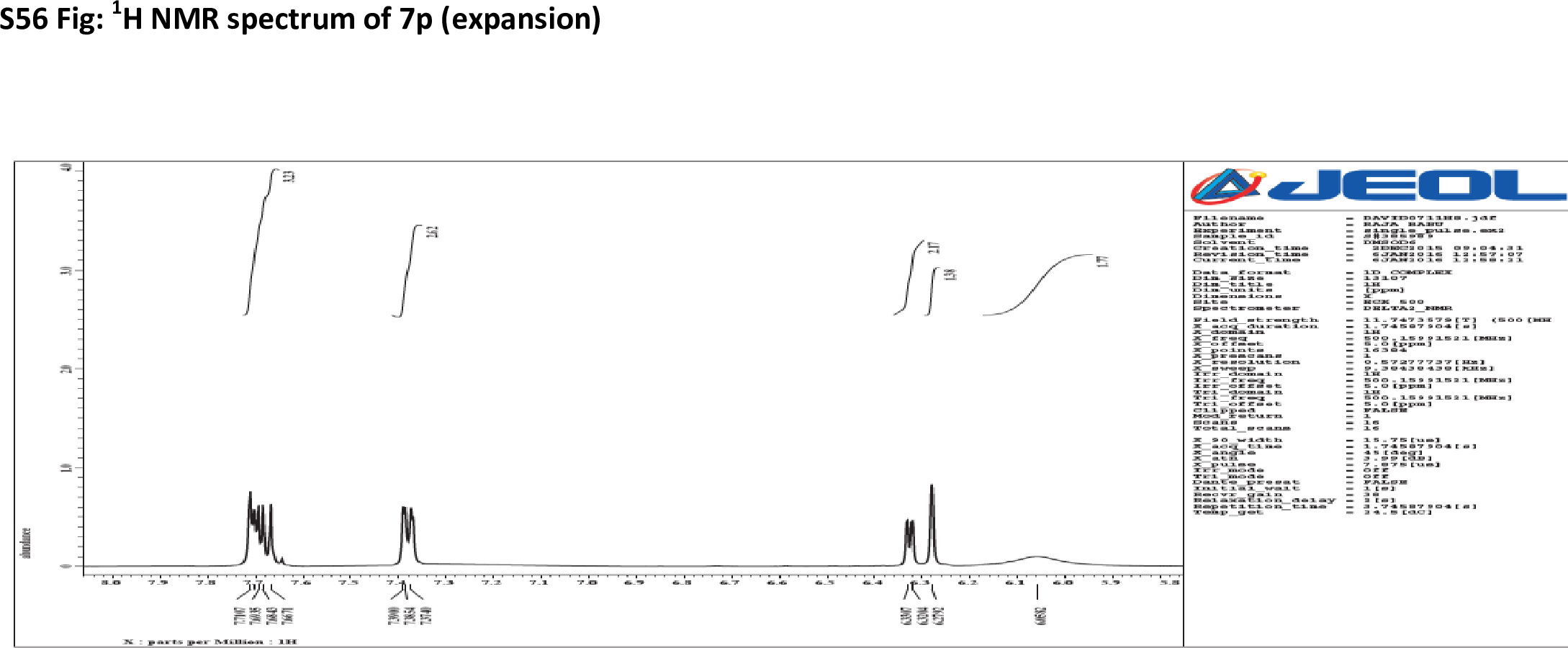

Supplement: S56 Fig — (TIF) [file pone.0183807.s056.tif]

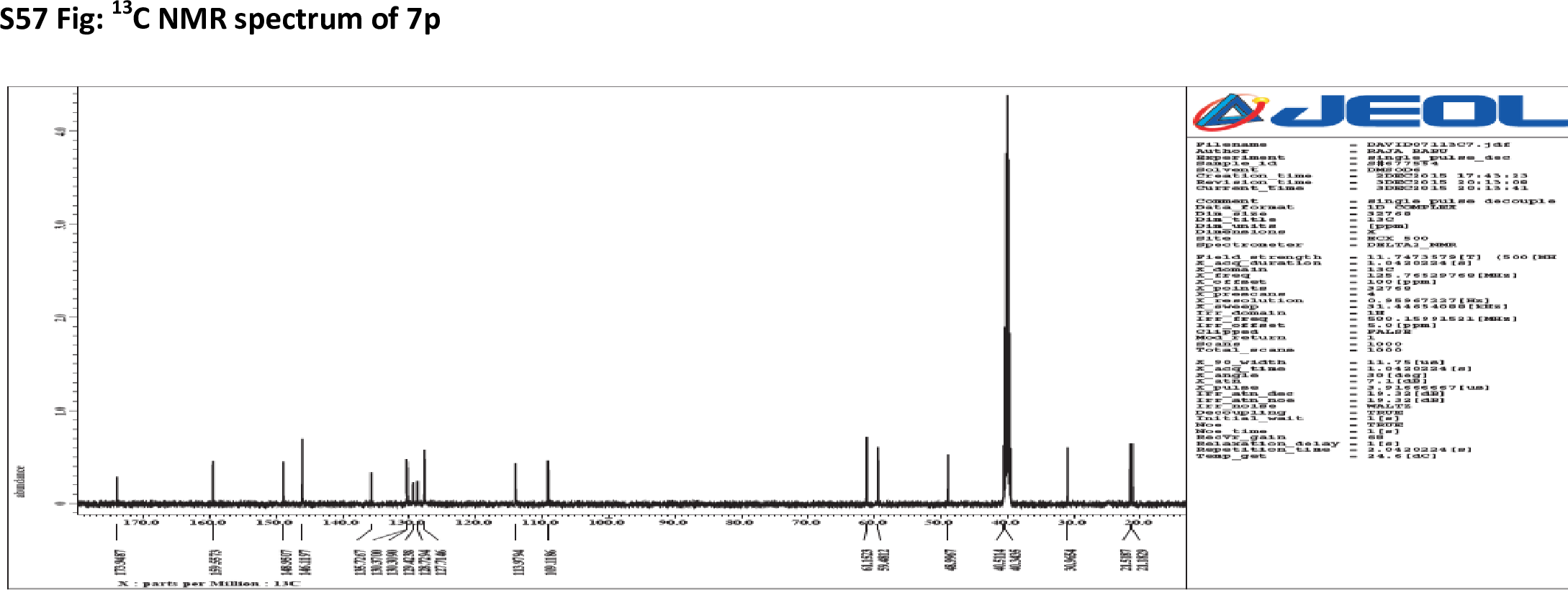

Supplement: S57 Fig — (TIF) [file pone.0183807.s057.tif]
